# Machine learning approach for elucidating and predicting the role of synthesis parameters on the shape and size of TiO_2_ nanoparticles

**DOI:** 10.1038/s41598-020-75967-w

**Published:** 2020-11-03

**Authors:** Francesco Pellegrino, Raluca Isopescu, Letizia Pellutiè, Fabrizio Sordello, Andrea M. Rossi, Erik Ortel, Gianmario Martra, Vasile-Dan Hodoroaba, Valter Maurino

**Affiliations:** 1 Department of Chemistry, University of Torino, Via Giuria 7, 10125 Torino, Italy; 2 UniTO-ITT Joint Lab, via G. Quarello, 15/A, 10135 Torino, Italy; 3 Department of Chemical and Biochemical Engineering, University Politehnica of Bucharest, 1-7 G. Polizu Street, 011061 Bucharest, Romania; 4 Department of Quality of Life, Istituto Nazionale Di Ricerca Metrologica (INRIM), Strada delle Cacce 91, 10135 Torino, Italy; 5 Division 6.1 Surface Analysis and Interfacial Chemistry, Federal Institute for Materials Research and Testing (BAM), Unter den Eichen 87, 12205 Berlin, Germany

**Keywords:** Nanoscale materials, Synthesis and processing

## Abstract

In the present work a series of design rules are developed in order to tune the morphology of TiO_2_ nanoparticles through hydrothermal process. Through a careful experimental design, the influence of relevant process parameters on the synthesis outcome are studied, reaching to the develop predictive models by using Machine Learning methods. The models, after the validation and training, are able to predict with high accuracy the synthesis outcome in terms of nanoparticle size, polydispersity and aspect ratio. Furthermore, they are implemented by reverse engineering approach to do the inverse process, i.e. obtain the optimal synthesis parameters given a specific product characteristic. For the first time, it is presented a synthesis method that allows continuous and precise control of NPs morphology with the possibility to tune the aspect ratio over a large range from 1.4 (perfect truncated bipyramids) to 6 (elongated nanoparticles) and the length from 20 to 140 nm.

## Introduction

Titanium dioxide is one of the most studied semiconductor metal oxides, due to its surface and electronic properties providing multisectorial applications that range from healthcare, photocatalysis, smart materials with self-cleaning and self-sterilizing properties and solar energy harvesting (photovoltaics and water photosplitting)^1–5^. However, it is difficult to correlate the functional properties of TiO_2_ nanomaterials to the properties at single nanoparticle (NP) level, due to broad size dispersity, the complex morphology and, consequently, the differences in surface properties of material used. In the last decades, intensive experimental and theoretical studies have been conducted on the reactivity of different metal oxides such as TiO_2_ as a function of the crystal surface exposed and morphology^1,2,6–14^. The request for different particle shapes, and therefore different surface terminations, stems from the different physico-chemical properties associated to different surfaces, including surface states, which can confer different reactivity and interfacial properties to the material. The possibility to tune the crystal morphology could have a strong impact for several applications, e.g. in photocatalysis the morphology can guide the charge carriers' dynamics^15^, while in the field of nanocomposites the tribomechanical properties could be adjusted^16–19^. Nonetheless, the availability of NP materials displaying uniformity at the nanoscale would be beneficial to tune product performance. Moreover, another aspect that should not be overlooked is the possibility to use size and shape-controlled nanoparticles (NPs) with high homogeneity and high stability (both of morphology and of the colloidal suspension) as a reference material for dimensional nano-metrology of real-world, non-spherical NPs. Currently, there is scarcity of certified reference nanoparticles. The few ones, only Au and silica, are rather model nanoparticles, i.e. spherical, monodisperse and generally non-overlapping when prepared on a substrate. The real world of industrial nanoparticles implies complex particle shape, a certain degree of agglomeration/aggregation state, size polydispersity. The nanoparticles presented in the present work constitute a milestone for the reliable but challenging analysis of size and more complex shape of real-world nanoparticles^20–22^. The synthesis of well-defined and stable NPs as reference materials offers a solid basis for the development of procedures and models to improve the traceability chain, compatibility and comparability of nanoparticle size measurements. This is key for further development in the standardisation of accurate size measurement for non-spherical nanoparticles^22^. The prediction of final shape and size of a nanocrystal from the synthesis conditions starting from first principles it is currently unfeasible. Under thermodynamic control, the final shape is related to the surface energy of the various crystallographic surfaces. However, these are controlled by the presence of adsorbates that can act as shape controllers^23^. Concerning anatase synthesis under hydrothermal (HT) conditions, there are several experimental and theoretical studies that suggest various mechanism of TiO_2_ crystal growth for HT processes^24–27^. Recently, we proposed a model able to describe the evolution of crystal size and shape during alkaline HT processing of triethanolamine-titanium (IV) complex^27^. However, a quantitative model able to predict the size and morphology from HT synthesis conditions still does not exist. In this context Machine Learning (ML) is increasingly used for predicting the relationship between determined input parameters and resulting structures or properties from available datasets, without using first principles^28–31^. Although certain works were already carried out in several fields like synthesis, catalysis, molecular interactions, biology, engineering, etc.^32–38^, often ML is used to establish the relationship among a structure and a single determined material property only, rather than optimize the conditions to maximize the efficiency of a process^39–42^. Less frequently, it is used for the material design, i.e. the study of the influence of synthesis parameters on the final morphology of the NPs; and where this has been done, only one final morphological characteristic is taken into account (size or shape)^37,43^. This occurs because ML approaches are limited by the lack of large pre-existing coherent data sets, especially with regards to material synthesis^32^. The problem can be partially overcome by using experimental design techniques, which are often employed for building and optimizing regression models able to determine efficiently the set of conditions necessary to obtain a product or process with desired characteristics^44–48^. The combination of experimental design and ML could allow building a model able to predict/optimize the process parameters even in the presence of a reduced initial data set^49–54^. Also failed experiments can be useful for this task^55^. Further, there are recent studies demonstrating the use of ML for classification of materials (or corresponding data)^56^. Besides the valuable prediction of advanced material properties such as physical, chemical, optical, mechanical, electrical etc., i.e. material discovery, the idea of high-throughput computational materials research is another promising application of ML^57^.

In the present work we present a quantitative model for the synthesis of TiO_2_ NPs with “a priori” established shape and size and limited polydispersity. To this aim, we coupled experimental design and ML techniques for the material synthesis, starting from a known HT synthesis method, for which the growth mechanism was investigated^27,58^. The syntheses of truncated bipyramidal (the equilibrium shape for anatase)^1^ and elongated anatase NPs were performed using as precursor a titanatrane [Ti(TeoaH)_2_] (see characterization in Supplementary Information SI, Sect. 2), and triethanolamine as shape controller exploring a quite wide experimental domain, in terms of reaction pH, temperature and reactants concentrations. Then, a mathematical model was developed to generalize and predict NP shape and size. Modelling was carried out in an iterative way with Artificial Neural Network (ANN) and Genetic Algorithm (GA) for the optimization, in the framework of the learning approach^59–63^. This enabled the formulation of optimized models, which is able to guide the syntheses towards materials constituted of low polydispersity NPs with desired shape and size in an inverse engineering approach. This tool allows identifying well-defined parameters of synthetic procedures once certain particle size and shape are required for a particular application. Moreover, the procedure could be extended to other oxides and materials^64^. The choice of ANN for process modeling, despite the small dataset, is due to their capability to fit data, without overfitting, which make them reliable, especially for nonlinear dependency between variables, which is our case. ANNs gave better results for some extension of the variables’ variation range resulting in a higher prediction power than classical polynomial regression, even though large datasets are required^65^. The work follows step by step the creation of the ANN models using the data obtained by the experimental design and their refinement and optimization with the increase of the dataset with further experiments. For each step, a comparison with a second degree polynomial regression was carried out in order to estimate the goodness of the ANN models.

**Chart 1:**
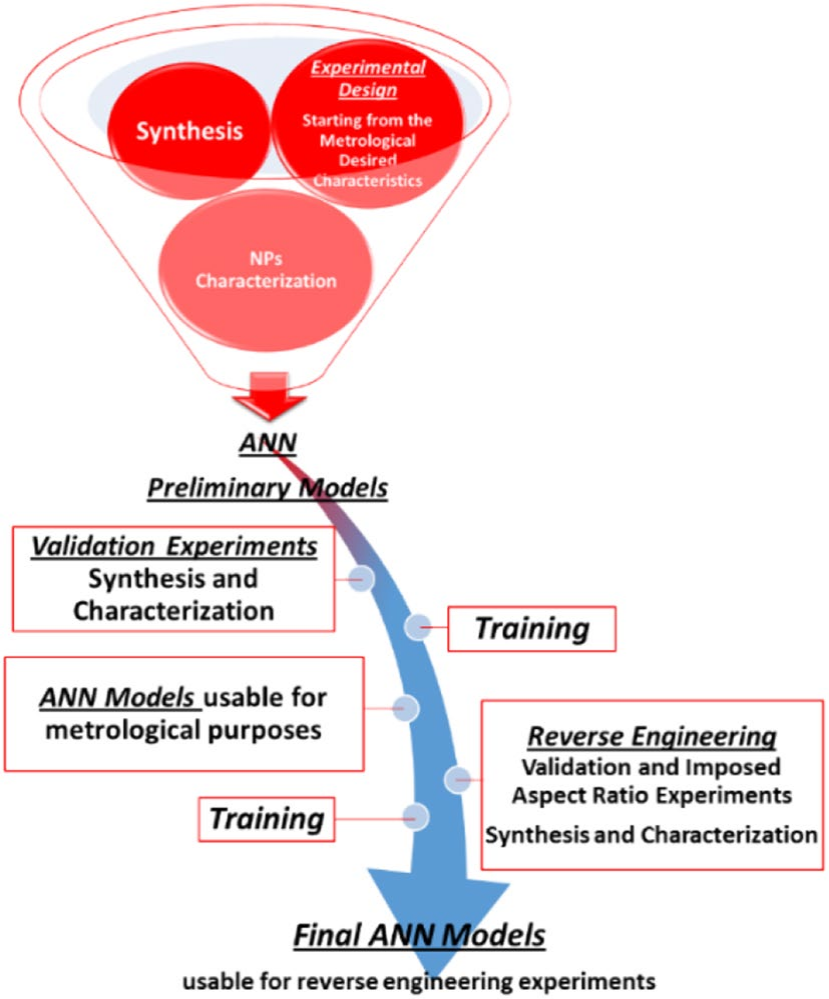
Flowchart of the work process, from the experimental design to the validated and optimized ANN models.

Chart 1 depicts the rationale of this work. The first part is dedicated to the experimental design (defining the desired product characteristics) and the complete morphological characterization of the NPs. The second part describes the development of the ML procedures (in particular ANN modelling) that predict the final characteristics of the NPs, together with their optimization (throughout Genetic Algorithm, GA) and the reverse engineering processes.

## Results

### Experimental design and synthesis results

The experimental design technique employed is based on the Responses Surface Method, by using Box Wilson central composite design (CCD)^47,66^. In SI (Sect. 5), we report the complete explanation of the Experimental Design construction. The experimental plan considers the four independent variables (factors Z_1_ to Z_4_), which mainly influence the characteristics of the product:

- Z1—[Ti(TeoaH)_2_] initial concentration;
- Z2—Added TeoaH_3_ concentration as shape controller;
- Z3—Initial pH;
- Z4—operating temperature.

The experimental ranges studied were from 30 to 120 mM [Ti(TeoaH)_2_], from 0 to 70 mM added TeoaH_3_, pH between 8.7 and 12 and temperature values from 135 to 220 °C. The orthogonal fractional CCD is shown in SI (Table S2). The required values for the products characteristics, Y_1_ to Y_3_ are:

- Hydrodynamic radius (Y1) → R_H_, max. 25 nm; the choice of the hydrodynamic radius evaluation was done because this parameter allows determining more accurately the polydispersity of NPs. Moreover, R_H_ can be rapidly determined by DLS measurements and then directly compared with the electron microscopy analysis through the Perrin equation (details in SI, Sect. 1). Obviously, R_H_ were determined on stable suspensions and following a standard operative procedure for all samples;
- Polydispersity (Y2) → max. 5%, corresponding to 1 nm for Y1 values around 20 nm; the experimental results are expressed as standard deviation (in nm) of the DLS hydrodynamic radius distribution for the relevant mode; for mathematical analysis Y2 is converted in percentages;
- Aspect Ratio (*p*) → required value is 1.5 representing the ratio between major and minor axes of the ellipse that fits to the particle boundaries (for details see Sect. 1—page 3 in SI). The ellipse model for fitting the bipyramids has been chosen due to simplicity (and practicability) reasons.

The experimental program adopted allows the investigation over a large range of operating parameters using a limited number of experiments (Table S2). This will give the possibility of process analysis and mathematical modelling in order to predict final product characteristics in various operating conditions as well as to identify the synthesis conditions for a product with predefined final properties.

**Figure 1:**
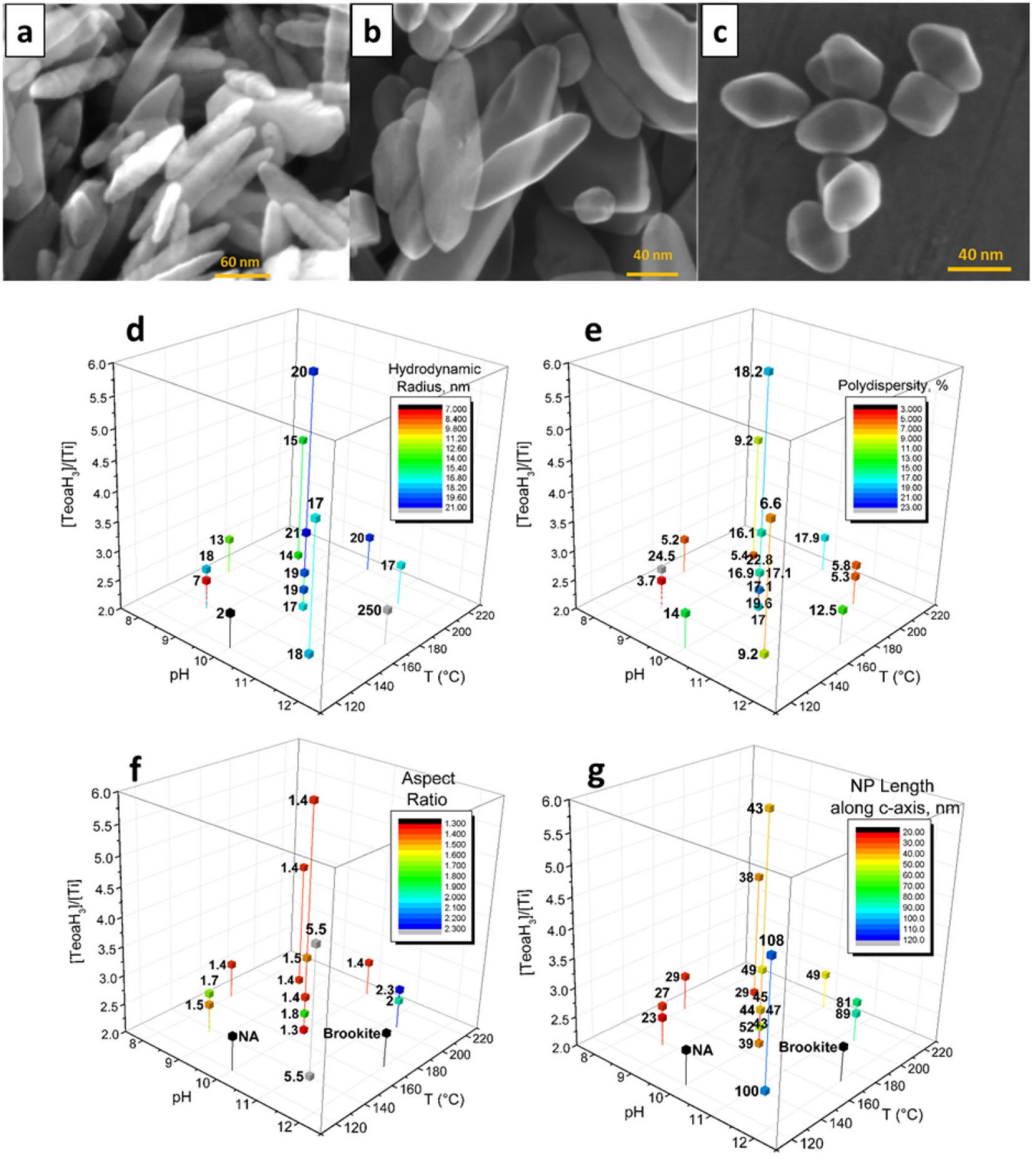
High-resolution SEM micrographs of selected synthetized materials: HT06 (**a**), HT08 (**b**), and HT16 (**c**). 3D-plots of Hydrodynamic Radius (**d**), Polydispersity (**e**), Aspect ratio (**f**) and NP length along the c-axis (**g**) experimentally obtained as functions of pH, Temperature and [TeoaH_3_]/[Ti] relative concentration. The data reported represent the 20 materials obtained following the experimental design.

The results of the experimental design, i.e. microscopy measurements, XRD and DLS are described in detail in SI (Sects. 6–8) and summarized in Fig. 1 (together with HR-SEM micrographs of three materials synthetized), where it is possible to gather further insight into the relevance of HT synthesis parameters. The NPs synthetized are characterized by a different elongation along the *c* axis of the anatase crystal (23–108 nm) (Fig. 1g, Table S3 and Sect. 7 of the SI). The elongation is favored by high pH values in the whole temperature range considered. Very low hydrodynamic radii are expected at low pH and low temperature conditions, in a narrow region of the experimental ranges explored here, while particles with hydrodynamic radius between 10 and 20 nm can be obtained in several working conditions (Fig. 1d, Table S4 and Sect. 8 of the SI). In the whole range of temperatures and pH values, for 65 mM Ti(TeoaH)_2_ and 40 mM added TeoaH_3_, the expected R_H_ would never exceed 20 nm. For higher Ti(TeoaH)_2_ loadings and higher added TeoaH_3_ concentrations there is a large region in the pH and temperature variation range where particles with R_H_ larger than 20 nm may be obtained. However, even in this case, particle hydrodynamic radius would be limited to 29 nm. As for the hydrodynamic radius, aspect ratios between 1 and 2 are expected in a very wide region of working conditions, whereas high max/min values are favored at initial pH between 11 and 12. In that pH range, the aspect ratio increases with decreasing temperature. Nevertheless, there is a complex interplay among the variables considered that impedes to find other empirical relationships. The polydispersity (%) of NPs is almost always larger than the recommended for metrological applications (5%) (see Table S4), however it must be taken into account that with such complex morphology, polydispersity is even more challenging to be measured accurately, especially for high aspect ratios.

### Artificial neural networks (ANN) modelling

ANN modelling was carried out for the factors’ screening and the analysis of their relative influence upon TiO_2_ NPs synthesis by HT method. Details about the ANN formulation are presented in Supplementary Information (see Sect. 10).

**Figure 2:**
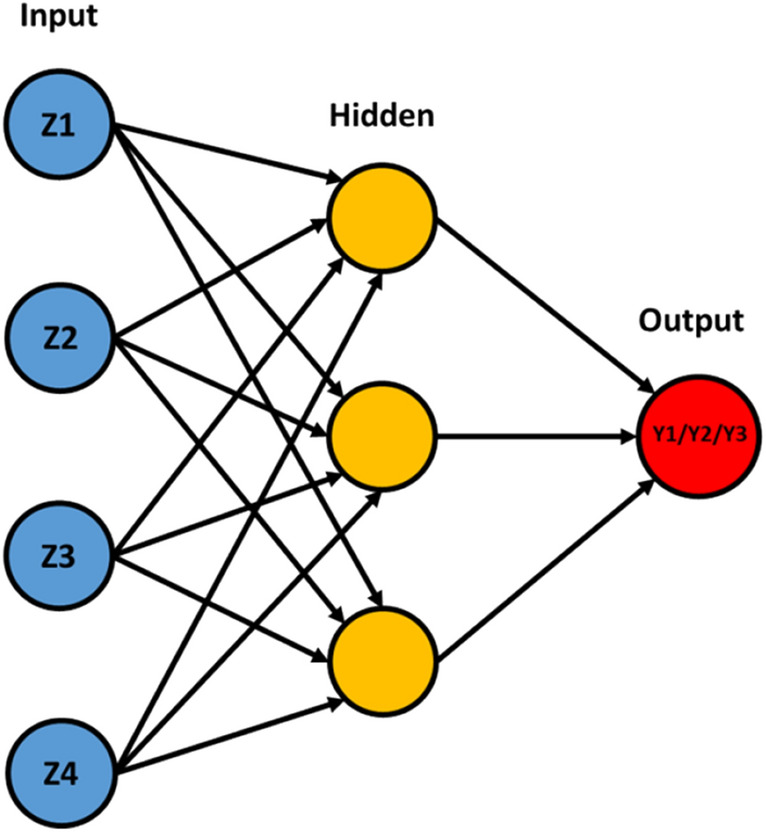
Architecture of the used ANN models, with Z1 to Z4 selected as input neurons and Y1 to Y3 defined as output neurons.

Three distinct modelling ANN were built to reflect the influence of working parameters upon the characteristics of the bipyramidal anatase synthetized. All three networks have the same architecture: four input neurons, for the four independent variables (Ti(TeoaH)_2_ concentration (mM), added TeoaH_3_ concentration (mM), pH and temperature), three neurons in the hidden layer to ensure learning capability, without increasing the number of ANN weights too much, and one output neuron, for the output variable to forecast (the hydrodynamic radius (Y1), the polydispersity (Y2) and the Aspect Ratio (Y3), respectively, Fig. 2). The ANN has 15 total interconnections, avoiding overfitting problems. In this study, the activation function is the sigmoid, and learning algorithm is back-propagating. For each of the three ANNs, from the 20 experimental data, 16 data sets were used for training and 4 data sets for validation and testing. The ANNs were built in the frame of Matlab R2015a software (Math Works, Natick, MA, USA).

ANN modelling of hydrodynamic radius, polydispersity and aspect ratio were compared at each step with second degree polynomial regression models developed by using the same dataset (see Sect. 9 of the SI for details). In Table 1, we reported the mean relative error of the developed models for hydrodynamic radius, polydispersity and aspect ratio. Table 1 highlights that for the three parameters taken into account, ANN models have always a better prediction ability.

**Table 1:** Comparison between the relative mean errors for second degree polynomial models and ANN models.

ANN (20 experiments)			Polynomial (20 experiments)		
R_h_	Polydispersity	Aspect ratio	R_h_	Polydispersity	Aspect ratio
2.4%	9.9%	2.6%	12.0%	18.0%	2.9%

Based on the trained ANNs, the influence of process parameters upon the characteristics of particle size distribution (mean hydrodynamic radius, polydispersity) and aspect ratio was investigated. Each network was used to predict the behavior of the system for the experimental range of the operating parameters. The simulations (reported in Figs. S88-S95, SI) revealed that the factors mostly influencing the characteristics of the final product are the pH and the temperature (in this order). These results are in good agreement with the empirical experimental observations.

The validation of the mathematical model was realized by performing 6 new experiments in the range of interest for the operating parameters (Table S5). The experimental outcomes for the obtained TiO_2_ NPs were compared with predicted ones. The data in Table S6 show a relatively good model prediction capability. In particular, the ANN models mean relative error is better compared with the polynomial models for the prediction of polydispersity.

### Refining of the ANN models with the addition of validation experiments

In order to improve the prediction ability of the neural networks, the six validation synthesis were added to the initial 20 experimental points. The neural networks defined for Y1, Y2 and Y3 calculations presented in the previous paragraph were tested for the new set of experimental data (HT-MODEL 01-06). Maintaining the same structure, the ANNs were further trained by using the whole new data set of 26 experiments. Once again, the match of the Polydispersity is the Y parameter suffering from largest deviations. This could be explained by the challenge in measuring accurately the polydispersity index for such non-ideal samples. Table 2 reports the mean relative errors of the developed models (ANN vs Polynomial) for hydrodynamic radius, polydispersity and aspect ratio.

**Table 2:** Comparison between the relative mean errors for second degree polynomial models and ANN refined models.

ANN (26 experiments)			Polynomial (26 experiments)		
R_h_	Polydispersity	Aspect ratio	R_h_	Polydispersity	Aspect ratio
3.0%	12.7%	2.9%	11.4%	22.5%	4.0%

Table 2 highlights that the ANN models have a significantly higher prediction ability compared to the polynomial regression models.

### Reverse engineering ANN models

Reverse engineering is defined as a technique that starts from the product and works through the design/synthesis process in the opposite direction to arrive at product definition statements. Extending this concept to the synthesis of titania NPs, the aim of reverse engineering approach is to find out the particular synthesis conditions enabling to obtain a product with defined R_H_, polydispersity and aspect ratio. This goal may be realized using mathematical modelling tools and optimization procedures. Therefore, ANN capability to capture the relation between operating condition and final product characteristics was used:

- to directly find the corresponding working conditions knowing the product characteristics;
- to define a functional dependence on working conditions (X) for each product characteristic (Y).

The optimization tool used is the Genetic Algorithm (GA). This algorithm is inspired from natural evolution and provides a heuristic search of the response surface, identifying the regions where optimum values may be located. Due to their working principle, GA is more robust in the localization of global optima than hill-climbing methods and it is expected to give good results in the case of multimodal objective functions, as it is the case in the present study. The search for best operating conditions for given product characteristics was achieved by formulation of an optimization problems. The definition of the goal was realized in several ways and is reported in SI (Sect. 10). The objective functions were selected based on the cumulated product requirements: minimum mean hydrodynamic radius (Y1), polydispersity (Y2) lower than 5% and Aspect Ratio (Y3) very close to 1.5 corresponding to low truncated anatase NPs. The optimized models were used to estimate the influence of operating parameters on the product characteristics and to propose optimal operating condition for predefined final product characteristics. Based on these predictions five additional validation experiments for the reverse engineering models were carried out together with three imposed aspect ratio experiments, in order to increase also the model potential to indicate the process parameters for elongating the NPs. The new experimental data will contribute to yield more reliable ANN models, capable of covering all the desired ranges of the operating conditions for the HT synthesis of anatase NPs.

The data shown in Table S8 highlight that there is a certain discrepancy between the experimental and the predicted results, especially for the materials with imposed aspect ratio. Therefore, a further implementation of the models for reverse engineering problem was mandatory and an ANN analysis exploiting as input also the 8 new experimental results was carried out. ANN and GA provide together a modelling tool for reverse engineering approach of the crystallization process.

The validation experiments proved that the model can estimate very well product characteristics in the range of the experimental data used in defining the ANN model and less accurate outside this region (for example the conditions for aspect ratio 4.2, see Table S8). The new experiments cover almost completely the whole experimental region and they are all used in the next iteration step to build new ANN models that would better predict the product characteristics starting from operating conditions. The validated optimized reverse-engineering models were built using all the experimental data. New ANN models were built for Y_1_, Y_2_, and Y_3_ using all the data obtained from the synthesis of the experimental design and of the validation experiments. The optimal architecture identified for the new ANNs was similar to the initial ones: 4 neurons in the input layer (Ti(TeoaH)_2_ initial concentration, TeoaH_3_ initial concentration, pH and Temperature), 3 neurons in the hidden layer and 1 neuron in the output layer (Y_1_, Y_2_ and Y_3_ respectively). The way in which the optimized models reflect the experimental variation is expressed as relative mean errors percentage in Table 3.

**Table 3:** Comparison between the relative mean errors for second degree polynomial models and ANN final models.

ANN (26 experiments)			Polynomial (26 experiments)		
R_h_	Polydispersity	Aspect ratio	R_h_	Polydispersity	Aspect ratio
9.1%	13.9%	5.6%	11.9%	19.5%	10.1%

Table 3 confirms that final ANN models built with all the 34 experiments present a higher capacity to predict the results of the synthesis compared with the polynomial models.

Again, these new models can be further employed in solving the inverse problem: find out the operating conditions corresponding to given product characteristics. The higher errors with respect to the previous ones (see Tables 1 and 2) is due to expansion of the ranges of the model with the high imposed aspect ratio experiments, leading to a possible distortion of the model. As already commented earlier, the polydispersity of NPs with such complex morphology is even more challenging to be measured accurately for high aspect ratios. However, the models are able to predict reasonably well the aspect ratio (from 1.4 to 6) and the hydrodynamic radius of the NPs. The comparison of all the data of the final refined models are shown in Tables S9 and S13 of the SI.

### ANN model for NPs length along the c-axis direction

Once having seen good results in the ANNs modelling for hydrodynamic radius, polydispersity and aspect ratio, all experimental data were employed for new ANN models, built to reflect the length of the nanoparticles along the c-axis direction dependence on operating parameters (Table S3 column “Major” for the values) and its polydispersity (see SI, Table S10). These new results, unlike R_H_ and polydispersity index, are completely independent on the colloid stability (although the DLS measurements were performed on stable colloids) because in this case the measurands are geometric values obtained from the TSEM micrographs.

**Figure 3:**
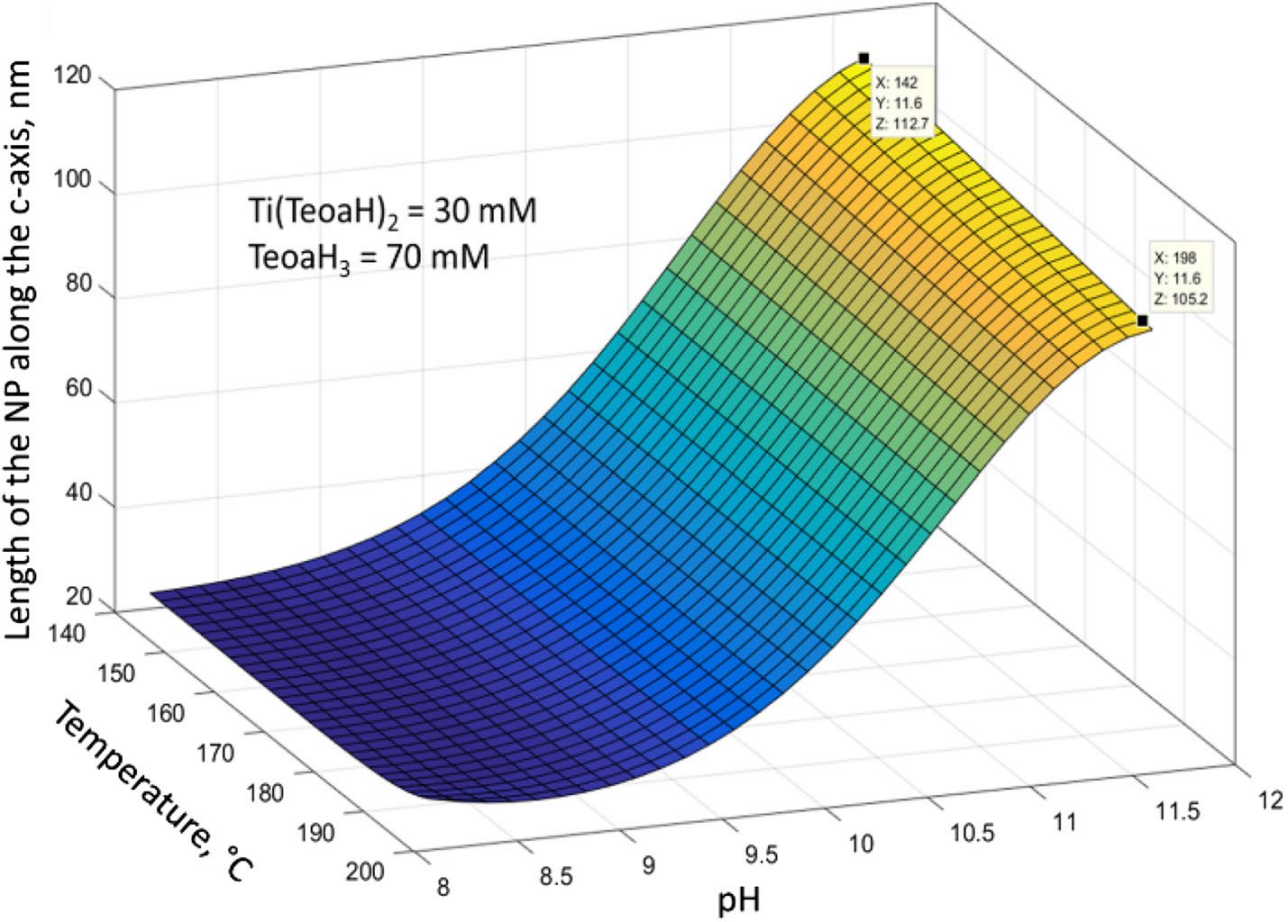
Variation of the length of the nanoparticles along the direction of the c-axis of the crystal as a function of pH and temperature (image created with Matlab R2015a software (Math Works, Natick, MA, USA), https://it.mathworks.com/products/matlab.html).

Table 4 displays smaller error between experimental and computed values for the ANN models. The new ANNs can be employed for performing simulations as reported in Fig. 3, where the variation of the length of the nanoparticles along the direction of the c-axis of the crystal is reported as a function of pH and temperature (with precursor and shape controller concentration fixed to 30 mM and 70 mM respectively), the parameters that mainly influence the NPs morphology.

**Table 4:** Comparison between the relative mean errors for second degree polynomial models and ANN refined models obtained for the length of the NPs along the c-axis and its standard deviation.

c-axis length		Standard deviation	
ANN	Polynomial	ANN	Polynomial
8.5%	13.5%	8.9%	18.1%

It must be underlined, that the length oS3f the NP does not necessarily correspond to the measure of the c-axis intended as average size of crystal domain (D_004_) as obtained from XRD measurement (Table and in detail in the Sect. 7 of the SI). In particular, the length of the NP seems to be comparable to D_004_ only for aspect ratios up to 2 (Table S3).

To test the final reliability of all the ANNs model, they were used for predicting the NPs characteristics of a last synthesis, obtaining very good results for almost all the ANN models (Table S12) and each time they performed better compared with the polynomial models. Obviously, each ANN model can be validated with new experiments that consequently can be used to the refine model improving its prediction ability, resulting in an iterative process that can gradually lead to improved reliability.

## Discussion

The TiO_2_ NPs obtained in the experimental design used in the present study have an aspect ratio ranging from 1.4 to 5.5 with sizes from 20 to 110 nm. The hydrodynamic radius values measured with the DLS (R_H_ 7–20 nm) are in fair agreement with the R_H_ values derived from the Perrin formula (R_H_ 9–29 nm), which takes into account the TSEM analysis. Moreover, the comparison of the average size of the crystal domains (D_004_) obtained by Scherrer Analysis of the 004 XRD peak (c-axis) with the actual length of the nanoparticles obtained by electron microscopy (see Table S3 of the SI) confirms the low defectivity of the nanoparticles along the c-axis for almost all the materials synthetized. The lower D_004_ values obtained for the more elongated nanoparticles (aspect ratio higher than 2) compared with the nanoparticle length obtained from the microscopy, indicates a higher defectivity of the crystal lattice of these NPs and the possibility to have poly-crystallinity. These new findings further highlight the difficulty to obtain a real strong elongation of the crystal along the c-axis with the exposition of high-energy surface and pay attention to the importance of characterizing nanoparticles in the best possible way before drawing hasty conclusions relying solely on morphology. The final results represented in Fig. 1 highlight some general rules for these kinds of synthesis, in particular: lower pH and temperature conditions lead to lower R_H_. Alkaline pH (> 11) and low temperature favor the formation of brookite phase and a stronger elongation of the NPs.

The results of the experimental design were used as training set for ANN models to predict the TiO_2_ NPs size and shape as a function of four HT synthesis process parameters: pH, temperature, precursor and shape controller concentrations. The models were validated with a dataset constituted of 6 validation experiments. The resulting ANN models were used (together with GA) to formulate optimizing problem and propose best operating conditions for given product characteristics. The ANN models were then reverse engineered, to directly find the corresponding working conditions given the NPs characteristics. The models were tested with other experiments that allow the ranges extension, in particular driving the synthesis of NPs with high aspect ratio. This last models lead to a lower prediction accuracy compared with the previous optimized models, but it allows tuning the elongation of the NPs along the c-axis starting from defined synthesis conditions with good predictive capability, especially for aspect ratio > 3. At each step, the ANN models were compared with a second degree polynomial models employed as benchmark for these kind of analysis. Every time, the performances of the ANN models after refinement exceed (in a more or less marked way) the polynomial ones, confirming the good prediction ability of the ANN models (see Tables S9, S12, S13 and S14).

The ANNs built can be further trained with other experiments, in an iterative process that could lead to closer match between the a priori given and experimentally obtained NPs characteristics. In fact, the neurons are usually trained with thousands of input data in its common applications such as data mining, etc. In this work we have seen how ANN can be successfully exploited to predict the morphology of the NPs even starting from a relatively small data set, but obtained through experimental design. The final test confirms the reliability of the model (always better than polynomial models), with the possibility to continuously control the aspect ratio of the anatase NPs from 1.4 to 6. Finally, new ANN models were built in order to reflect the dependence of the length of the nanoparticles along the c-axis and its standard deviation from the synthesis parameters. The new ANNs can be successfully used to modelling and predict (together with GA) the length of the NPs in wide range from 20 to 140 nm.

## Conclusions

In conclusion, the statistical analysis of data, model building and refining can help to control the product characteristics. By statistical modelling both polynomial and ANN models are good candidates, but with a slight increase of data the prognosis capacity of ANN is better especially when the process has strong nonlinearities.

The design rules obtained in this study thanks to ANN models are valuable per se, because the NP materials of this kind can find large-scale application in catalysis and energy harvesting, but, more importantly, because these results can be extended to different synthesis procedures or to other materials, e.g. α-Fe_2_O_3_ ZnO, WO_3_, etc., thus, widening the experimental ranges and adding new relevant process parameters. In all these cases, the possibility to rely on materials composed of NPs with low polydispersity in shape and size, enables the more reliable correlation between macroscopic properties and morphology at the nanoscale, with significant benefits for both fundamental and applied research.

## Methods

### Nanoparticles synthesis

The NPs were synthetized using a known procedure. The required mass of precursor, a complex of Ti with triethanolamine (details in SI), was dissolved in ultrapure water together with the right amount of triethanolamine as capping agent. The pH was adjusted with 1 M carbonate-free NaOH or 1 M HCl, as required. The final solution was then filtered and heated in a Teflon lined stainless steel high pressure autoclave at the set temperature after a N_2_ sparging of 5 min. Details of the precursor and NPs synthesis and characterization in the Sects. 1–4 of the SI.

### Experimental design

The experimental design technique used is a Box Wilson central composite designs (CCD) with 4 factors (*n*), 8 star points and 4 center points (*N*_*0*_). The experimental plan considered the four independent variables (factors) that mainly influence the product characteristics: Ti (TeoaH)_2_ initial concentration (Z_1_), TeoaH_3_ concentration added as shape controller (Z_2_), initial pH (Z_3_), operating temperature (Z_4_). The output variables are hydrodynamic radius (Y1), polydispersity index (Y2) and the aspect ratio (Y3). Details of the experimental design building in Sect. 5 of the SI.

### Mathematical modelling

The best architecture identified for all the ANNs is: 4 neurons in the input layer corresponding to the operating parameters, 3 neurons in the hidden layer and 1 neuron in the output layer. The ANNs were trained in the frame of Matlab R2015a software using backpropagation algorithm as detailed in SI. The hyper-parameters in ANN are the “by default parameters” given in Matlab for the optimization function chosen. The errors (total errors) are explicitly presented by parity plots and detailed in SI. The criterion for adjusting the weights (optimization of the network structure) was the minimization of sum of squared error in the training step. The data sets were used 70–80% for training, and 30–20% for validation and testing to prevent overfitting and test the prediction capability. The ANN were trained and tested in the frame of Matlab R2015a software. The implemented training algorithm, trainlm is used. It is the fastest backpropagation algorithm in the Matlab toolbox and it updates the weight and bias values according to Levenberg–Marquardt optimization. The other hyperparameters are: Number of epach: max 1000, Maximum validation failures 6; Minimum performance gradient 1e-7, Initial Mu 0.001, Mu increasing factor 10, Mu decreasing factor 0.1, maximum Mu 1e10. For the details of the ANN theory and on all the developed ANNs see the Sect. 10 of the SI.

## Supplementary information

Supplementary Information.
