# Supplementary material for: Machine learning approach for elucidating and predicting the role of synthesis parameters on the shape and size of TiO2 nanoparticles
Source: Sci Rep. 2020 Nov 3;10:18910. doi: 10.1038/s41598-020-75967-w (PMC7609603; doi:10.1038/s41598-020-75967-w)
Supplement: Supplementary file 1 — Supplementary Information. [file 41598_2020_75967_MOESM1_ESM.pdf]

Supplementary Information

Machine Learning Approach for Elucidating and Predicting the Role of Synthesis Parameters on the Shape and Size of TiO_2 Nanoparticles

Francesco Pellegrino,[ab] Raluca Isopescu,[c] Letizia Pellutiè,[a] Fabrizio Sordello,[a] Andrea Mario Rossi,[d] Erik Ortel,[e] Gianmario Martra,[ab] Vasile-Dan Hodoroaba,*[e] Valter Maurino[ab]*

a) *Chemistry Department, University of Torino, Via P. Giuria 7, Torino, 10125, Italy.*
b) *UniTO-ITT Joint Lab, via G. Quarello, 15/A - 10135 Torino, Italy*
c) *University Politehnica of Bucharest, 1-7 G. Polizu Street, Bucharest, 011061, Romania.*
d) *Istituto Nazionale di Ricerca Metrologica, Department of Quality of Life, Strada delle Cacce 91, 10135 Torino, Italy*
e) *Federal Institute for Materials Research and Testing (BAM), Division 6.1 Surface Analysis and Interfacial Chemistry, DE-12200, Berlin.*

*Corresponding authors: francesco.pellegrino@unito.it;
dan.hodoroaba@bam.de;

SUPPORTING INFORMATION

1. **Synthesis**
2. **Characterization of the Ti(IV)-TeoaH$_3$ species**
3. **Reactor's description;**
4. **Post-Synthesis Treatment to obtain Nanopowders**
5. **Experimental Design;**
6. **Nanoparticle Shape and Size Analysis with Electron Microscopy;**
7. **X-Ray Diffraction Pattern analysis;**
8. **Dynamic Light Scattering analysis;**
9. **Second Degree Polynomial Model**
10. **Artificial Neural Networks – ANN Modelling**

1. Synthesis

Materials and Reagents. The precursor used for the hydrothermal synthesis of TiO_2 nanoparticles is a complex of Ti(IV) with $TeoaH_3$ with molar ratio 1:2 as also mentioned in a previous work[1]. The synthesis of the precursor is carried out as follows: in a three-necked (ground joints) round-bottomed flask a certain amount of $TeoaH_3$ (Aldrich Reagent grade 98%) is poured. One of the ground joint is equipped with a dropping funnel loaded with the required quantity of Ti(IV) isopropoxide (Aldrich reagent grade 98%). The flask and the funnels are maintained under a N_2 flow. The Ti(IV) isopropoxide is then dropped into the triethanolamine under vigorous stirring. A vacuum pump is attached to the flask, maintaining a gentle flow of N_2, until the isopropyl alcohol is distilled off. The synthesis product is a pale yellow viscous liquid, to be stored at 4°C under N_2 gas. The reaction scheme is displayed in Figure S1.

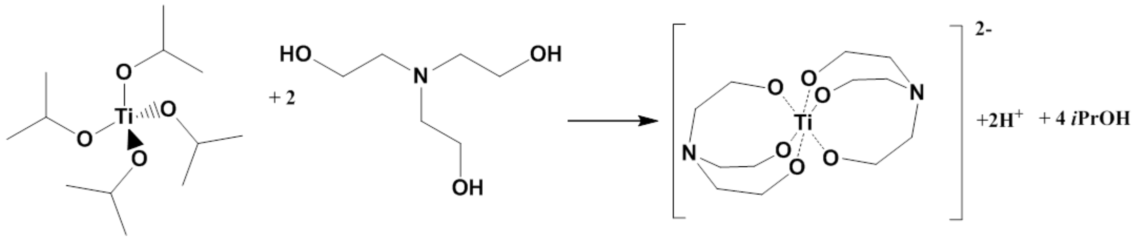

Figure S1. Synthesis of the Ti(IV) – $TeoaH_3$ 1:2 complex

The nominal Ti(IV) content of pure[$Ti(TeoaH)_2$] is 14.00%. However, not all the isopropanol can be eliminated by the vacuum treatment, so the product must be titrated to determine the Ti content[2]. For the complete characterization of the precursor see Supporting Information. The pH adjustment before the synthesis is performed with HCl (Aldrich reagent grade 37%) or carbonate-free NaOH (Sigma Aldrich reagent grade 98%). The syntheses are carried out in a 200 mL Teflon lined stainless steel high pressure reactor mod DAB 3 (Berghof, Tuebingen, Germany). In Supporting Information (Table S1) all the relevant conditions and dimensions of the high pressure reactor are reported.

Hydrothermal Synthesis. The exact conditions for the preparation of [$Ti(TeoaH)_2$] solutions for each type of NPs are reported in Figure S2. In general, after dissolution of the required mass of [$Ti(TeoaH)_2$] and (possibly) of the shape controller ($TeoaH_3$) in ultrapure water (resistivity > 18.2 MΩ cm, TOC < 5 ppb, produced with a Millipore MilliQ apparatus), pH is adjusted with 1 M carbonate-free NaOH or 1 M HCl, as required. The solution is then filtered through a 0.45 μm cellulose acetate membrane filter. The presence of iron in the [$Ti(TeoaH)_2$] solution before hydrothermal treatment is checked by the thiocyanate test (Fe(III) < 0.2 mg L^{-1}). The solution is N_2 purged for at least 10 minutes in order to eliminate O_2 before sealing the hydrothermal reactor. The purging time should be adjusted in order to ensure an O_2 content of the gaseous phase in the reactor < 1% mol/mol. The reactor is heated to 40 °C for 30 minutes, then to the set temperature for the treatment (±1°C) at 1 °C min^{-1}. The temperature is kept

constant for 50 hours. The reactor is then cooled in air. For the post-synthesis treatment see Supporting Information (Paragraph 3).

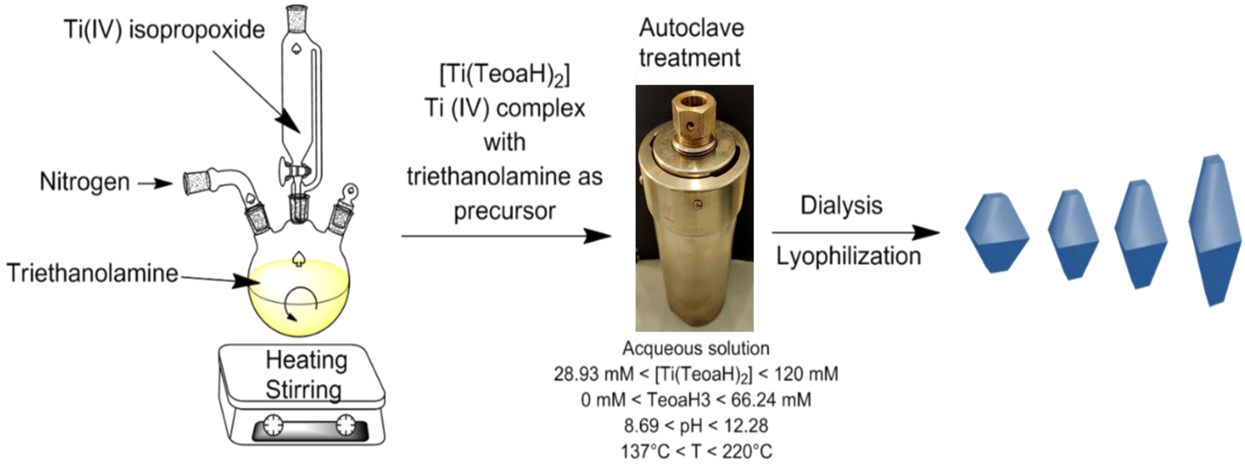

Figure S2. Synthesis scheme of the nanoparticles from the precursor synthesis to the powders.

Nanoparticles Characterization and Image Analysis. Dimensional and shape characterization was performed with two different techniques: Dynamic Light Scattering (DLS) and Scanning Electron Microscopy (SEM).

The DLS system is an ALV (Langen Germany), NIBS model (not invasive backscattering) with fixed angle (173°), He-Ne laser. The hydrodynamic radius R_H of nanoparticles in aqueous suspension was obtained from the Stokes-Einstein equation. DLS data were obtained by analysing the raw suspensions (if necessary diluting them using 200 mM NH_3 as a dispersant) after sonication for 10-30 minutes in an ultrasound bath and in a closed vial to avoid NH_3 evaporation (suggested 95 W, 37 kHz). The reported sizes were obtained by fitting the decay time distribution function to the integral equation relating the field correlation function and the defined distribution function using a constrained regularization method (CONTIN DP algorithm) developed by Provencher.[3] The mass and number distribution functions were then obtained.

In this study, SEM was performed with a Zeiss Supra 40 instrument (Zeiss, Oberkochen, Germany) equipped with a Schottky field emitter and, additionally to the standard Everhart-Thornley detector, with a high-resolution secondary electron InLens detector. Hence, high-resolution SEM imaging of the sample surface morphology at the nanometre scale is possible. This type of microscopy is especially suited to get access to the size and shape of individual NPs. Transmission electron microscopy (TEM) - either as the conventional TEM or as the transmission mode at an SEM, i.e. T-SEM [4] – was successfully applied for the analysis of well-dispersed, isolated NPs deposited on a conventional TEM grid.[4-5] The T-SEM mode (also named STEM-in-SEM) has been applied by using a dedicated sample holder allowing the primary electron beam to only transmit through the electron-transparent sample (NPs prepared on typical TEM grid) and being finally analysed by the conventional secondary electron (Everhart-Thornley) detector.[14b] The particular advantage of using the T-SEM is the superior accuracy in the identification of the NP boundaries, and hence, measuring the NP (projection) size with good accuracy.

Aspect ratio. The aspect ratio can be defined as the ratio between the size along the c-axis and the size of the bipyramid base (a-axis). Ideally, in the case of a perfect bipyramid, the shape of the particles may be obtained by processing a three-dimensional image, adjusting the image of the particle to a truncated bipyramidal contour. For a perfect bipyramidal crystal, having the anatase lattice parameters, this ratio is equal 2.51. This procedure, however, is suitable only on three-dimensional images of excellent quality and requires ad hoc software. The image processing (carried out with the software ImageJ)[6] was carried out on two-dimensional images, by calculating the lengths of the major (*Max*) and minor (*Min*) axes of the elliptical perimeter fitted to the contours of the 2-D projection of the nanoparticles.
The parameters *Max* and *Min*, however, can be measured accurately only on particles whose c-axis is parallel or perpendicular to the TEM grid. Thus, this scenario is not optimal for arbitrarily oriented NPs, and leads to an underestimation of the parameters *Max* and *Min*; the expected aspect ratio for a perfect truncated bipyramid (given the parameters of the anatase crystal) is therefore 1.5. In order to compare the results of size measurements by DLS with T-SEM, the geometrical data determined by T-SEM analysis were converted to a hydrodynamic radius (assuming the NPs as prolate ellipsoids) with the Perrin formula:[7]

$$\frac{R_H}{R_S} = \frac{\sqrt{p^2-1}}{p^{1/3}\ln\left(p+\sqrt{p^2-1}\right)}$$

$$p = Max/Min > 1\, prolate$$

where:

- R_H hydrodynamic radius of the ellipsoid,
- R_S radius of the sphere with the same volume

In this way, a direct comparison between DLS measurements (for which the measurand is the hydrodynamic radius) and T-SEM images (which give geometric size parameters) becomes possible.

2. Characterization of the Ti(IV)-TeoaH$_3$ species

A freshly prepared 40 mM Ti(TeoaH)$_2$ solution, diluted 1:100 with methanol, was analyzed by ESI-HRMS in order to characterize the Ti(IV) solution species. The spectrum is reported in Figure S3. The signal at m/z 172.0848 is the TeoaH$_3$ - Na$^+$ adduct ($C_6H_{15}O_3NNa$, Δmmu = -9.575). It is present also the signal of protonated TeoaH$_3$ at m/z 150.1040 ($C_6H_{16}O_3N$, Δmmu = -8.48). The presence of free TeoaH$_3$ signals means that the Ti(TeoaH)$_2$ complex is partially decomposed. The clusters centred at m/z 343.1126, 365.0963, 381.0693 have all the isotopic pattern of a molecule containing an atom of Ti (with the M+1 isotopic peak higher than expected due to the ^{13}C contribution,Figure S4). The first signal corresponds to the empirical formula $C_{12}H_{27}O_6N_2Ti$, the second to $C_{12}H_{26}O_6N_2TiNa$, the third to $C_{12}H_{26}O_6N_2TiK$, so they are the Ti(TeoaH)$_2$ complex cationized with H^+, Na^+ and K^+ respectively.
The clusters centred at m/z 536.1268, 558.1074, 574.0802 have all an isotopic pattern of a molecule containing two Ti atoms (Figure S5). The m/z ratios correspond to the empirical formulae $C_{18}H_{38}O_9N_3Ti_2$, $C_{18}H_{37}O_9N_3Ti_2Na$ and $C_{18}H_{37}O_9N_3Ti_2K$, so they are the complex Ti$_2$H(Teoa)$_3$ cationized with H^+, Na^+ and K^+ respectively. It is worth to note that the mass difference between Ti(TeoaH)$_2$ and Ti$_2$H(Teoa)$_3$ is 193.0112, that, obviously, correspond to the mass of Ti plus Teoa (not TeoaH$_3$) minus the mass of a hydrogen. The series is completed by the clusters centered at 729.1384, 751.1193 and 767.2993, which correspond to Ti$_3$(Teoa)$_4$

($C_{24}H_{48}O_{12}N_4Ti_3$) cationized by H^+, Na^+ and K^+ respectively. It is not possible to continue the sequence because $Ti_3(Teoa)_4$ has no longer mobile hydrogens. So the cluster centred at 938.3310 cannot be assigned to protonated $Ti_4(Teoa)_5$. The mass difference between this ion and protonated $Ti_3(Teoa)_4$ at m/z 729.1384 is no longer 193.0112, but 209.1926. This corresponds to Ti plus Teoa plus oxygen minus hydrogen. So with this multinuclear complex the hydrolysis of Ti(IV) is starting. This is in partial agreement, at least with the stoichiometry, with the reported structures of titanatranes crystallized from organic solvents[8].

In conclusion, the solutions of $Ti(TeoaH)_2$ are not thermodynamically stable already at ambient temperature. However, the hydrolysis in these conditions is slow and it could take days to go to completion. Taking into account that apparent activation energies of Ti-alkoxides hydrolysis are of the order of 30 kJ mol^{-1} [9], an increase of temperature of 100 K should increase the hydrolysis rates of three orders of magnitude in basic conditions, so the reaction goes to completion within minutes.

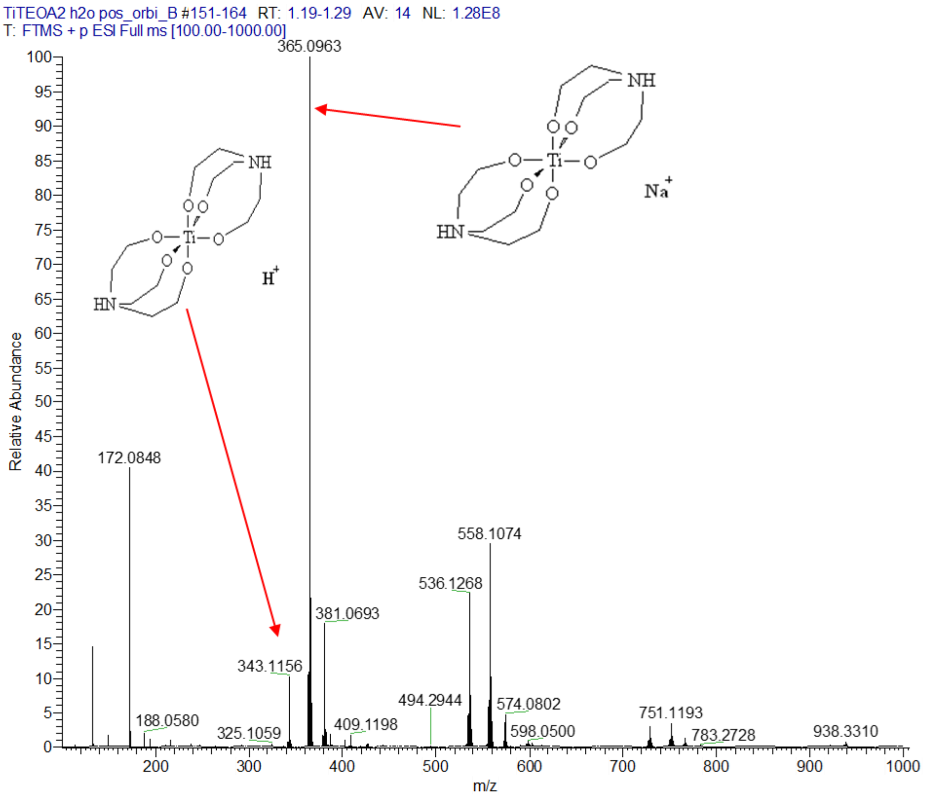

Figure S3. ESI-HRMS spectrum of a 40 mM aqueous solution of $Ti(TeoaH)_2$ diluted 1:100 in methanol.

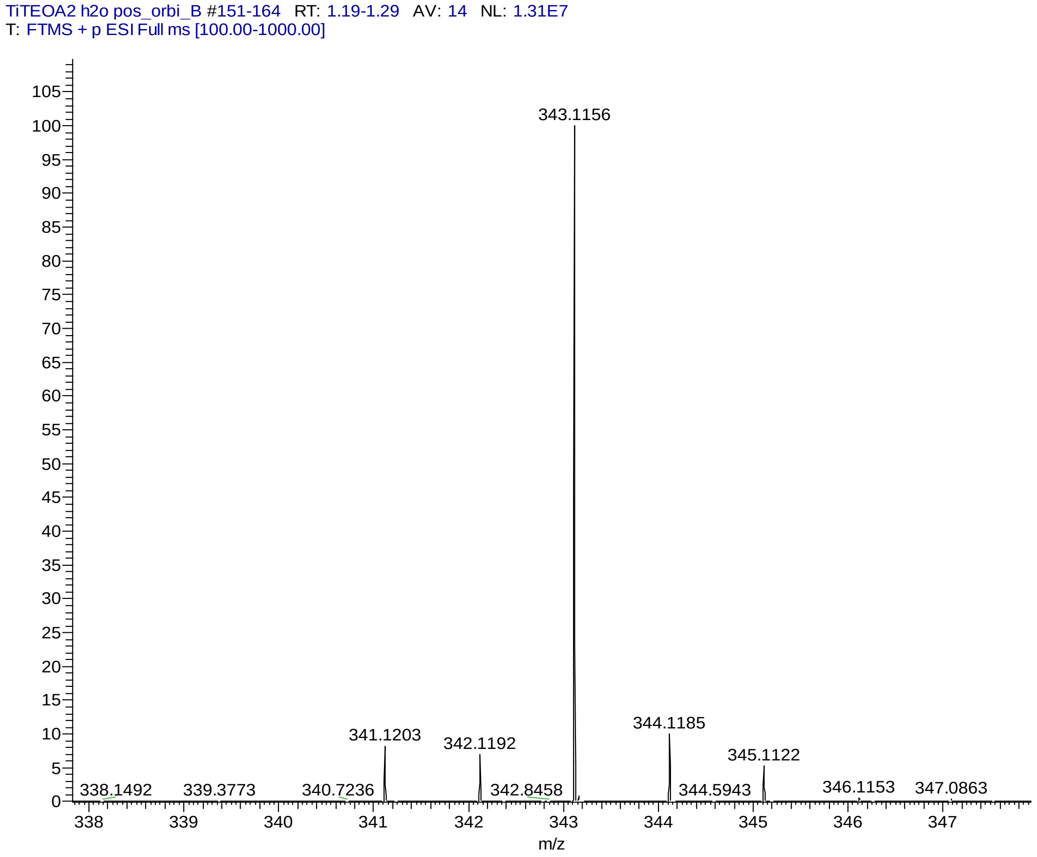

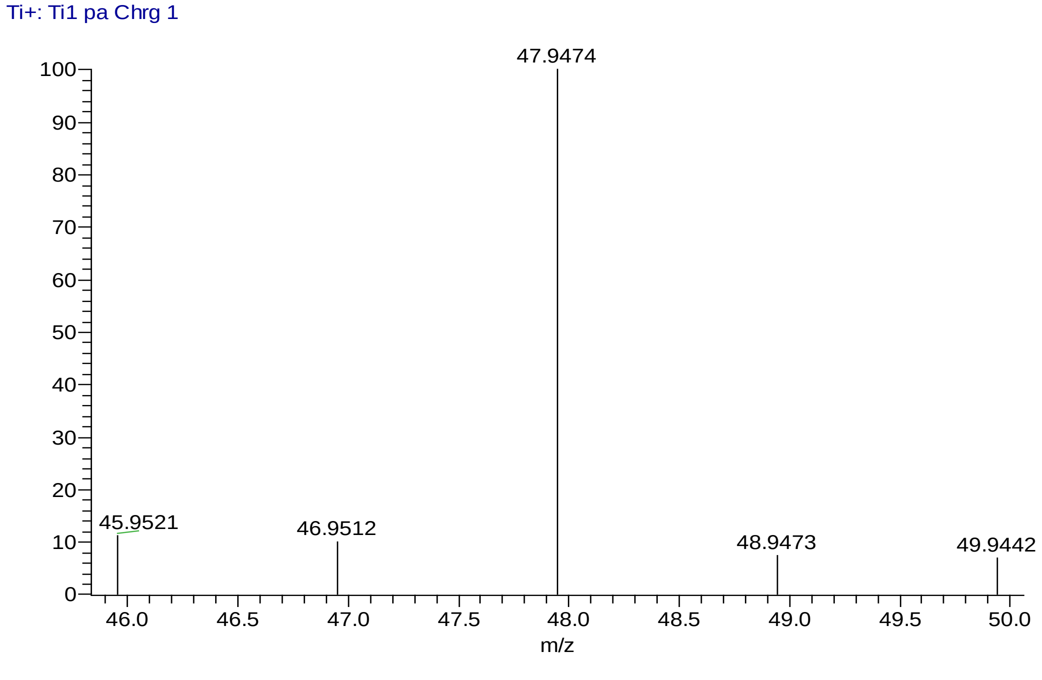

Figure S4. Isotopic pattern of the peak at m/z 226.0535 ($C_7H_{16}O_4NTi$), at 343.1313 ($C_{12}H_{26}O_6N_2Ti$, protonated) in the ESI-HRMS spectrum of the methanolic $Ti(TeoaH)_2$ solution (top) and the isotopic pattern of Ti (below).

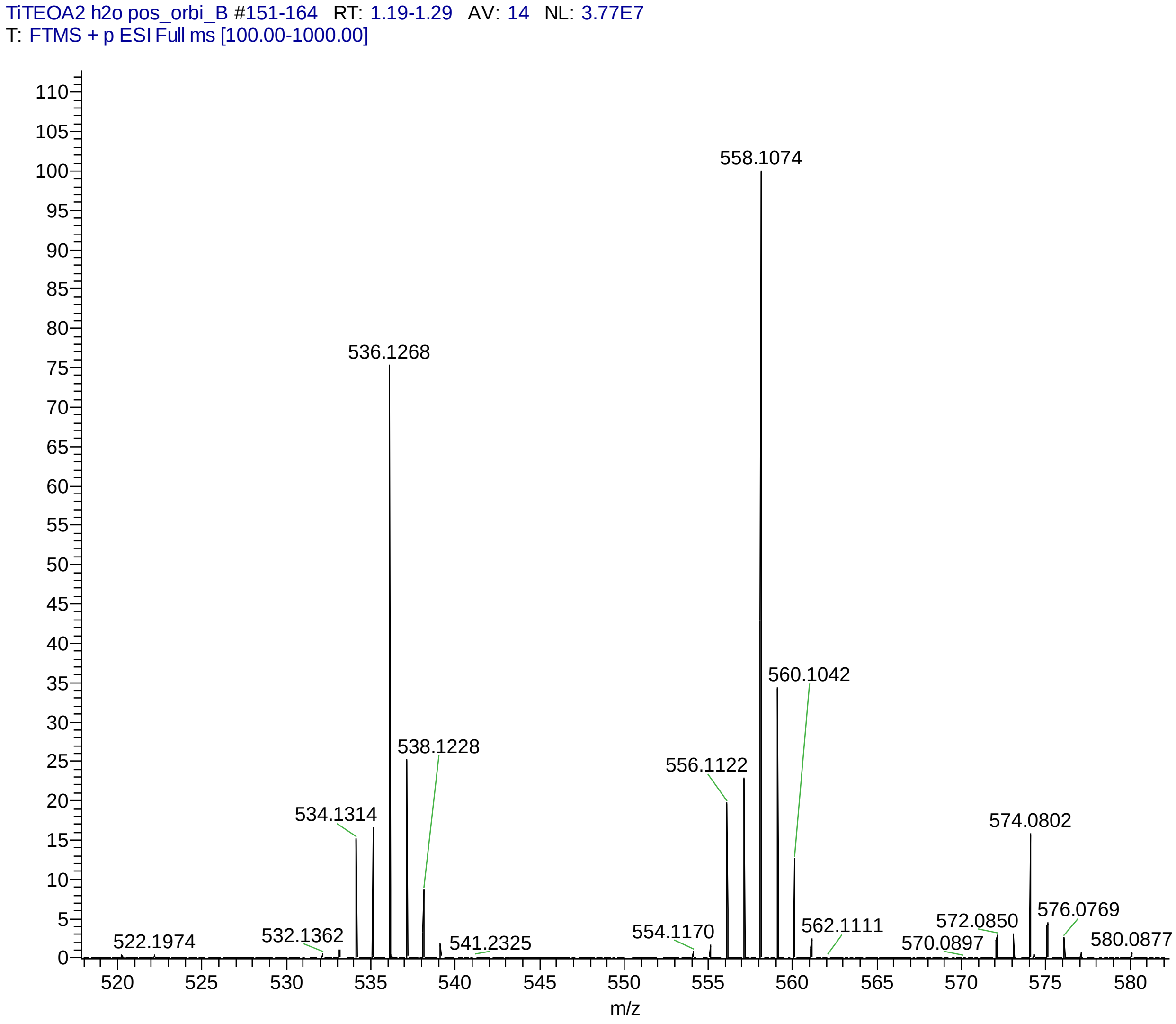

Figure S5. Isotopic patterns of the $Ti_2H(Teoa)_3$ complex cationized with H^+, Na^+ and K^+.

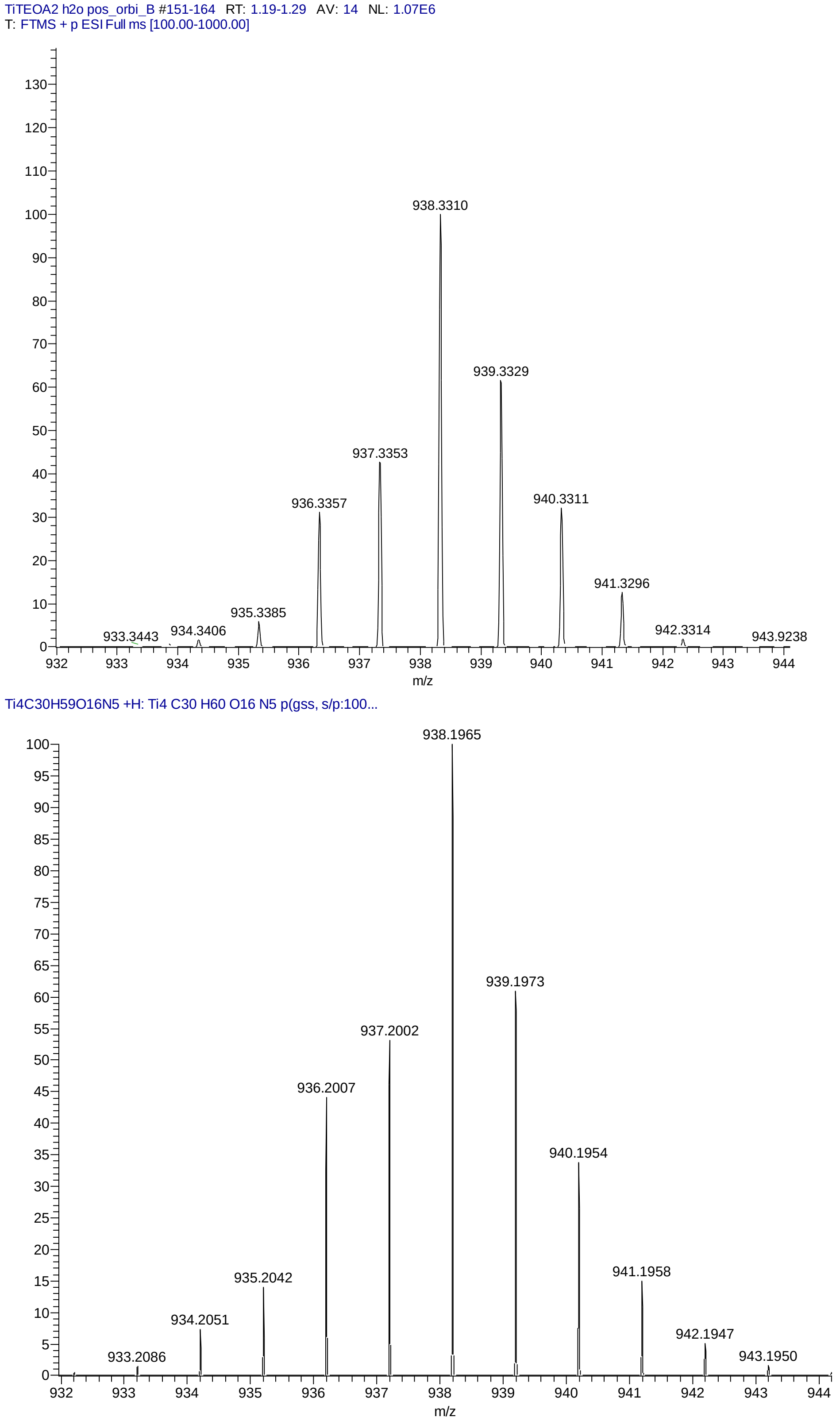

Figure S6. Isotopic cluster centered at m/z 938.3310 (top) and the simulation of the isotopic cluster of an ion of formula $Ti_4C_{30}H_{59}O_{16}N_5$ + H^+ ($Ti_4O(Teoa)_5$ – H) + H^+ (below).

3. Reactor's description

The synthesis was carried out in a 200 mL Teflon lined stainless steel high pressure reactor mod DAB3 (Berghof, Tuebingen, Germany) (Figure S7). The heating/stirring was carried out with a heating mantle mounted on a magnetic stirrer/heater (Heidolph, Germany, mod MR-HEI standard) and a temperature controller/programmer mod BTC-3000 (Berghof, Germany) equipped with a type K thermocouple (see Figure S7 for the complete assembly). In Table S1 all the relevant conditions and dimensions of the high pressure reactors are reported.

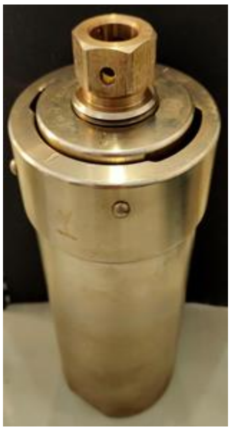

Figure S7. Berghof DAB3 Teflon lined stainless steel high pressure reactor.

High pressure reactor Description	
internal height (mm)	100.5
internal diameter (mm)	51.5
volume of liquid (ml ± 10 ml)	150
Magnetic stir bar	
length (mm)	30
diameter (mm)	6
stirring speed (rpm)	750

Table S1. Characteristics of the high pressure reactor and of the magnetic stirrer.

4. Post-Synthesis Treatment to obtain Nanopowders

At the end of the hydrothermal treatment the nanoparticles suspension is concentrated using a rotovapor system and then processed according to the following procedure:

1. Dialysis of the concentrated raw suspension against ultrapure water (MilliQ, Millipore) using a Spectra/Por dialysis membrane tubing (MWCO 8-12 kD or MWCO 12-14 kD), final pH of the permeate in the range 5-8, Cl^- and SO_4^{2-} < 1 mg L^{-1} (by ion chromatography).
2. Freeze-drying of the suspension.
3. Resuspension in milli-Q water (ca. 100 g/L)
4. Addition of 5 g L^{-1} of H_2O_2 and irradiation (in air) for 24 hours of the suspension, after addition of 50 g/L H_2O_2, under UV light using a Medium Pressure Mercury Lamp (emission wavelength 360 nm) or a fluorescent blacklit lamp (360 nm), ca. 20 W/m^2 in the range 300-400 nm.
5. Dialysis against milli-Q water using a Spectra/Por dialysis membrane tubing (MWCO 8-12 kD or MWCO 12-14 kD), final pH of the permeate in the range 5-8, Cl^- and SO_4^{2-} < 1 mg L^{-1} (by ion chromatography).
6. Freeze-drying of the suspension.

The choice to carry out dialysis plus freeze-drying in order to wash the NPs and eliminate the solvent was made taking into account that:

1. dialysis and freeze-drying allow recovering all the particles without changing the size distribution. Centrifugation or filtration can selectively lose particles on the fine side of the size distribution.
2. Freeze-drying allows the elimination of water without any thermal treatment, making no variation in the surface properties of the NPs.

5. Experimental Design

Starting from a previously developed synthetic procedure for obtaining TiO_2 bipyramidal nanoparticles, the synthesis conditions were modified by means of an experimental design. The experimental design technique used is a Box Wilson central composite designs (CCD). According to the design of experiments theory, a central composite design is a factorial design with center points, with a group of axial points, called "star points", that allow the estimation of the response surface curvature. If n is the number of factors, the 2n corner points have the normalized coordinates +/- 1 while the star points are created by drawing a line from the center orthogonal on each face. The star points represent new extreme values (low and high) for each factor in the design. In our case the chosen factors are 4 (n=4) as described below, so the number of the "star points" is 2n = 8. The distance α from the center of the eight "star points", for an orthogonal experiment, in normalized coordinates, is calculated from the relation[10]:

$$\alpha^4 + 2^{n-1}\alpha^2 - 2^{n-2}(n + 0{,}5N_0) = 0$$

where n is the number of factors (n=4), and N_0 the number of center points (N_0 = 4).

The experimental plan considered the four independent variables (factors) that mainly influence the product characteristics:

- Z_1 - Ti $(TeoaH)_2$ initial concentration;
- Z_2 - Added $TeoaH_3$ concentration as shape controller;
- Z_3 - Initial pH;
- Z_4 – operating temperature.

The output variables are hydrodynamic radius (Y_1), polydispersity index (Y_2) and the aspect ratio (Y_3, see below for its definition and measurement). The variables X_1, X_2, X_3, X_4 are the coded factors corresponding to the natural variables Z_k (k = 1, 2, 3, 4). The coded factors are obtained using the general relations:

$$z_k^0 = 0{,}5(z_k^{\max} + z_k^{\min}), \quad \Delta z_k = \frac{z_k^{\max} - z_k^0}{\alpha}, \quad x_k = \frac{z_k - z_k^0}{\Delta z_k} \qquad (\text{ k = 1,2,3,4})$$

where z_k^0 are the central point coordinates:

$z_1^0 = 75mM$; $z_2^0 = 40mM$; $z_3^0 = 10\,pH$; $z_4^0 = 170^0 C$,

Δz_k represent the distances from the central point to the factorial levels:

$\Delta z_1 = 36mM$; $\Delta z_2 = 17mM$; $\Delta z_3 = 1.3pH$; $\Delta z_4 = 32.8^0 C$

and x_k represents the values of the work matrix of the experimental program expressed in normalized coordinates.

The experimental ranges studied were from 29 to 120 mM [$Ti(TeoaH)_2$], from 0 to 66 mM added $TeoaH_3$, pH between 8.7 and 12 and temperature values from 137 to 220°C. Taking into account the great complexity of the experimental work involved, a fractional central composite design was selected with 8 factorial points, 8 star points and 4 central points. For the selected experiment plan α=1.525. The orthogonal fractional centered composite design, expressed in real factor values, is shown in Table 1.

Table S2. Work matrix for the experimental program, based on orthogonal fractional design Box Wilson. In the table we also reported the crystal phase (see the relevant paragraph) determined by XRD.

Experiment	Z_1, mM	Z_2, mM	Z_3, pH	Final pH	Z_4, °C	[$TeoaH_3$]/[Ti]	Crystal Phase
HT01	29	14	8.7	8.8	137	2.5	anatase
HT02	29	14	11.3	11.6	203	2.5	anatase
HT03	29	66	8.7	/	203	4.2	anatase
HT04	29	66	11.3	11.6	137	4.3	anatase & brookite (~5%)
HT05	101	14	8.7	8.9	203	2.1	anatase
HT06	101	14	11.3	12.1	137	2.1	anatase & brookite (~7%)
HT07	101	66	8.7	8.8	137	2.7	anatase
HT08	101	66	11.3	12.2	203	2.7	anatase
HT09	10	40	10	10.1	170	5.9	anatase
HT10	120	40	10	11.6	170	2.3	anatase
HT11	65	0	10	11.2	170	2.0	anatase
HT12	65	80	10	11.5	170	3.3	anatase
HT13	65	40	8	8.2	170	2.6	anatase
HT14	65	40	12	12.3	170	2.6	anatase & brookite (~15%)
HT15	65	40	10	10.1	120	2.6	anatase
HT16	65	40	10	11.0	220	2.6	anatase
HT17	65	40	10	11.4	170	2.6	anatase
HT18	65	40	10	11.3	170	2.6	anatase

**HT19**	**65**	**40**	**10**	**11.4**	**170**	**2.6**	**anatase**
**HT20**	**65**	**40**	**10**	**11.4**	**170**	**2.6**	**anatase**

Table S3. Dimensional parameters obtained by T-SEM micrograph analysis with the corresponding standard deviations and average size of crystal domain of the c-axis (D_{004}) obtained by Scherrer Analysis of the 004 XRD reflex.

Experiment	Minor = "Min", nm	Major = "Max", nm	Max/Min = p	D_{004}, nm
**HT01**	15 ± 3	23 ± 7	1.5 ± 0.5	28
**HT02**	45 ± 10	89 ± 33	2.0 ± 0.9	77
**HT03**	28 ± 5	38 ± 9	1.4 ± 0.4	35
**HT04**	20 ± 4	108 ± 47	5.5 ± 2.6	62
**HT05**	21 ± 4	29 ± 8	1.4 ± 0.5	36
**HT06**	18 ± 4	100 ± 31	5.5 ± 2.1	49
**HT07**	16 ± 4	27 ± 10	1.7 ± 0.8	31
**HT08**	35 ± 6	81 ± 37	2.3 ± 1.1	52
**HT09**	32 ± 4	43 ± 11	1.4 ± 0.4	39
**HT10**	30 ± 5	52 ± 15	1.8 ± 0.6	48
**HT11**	29 ± 4	39 ± 9	1.3 ± 0.4	37
**HT12**	33 ± 4	49 ± 12	1.5 ± 0.4	53
**HT13**	21 ± 4	29 ± 9	1.4 ± 0.5	30
**HT14**	/	/	/	/
**HT15**	/	/	/	/
**HT16**	35 ± 4	49 ± 10	1.4 ± 0.3	44
**HT17**	31 ± 4	47 ± 10	1.5 ± 0.4	47
**HT18**	32 ± 4	45 ± 11	1.4 ± 0.4	-
**HT19**	32 ± 4	43 ± 10	1.4 ± 0.4	-
**HT20**	32 ± 4	44 ± 11	1.4 ± 0.4	-

Table S4. Hydrodynamic radii extracted from the T-SEM images analysis and DLS measurements of the TiO_2 particulate samples.

Experiment	R_H Perrin (TSEM), nm	R_H ± SD Mode 1 (DLS), nm	R_H Mode 2 (DLS), nm
**HT01**	9	7.0 ± 0.3	24
**HT02**	29	20 ± 1	58
**HT03**	16	15 ± 1	35
**HT04**	22	22 ± 2	68
**HT05**	12	14 ± 1	35
**HT06**	20	20 ± 1	44
**HT07**	10	18 ± 4	/
**HT08**	25	17 ± 1	126
**HT09**	18	20 ± 4	/
**HT10**	19	19 ± 4	/
**HT11**	16	17 ± 3	/
**HT12**	19	21 ± 3	/
**HT13**	12	13± 1	35
**HT14**	/	250 ± 20	/
**HT15**	/	2.0 ± 0.3	12

**HT16**	20	20 ± 4	/
**HT17**	18	19 ± 3	/
**HT18**	18	18 ± 4	/
**HT19**	18	19 ± 3	/
**HT20**	18	19 ± 3	/

6. Nanoparticle Shape and Size Analysis

The shape analysis was carried out by TEM and T-SEM for various NP sets resulted from the experimental design syntheses. At least 500 NPs were analysed for each sample. The T-SEM and TEM imaging was performed on the raw suspensions of the NPs as obtained from the hydrothermal syntheses. Representative T-SEM micrographs of the materials HT06, HT08 and HT16 are displayed in Figure 2. In Table S3 the dimensional parameters obtained by means of T-SEM image analysis are reported, i.e. minor (*Min*) and major (*Max*) axes lengths of the ellipse that fits the contour of the 2D projection of the NP prepared on a TEM grid (more details in the Methods section) and the shape parameters. Due to the substantial presence of brookite in the sample HT14 that make impossible an accurate shape and size analysis of particulate material and the difficulties in the analysis of the material HT15 (complex, not well-defined morphology, Figure S22). In order to avoid a strong model distortion, the missing data for the aspect ratio were complemented with data in the range of the other experimental values, while the outlier values for Y1 and Y2 in HT14 run were replaced with additional values in the range of variation.[11]

.

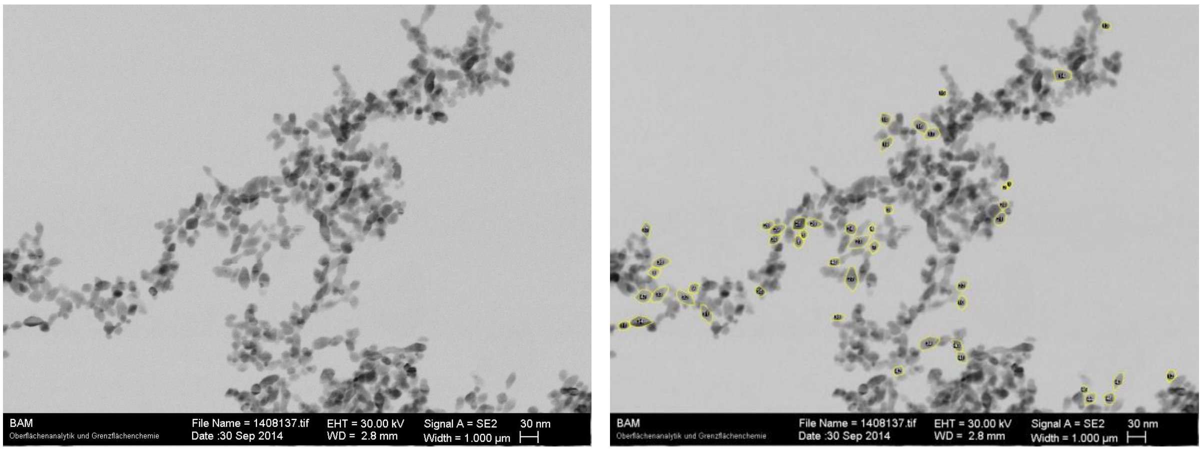

Figure S8. T-SEM images of sample HT01.

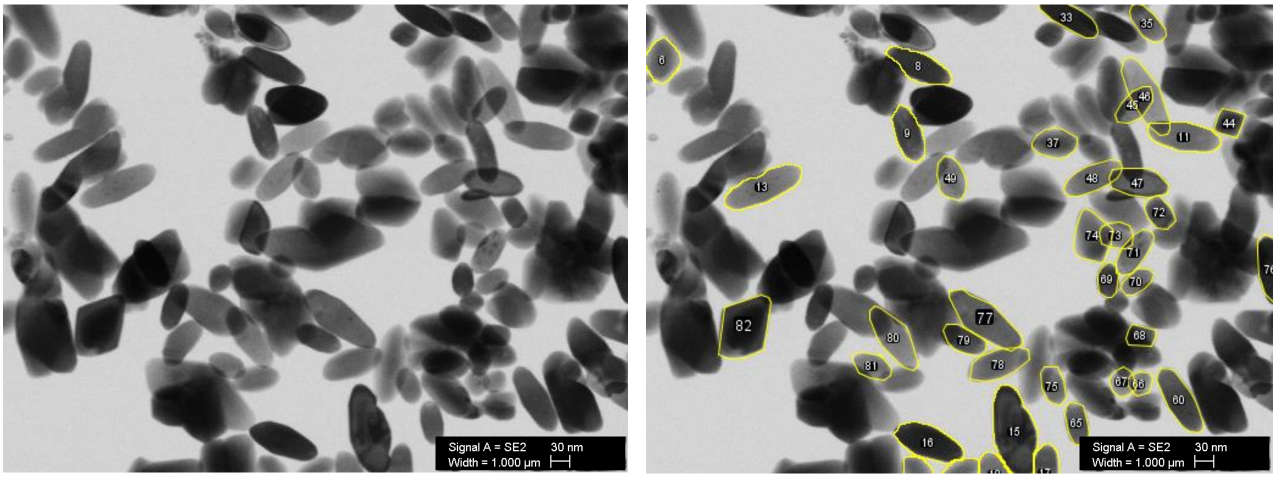

Figure S9. T-SEM images of sample HT02.

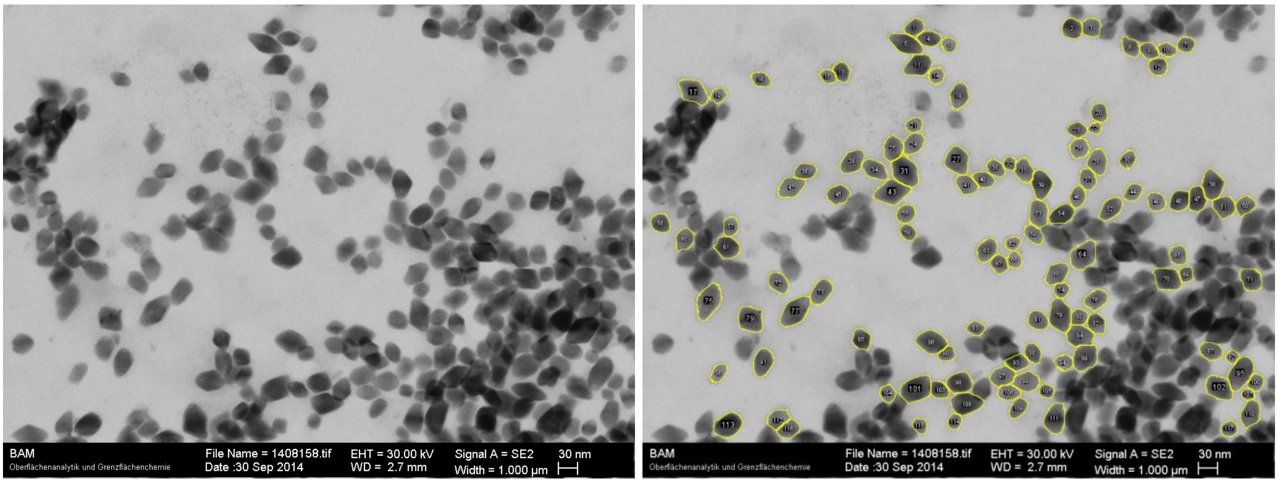

Figure S10. T-SEM images of sample HT03.

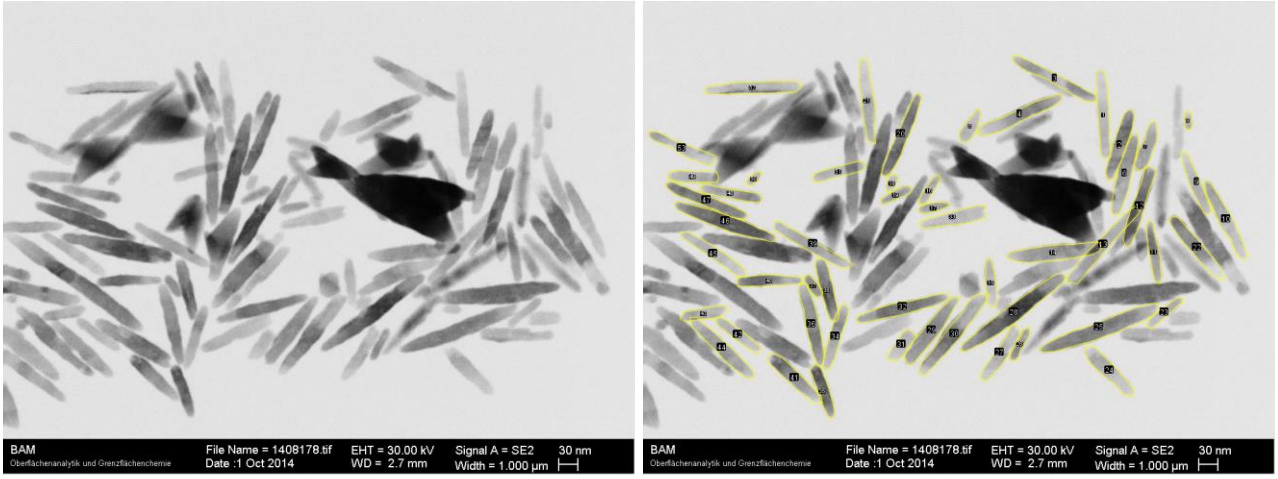

Figure S11. T-SEM images of sample HT04.

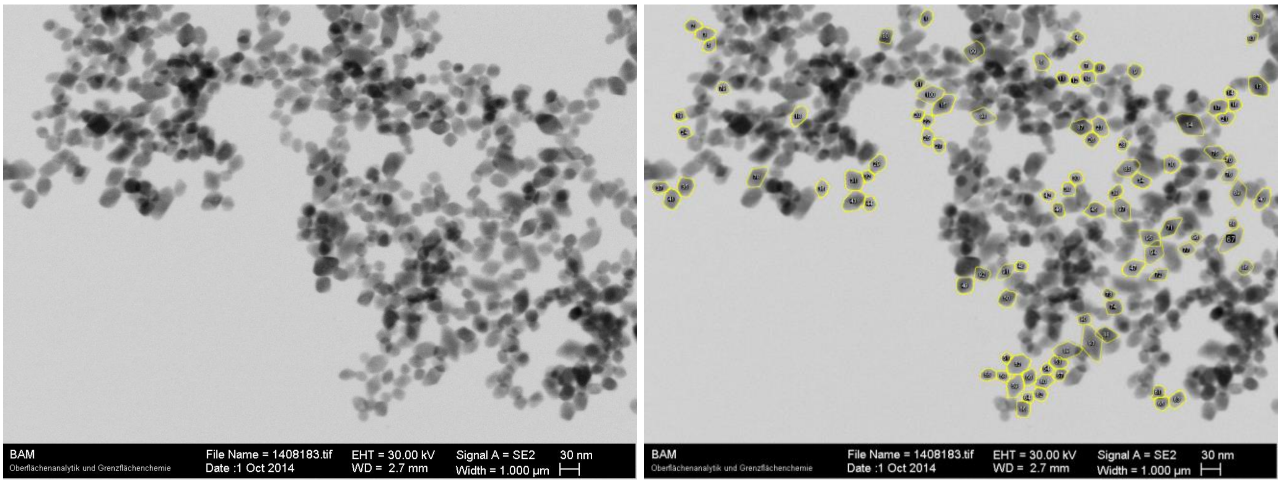

Figure S12. T-SEM images of sample HT05.

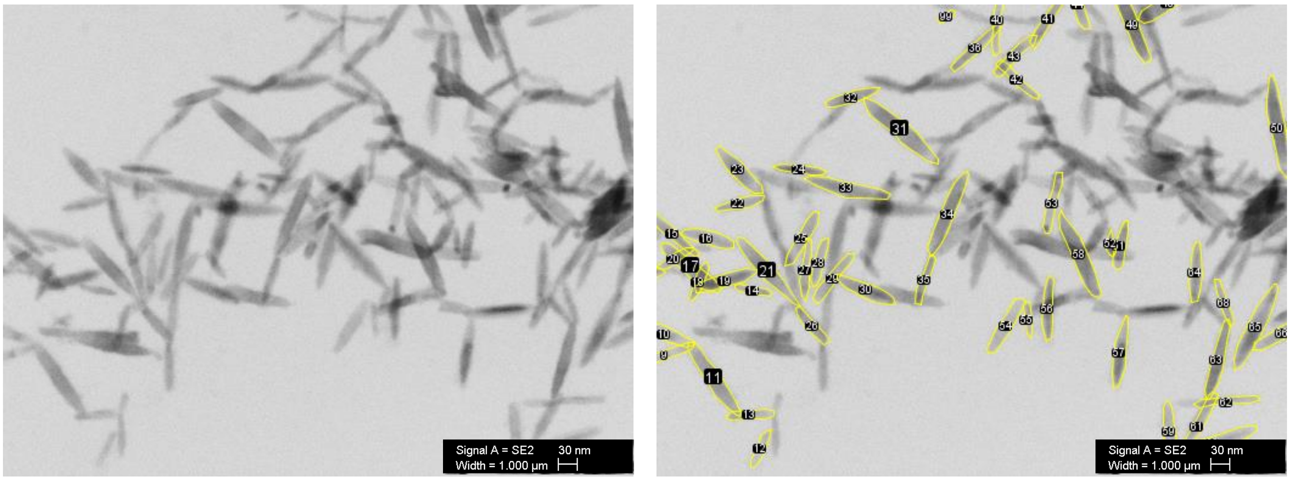

Figure S13. T-SEM images of sample HT06.

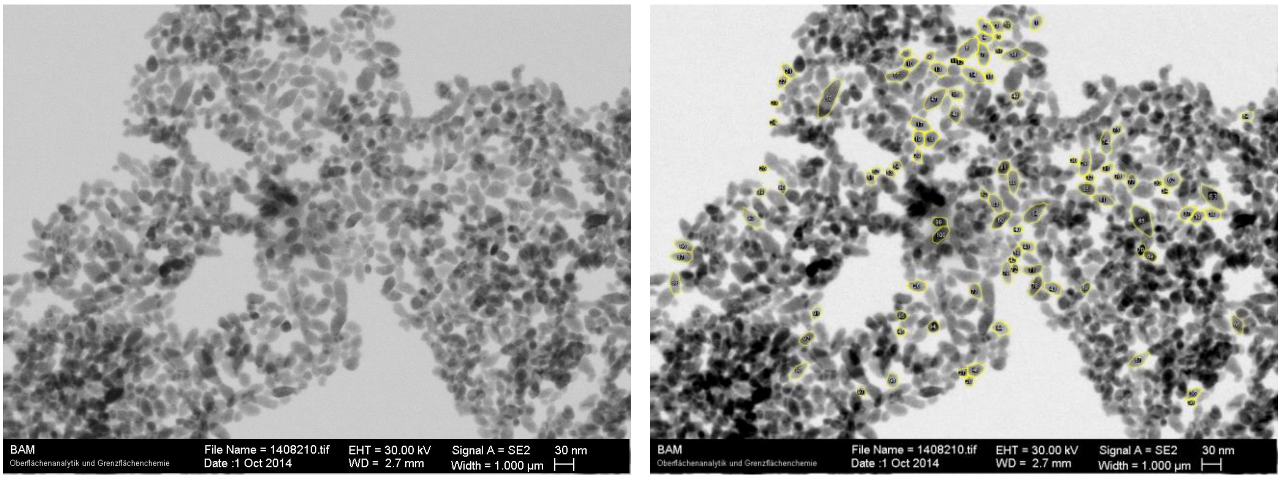

Figure S14. T-SEM images of sample HT07.

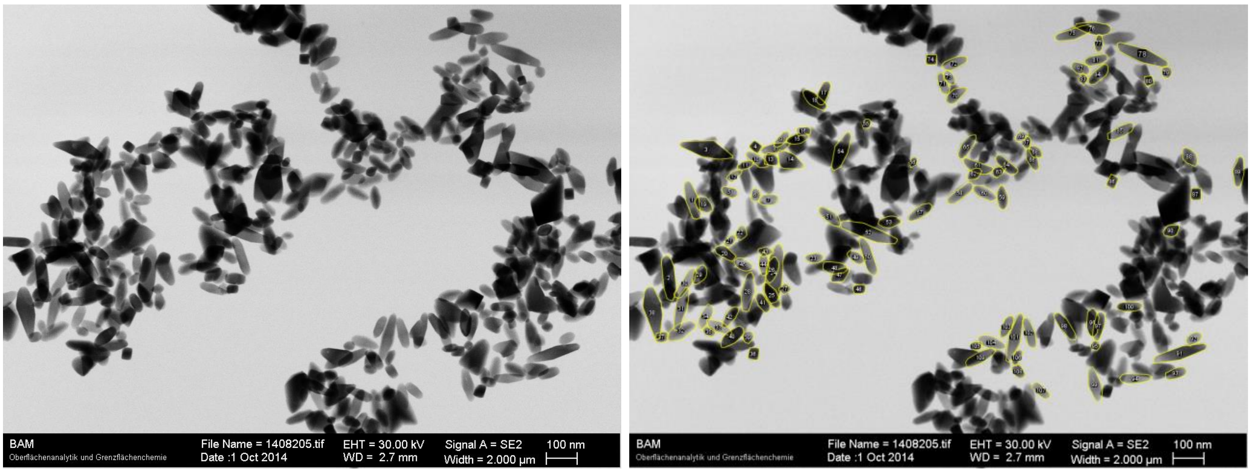

Figure S15. T-SEM images of sample HT08.

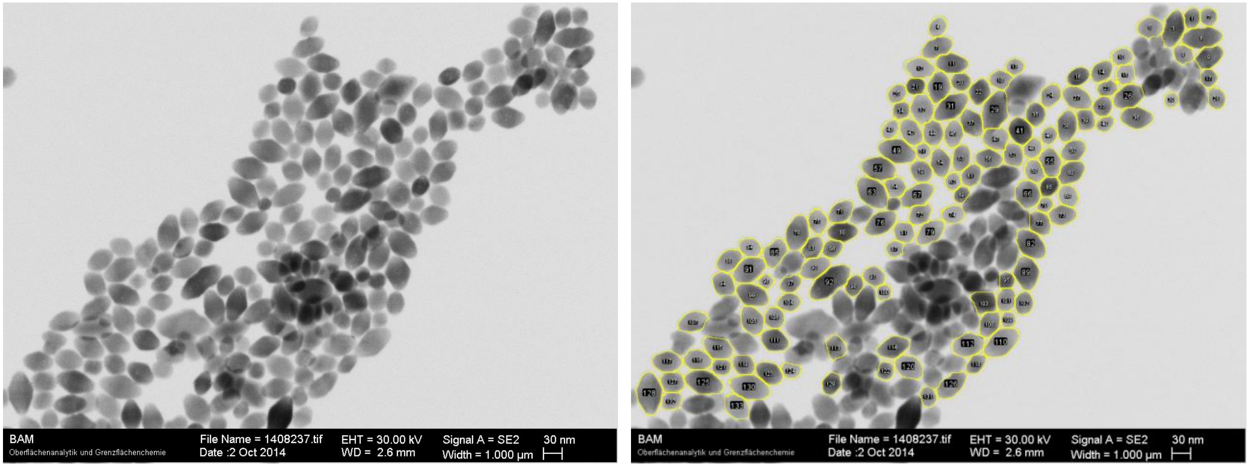

Figure S16. T-SEM images of sample HT09.

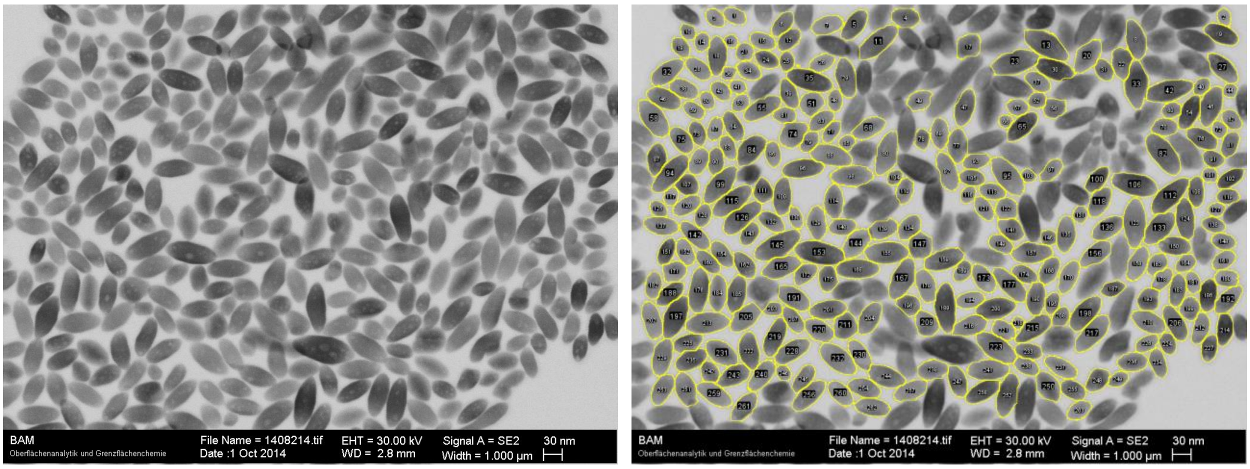

Figure S17. T-SEM images of sample HT10.

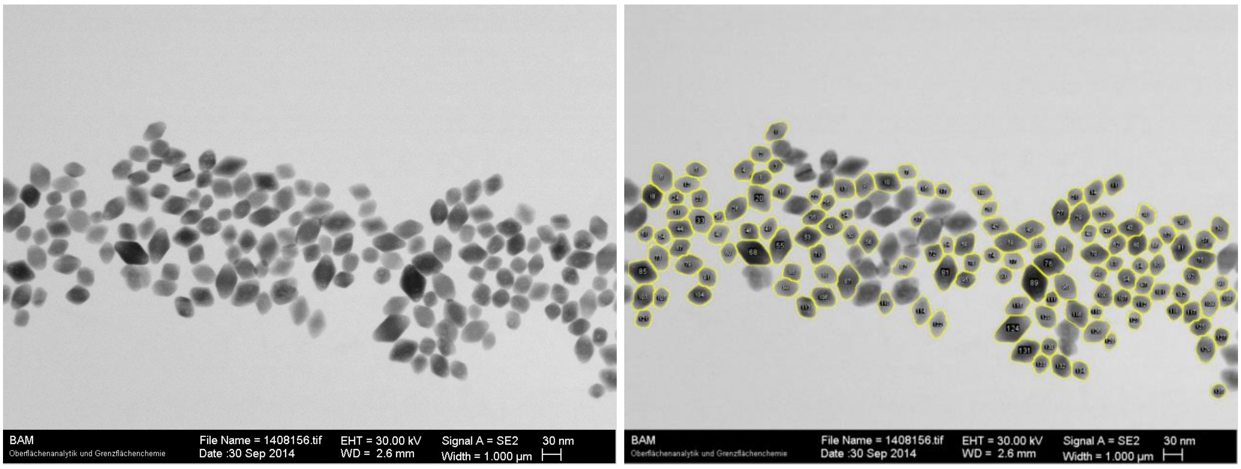

Figure S18. T-SEM images of sample HT11.

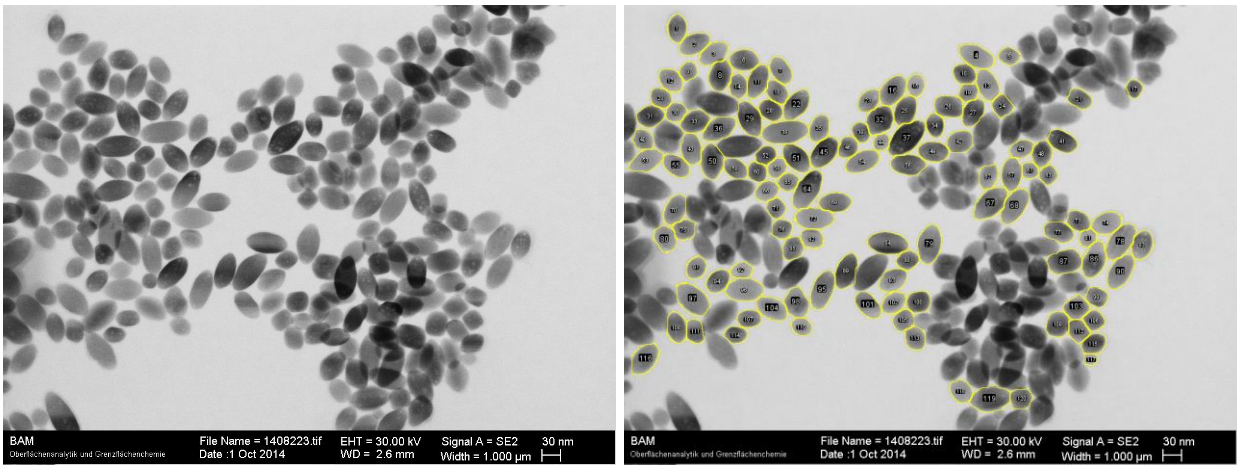

Figure S19. T-SEM images of sample HT12.

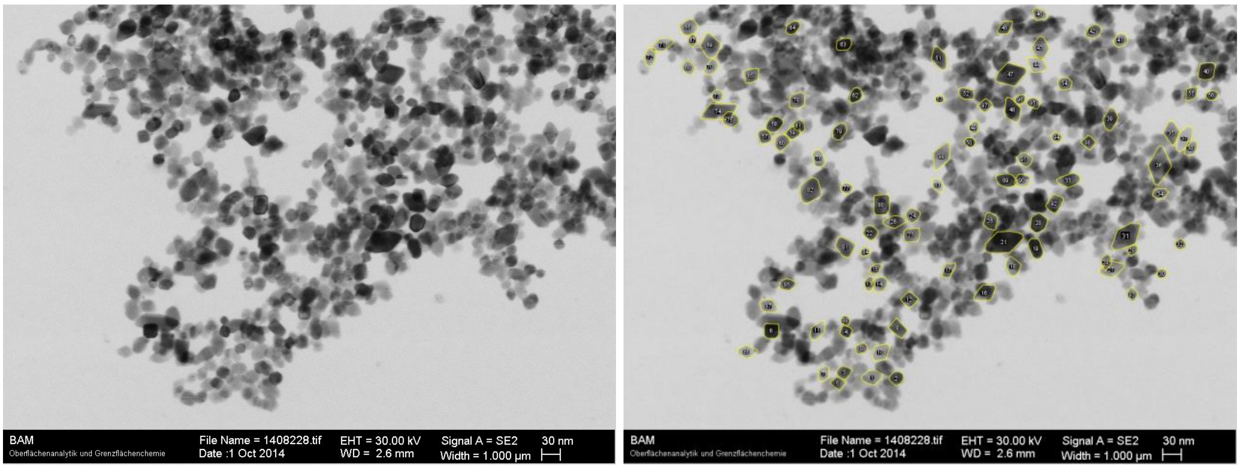

Figure S20. T-SEM images of sample HT13.

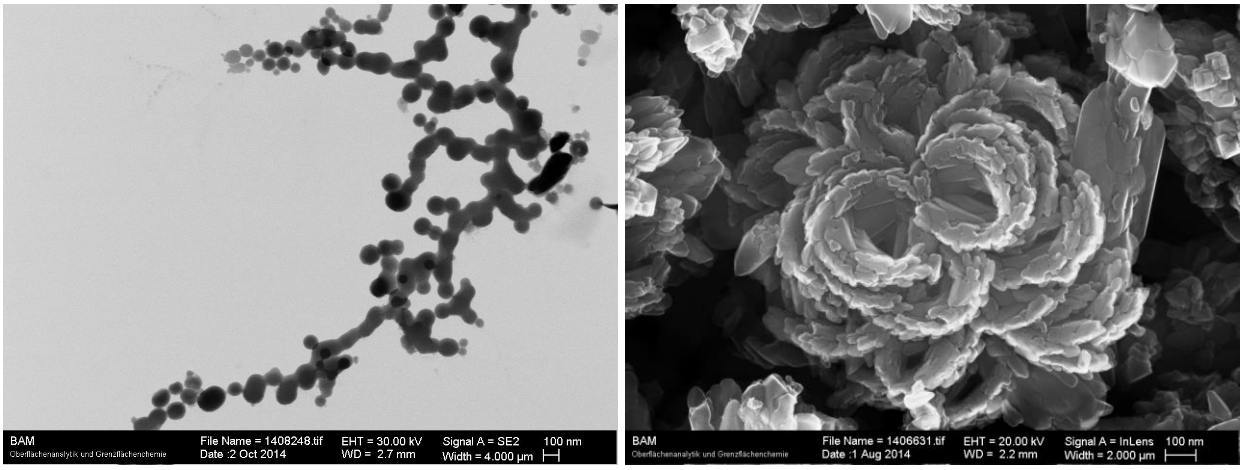

Figure S21. T-SEM images of sample HT14.

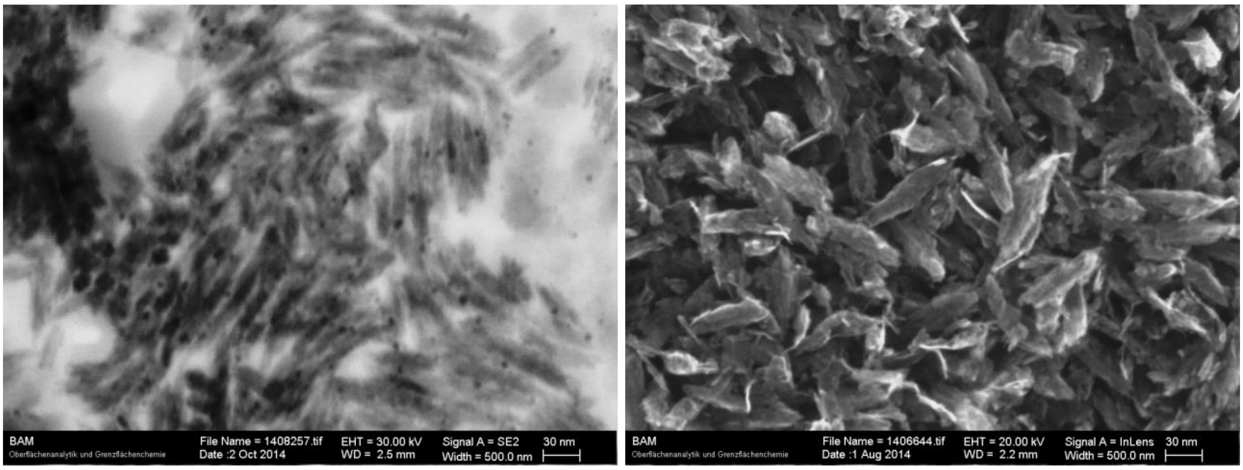

Figure S22. T-SEM images of sample HT15.

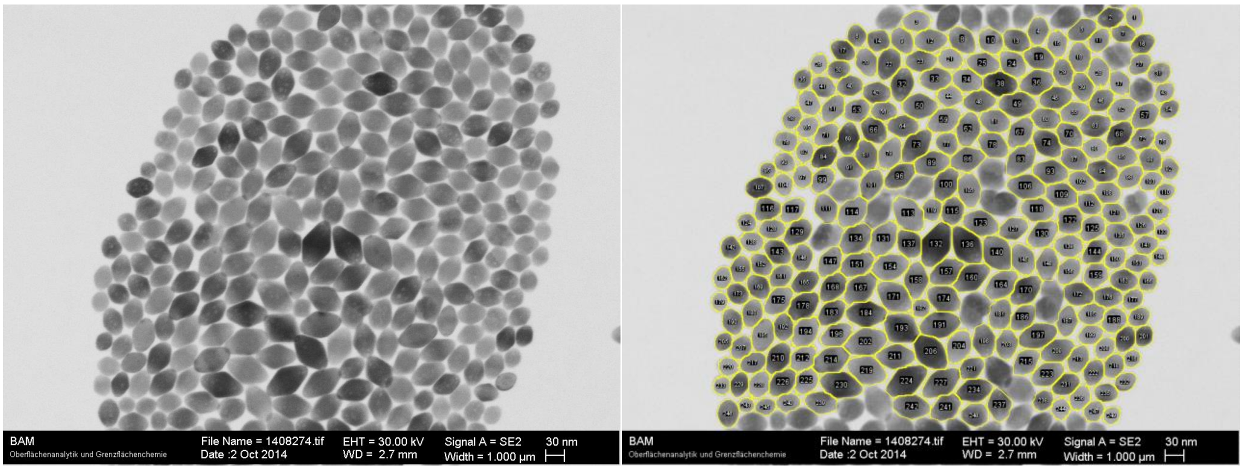

Figure S23. T-SEM images of sample HT16.

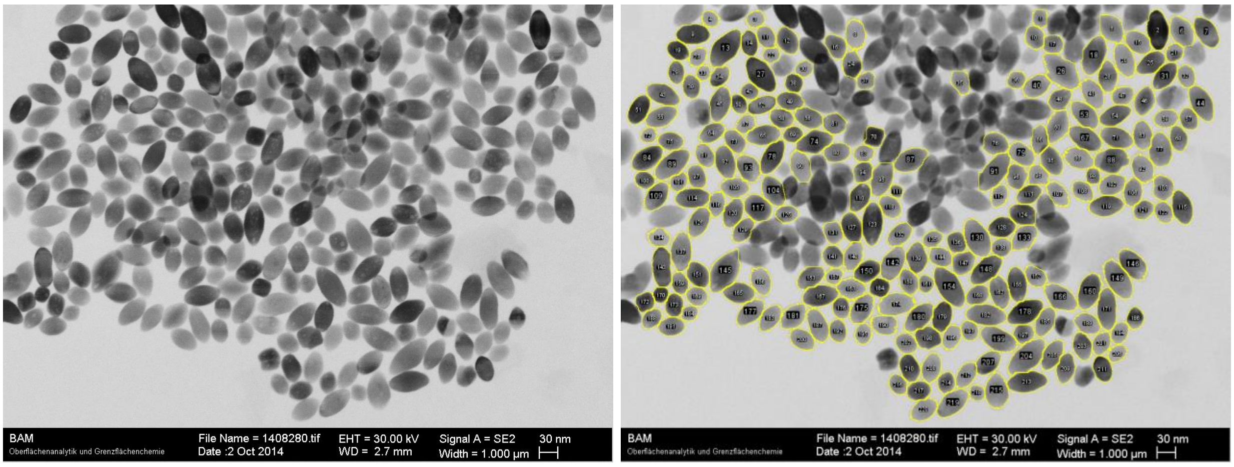

Figure S24. T-SEM images of sample HT17.

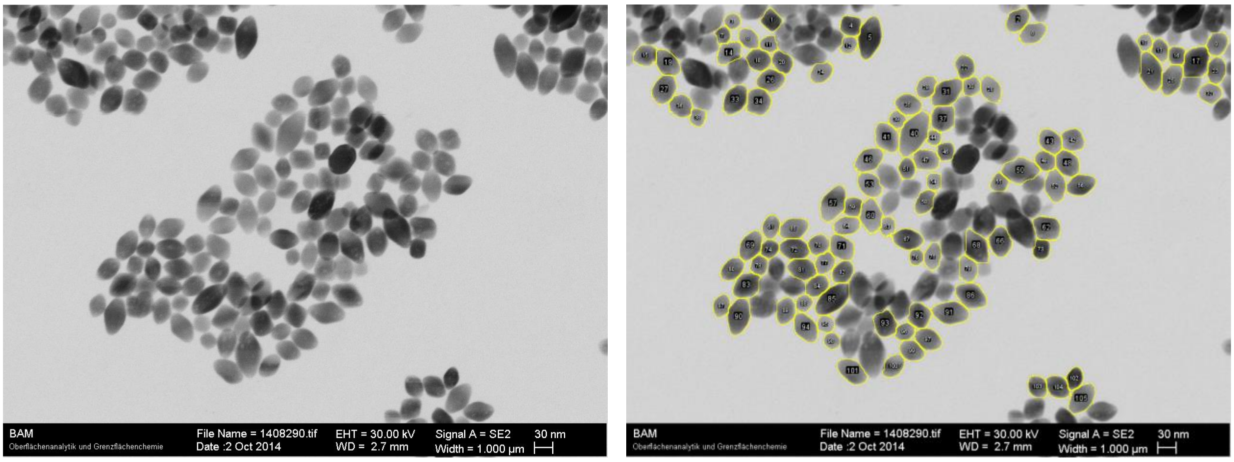

Figure S25. T-SEM images of sample HT18.

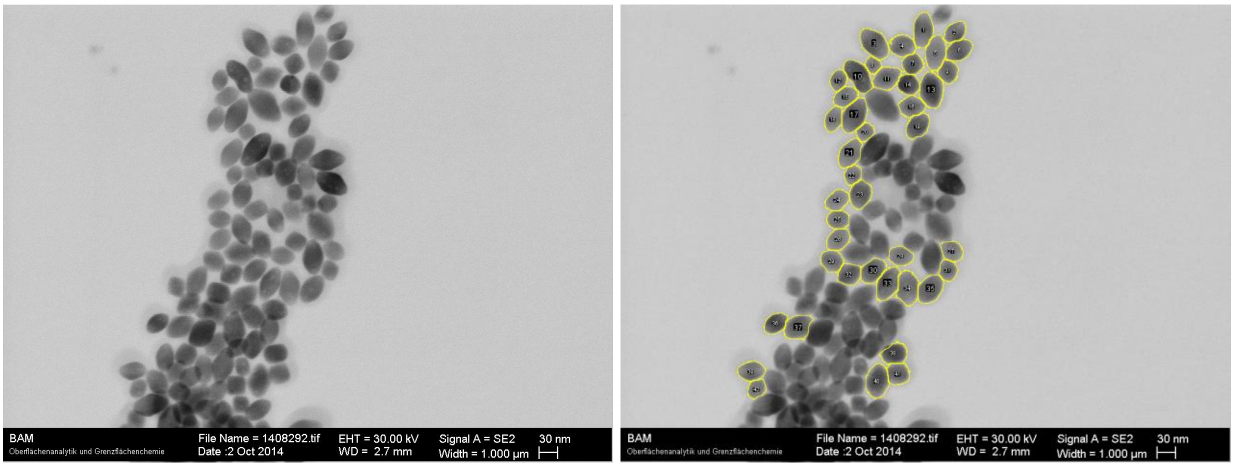

Figure S26. T-SEM images of sample HT19.

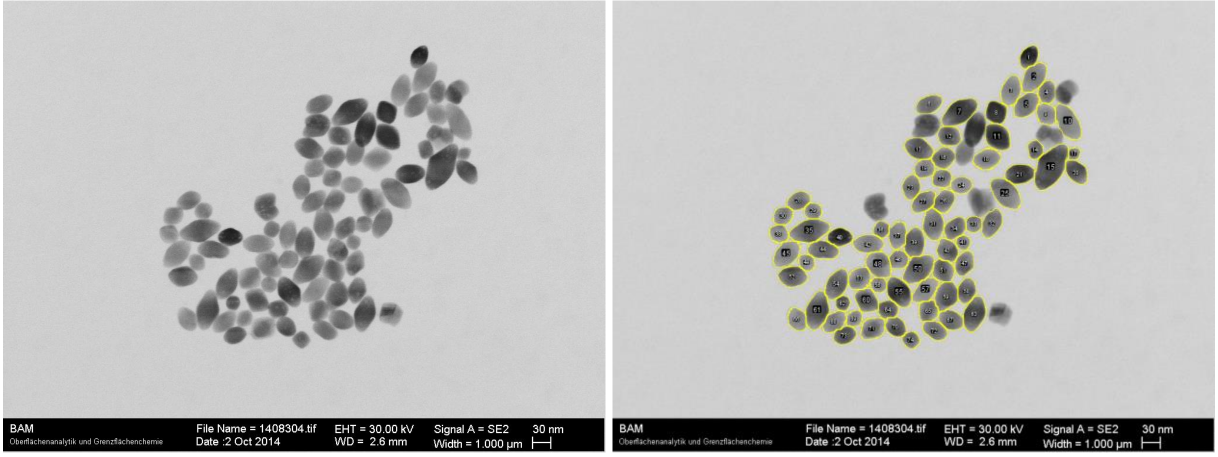

Figure S27. T-SEM images of sample HT20.

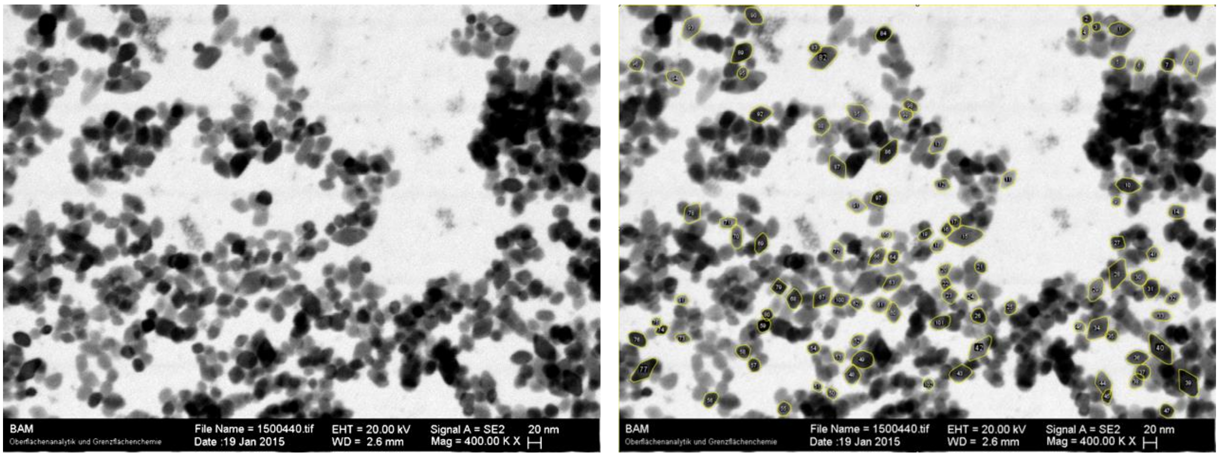

Figure S28. T-SEM images of sample HT-MODEL_01.

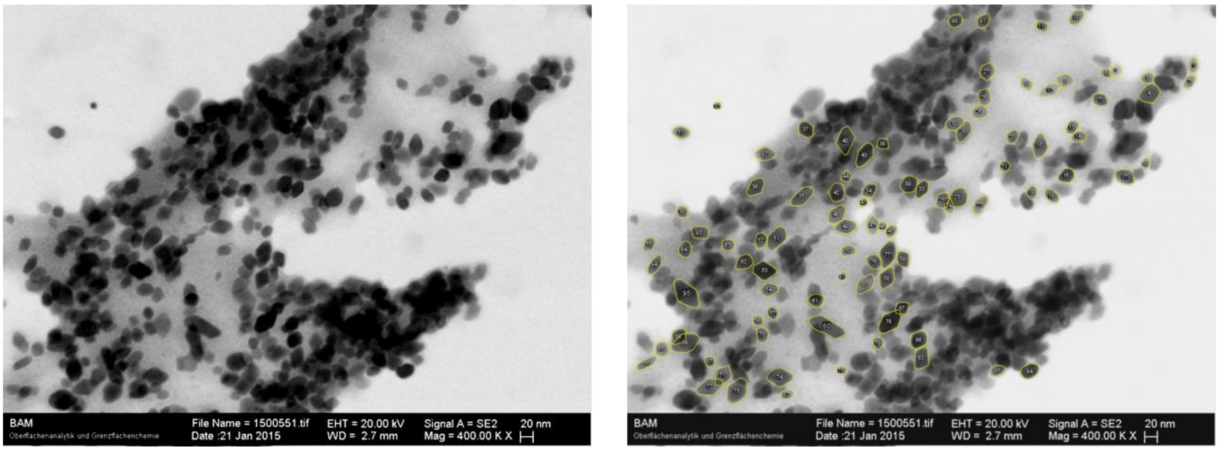

Figure S29. T-SEM images of sample HT-MODEL_02.

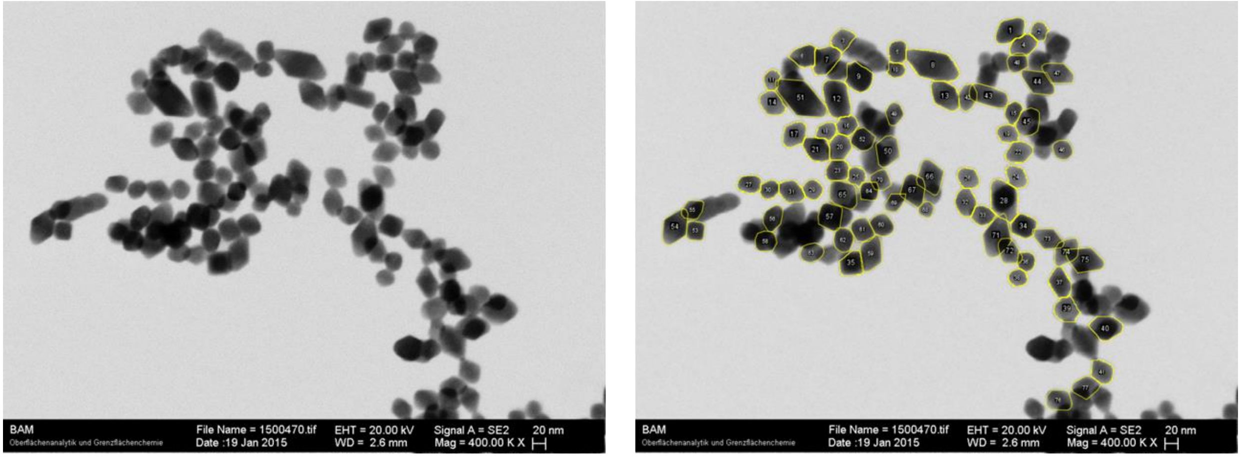

Figure S30. T-SEM images of sample HT-MODEL_03.

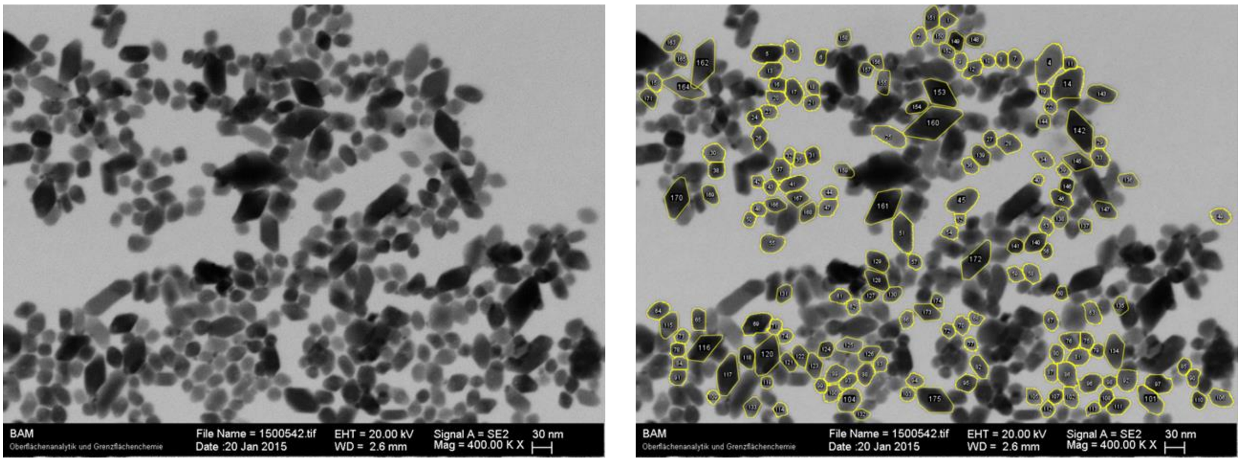

Figure S31. T-SEM images of sample HT-MODEL_04.

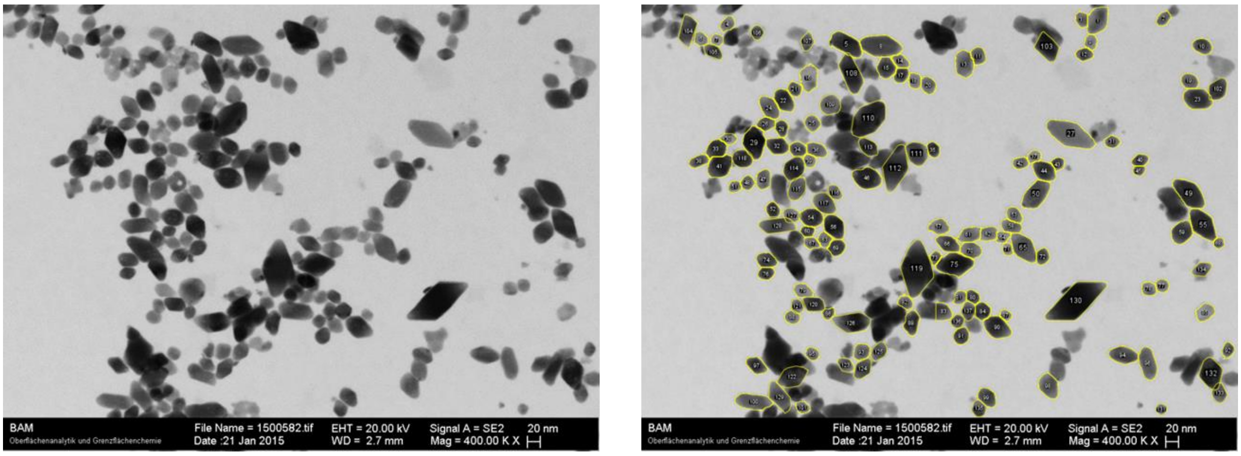

Figure S32. T-SEM images of sample HT-MODEL_05.

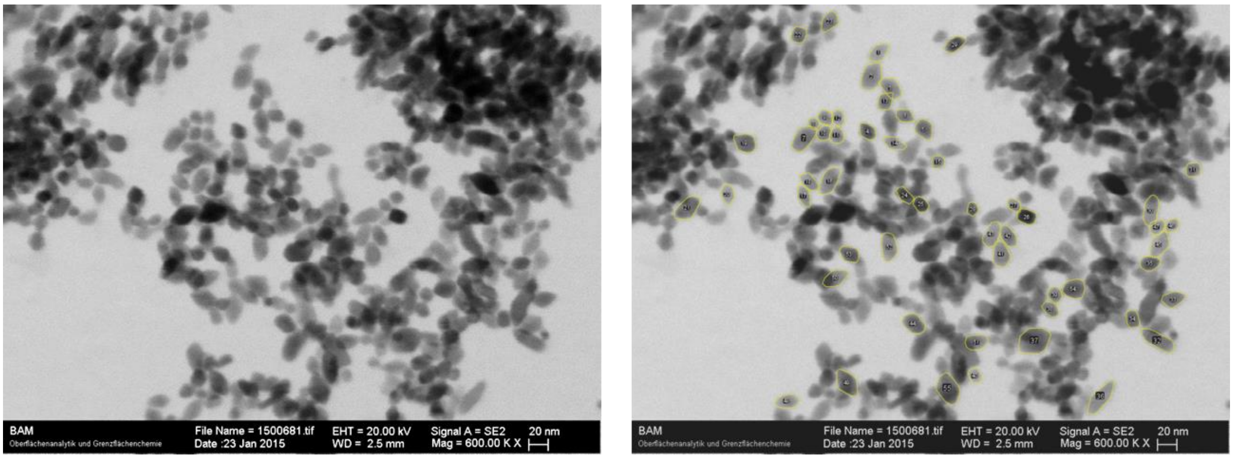

Figure S33. T-SEM images of sample HT-MODEL_06.

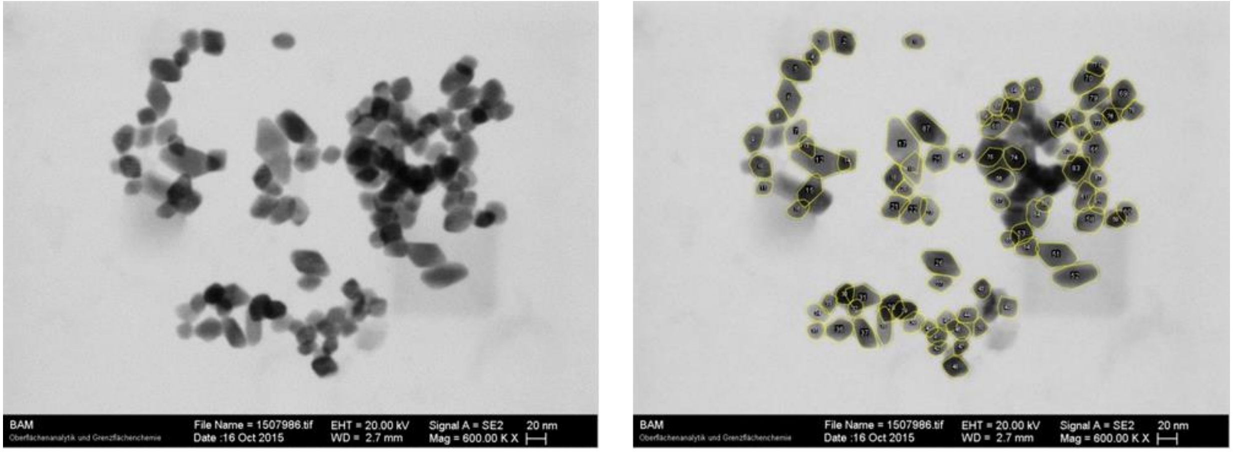

Figure S 34. T-SEM images of sample HT-MOD_BIS_01.

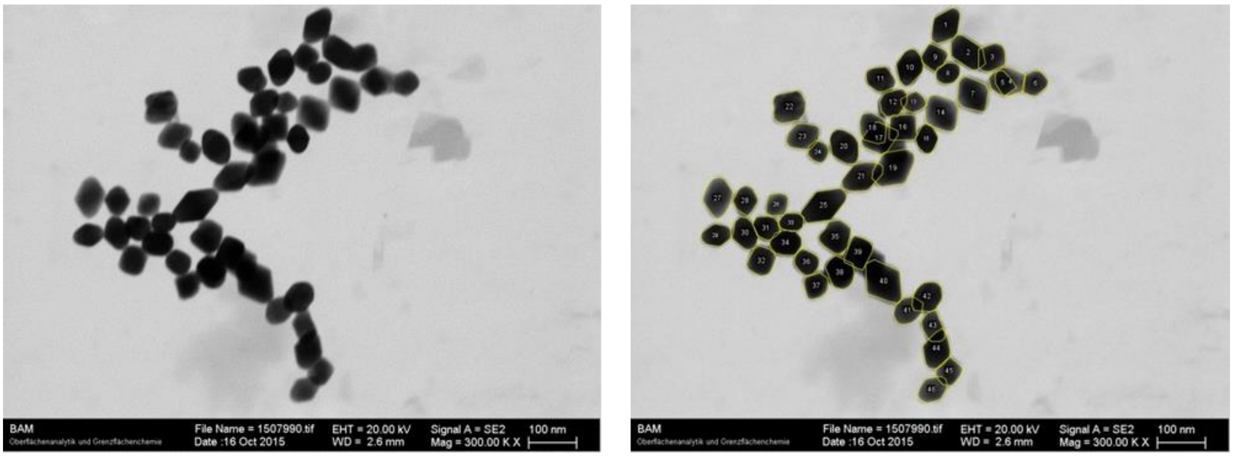

Figure S35. T-SEM images of sample HT-MOD_BIS_02.

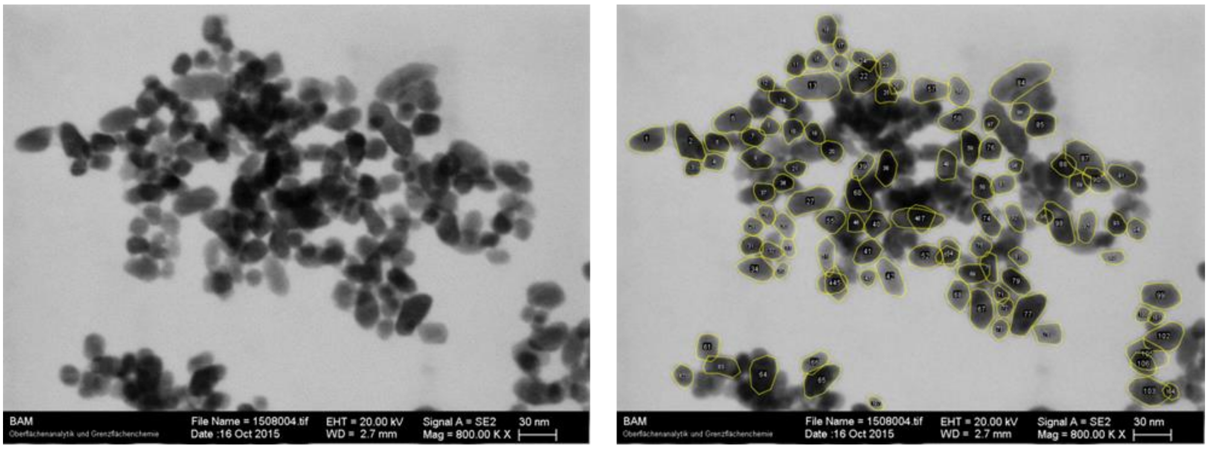

Figure S36. T-SEM images of sample HT-MOD_BIS_03.

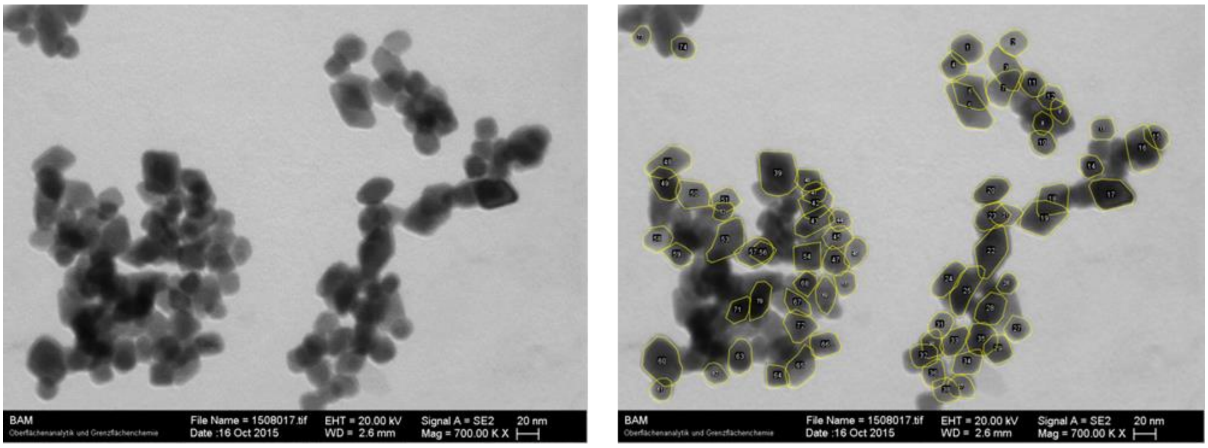

Figure S37. T-SEM images of sample HT-MOD_BIS_04.

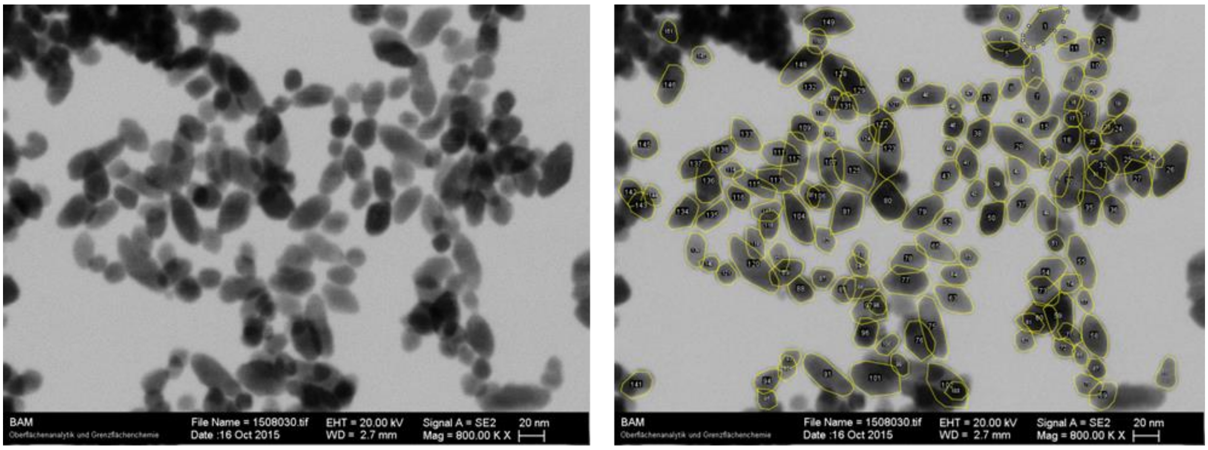

Figure S38. T-SEM images of sample HT-MOD_BIS_05.

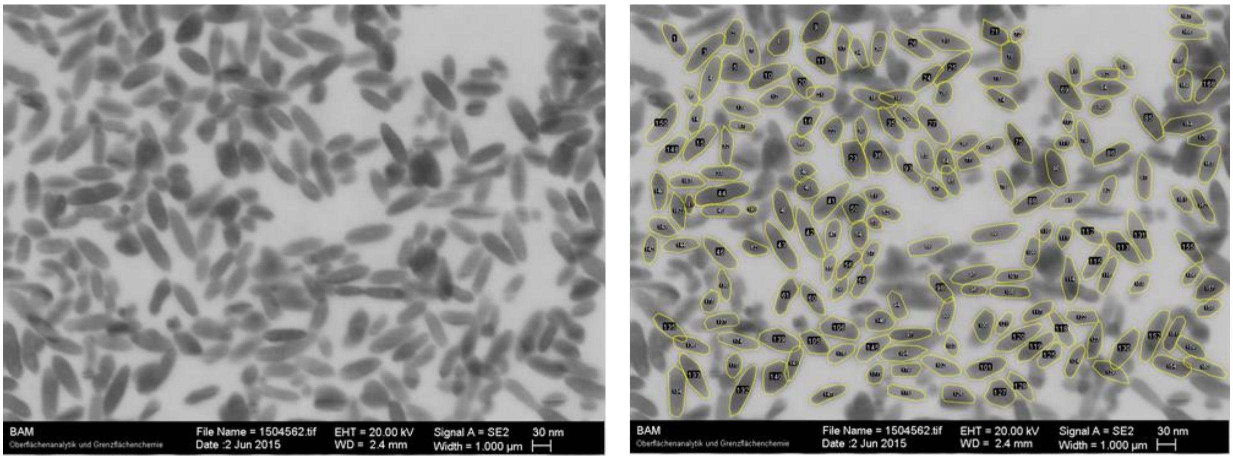

Figure S39. T-SEM images of sample HT-AspectRatio_01.

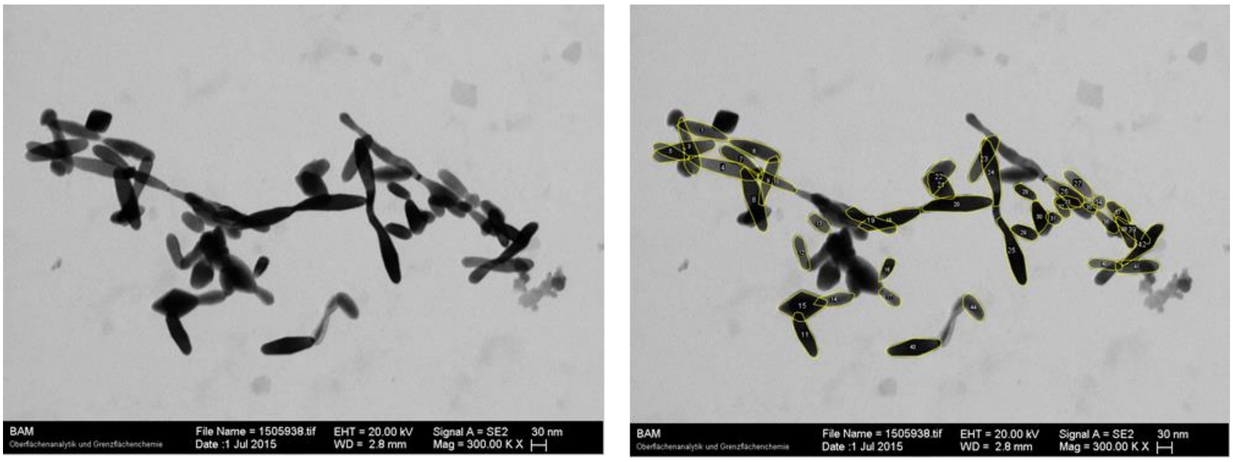

Figure S40. T-SEM images of sample HT-AspectRatio_02.

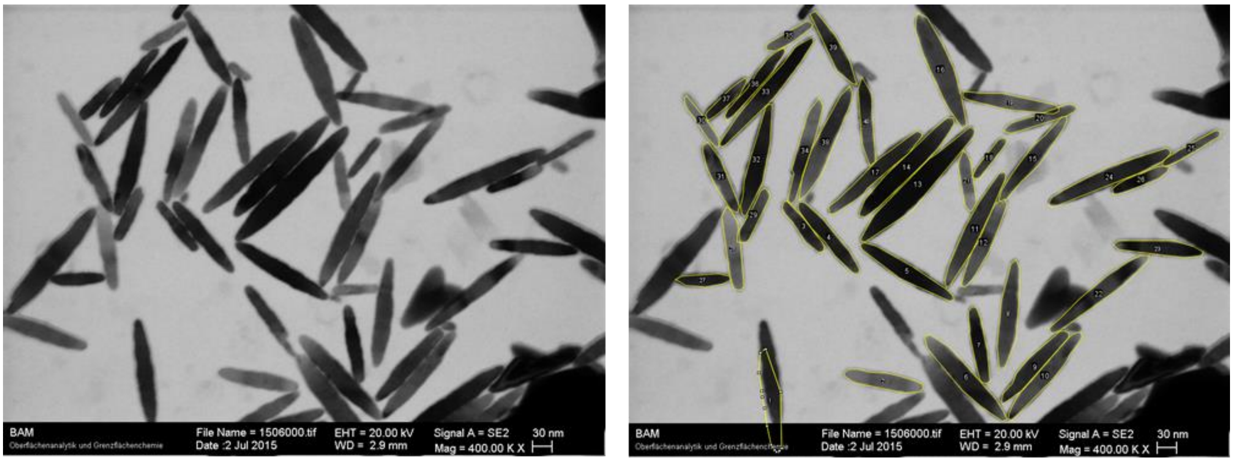

Figure S 41. T-SEM images of sample HT-AspectRatio_03.

7. Size determination by TSEM Analysis

The histogram representations of the size (major and minor, see Table S3) obtained by electron microscopy for all the samples synthetized in the manuscript are reported from Figure S42 to Figure S47.

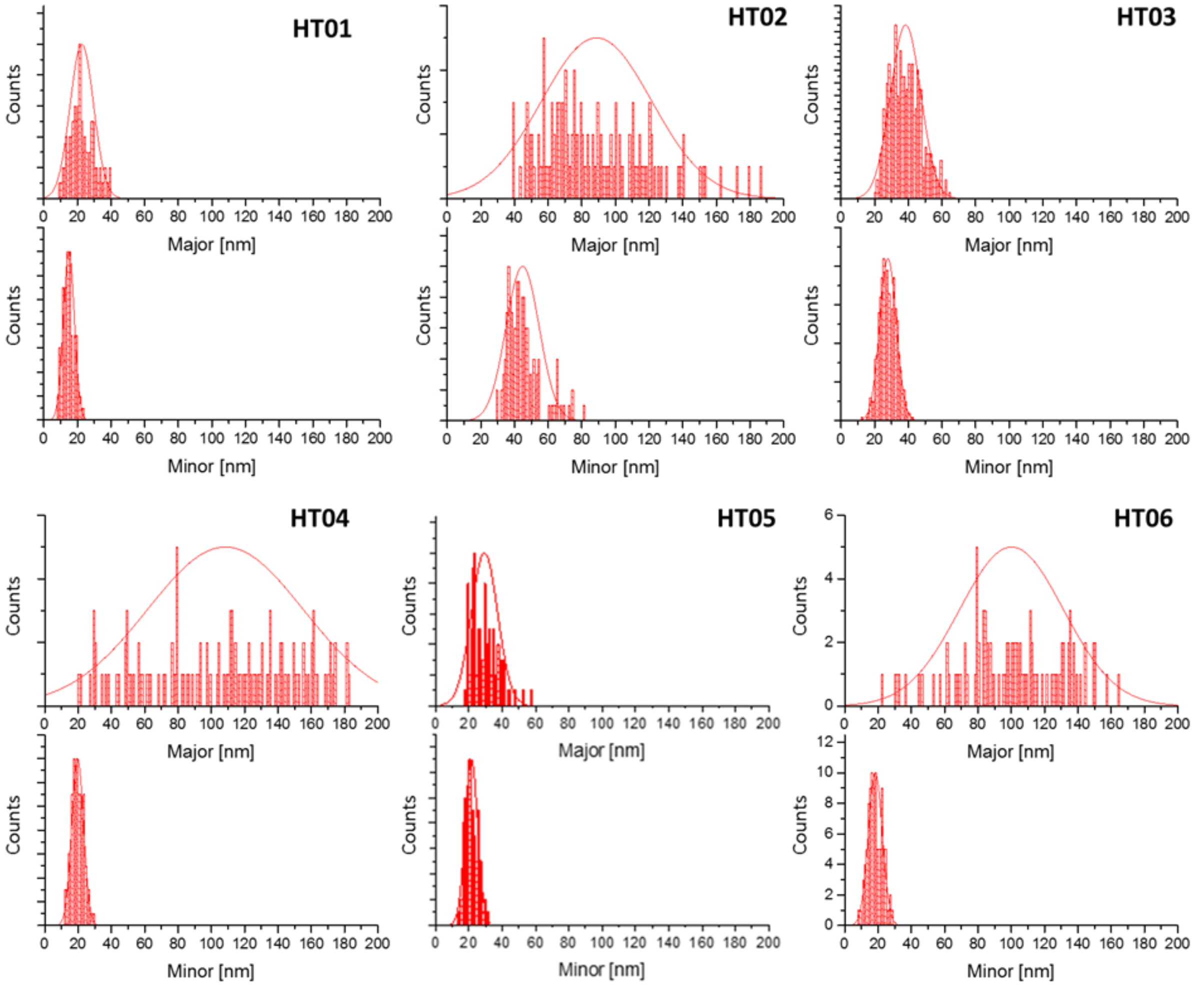

Figure S42. Histogram of the size (major and minor) obtained by TSEM analysis for the materials: HT01, HT02, HT03, HT04, HT05 and HT06.

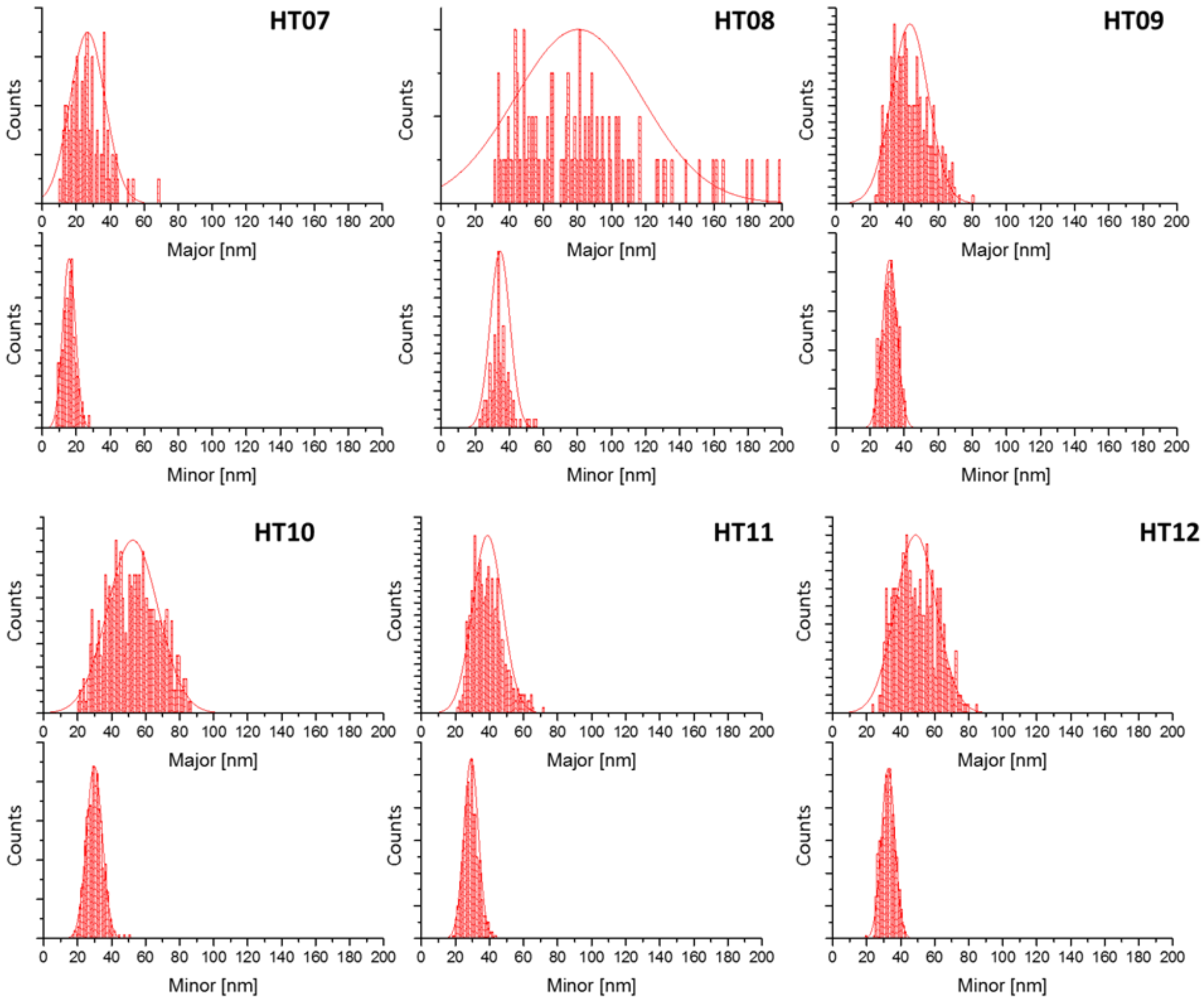

Figure S43. Histogram of the size (major and minor) obtained by TSEM analysis for the materials: HT07, HT08, HT09, HT10, HT11 and HT12.

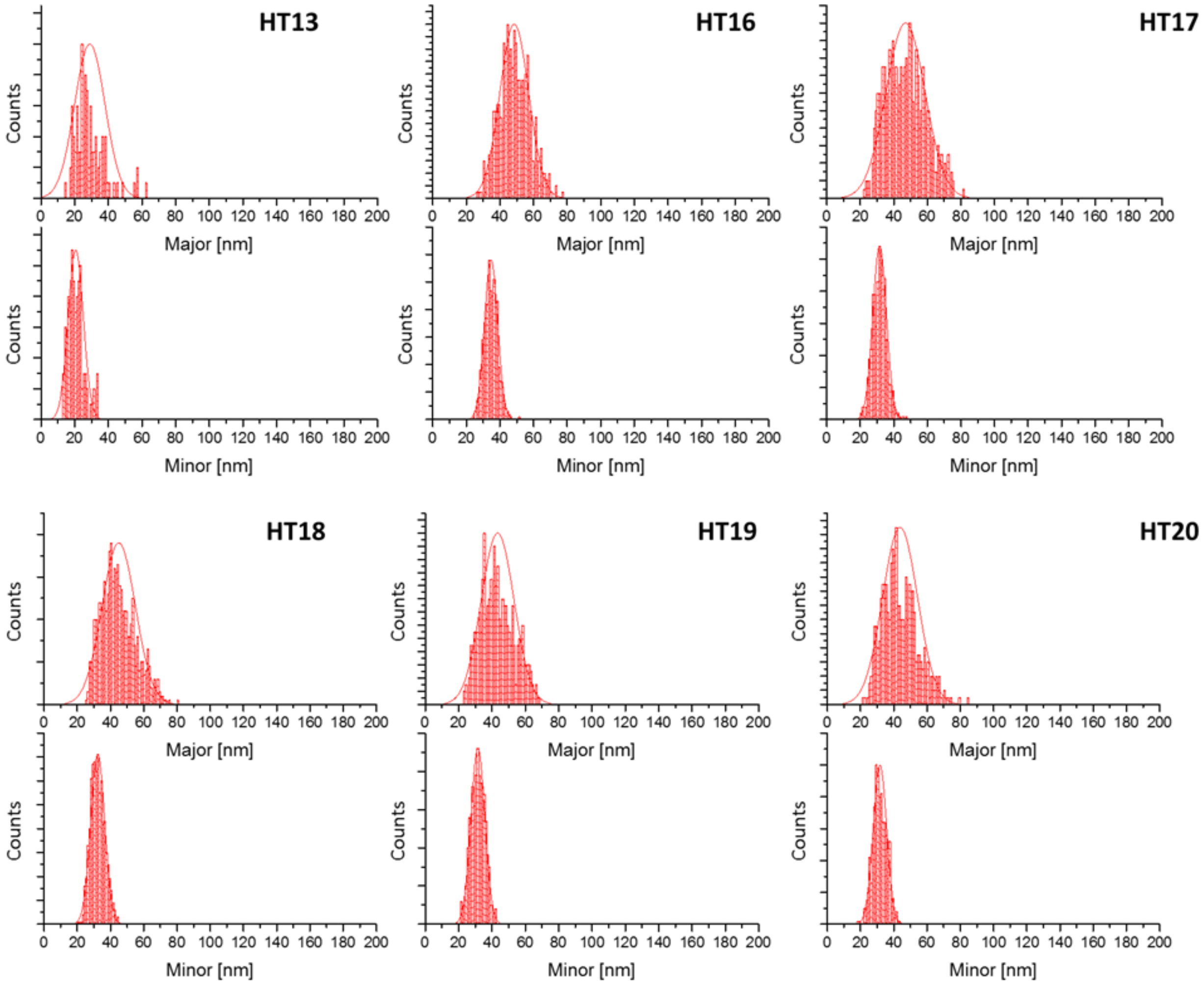

Figure S44. Histogram of the size (major and minor) obtained by TSEM analysis for the materials: HT13, HT16, HT17, HT18, HT19 and HT20.

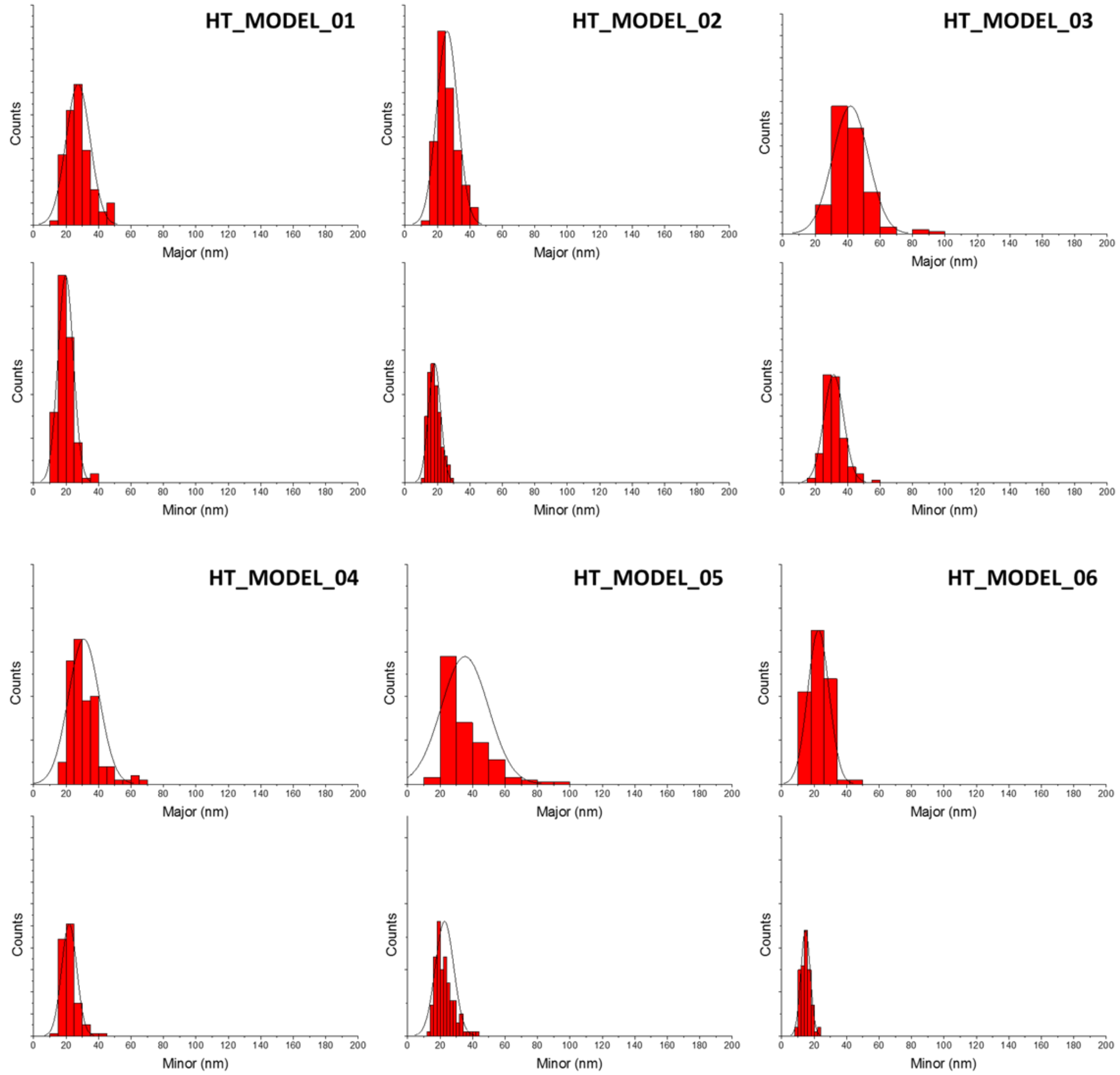

Figure S45. Histogram of the size (major and minor) obtained by TSEM analysis for the materials: HT_MODEL_01, HT_MODEL_02, HT_MODEL_03, HT_MODEL_04, HT_MODEL_05, HT_MODEL_06.

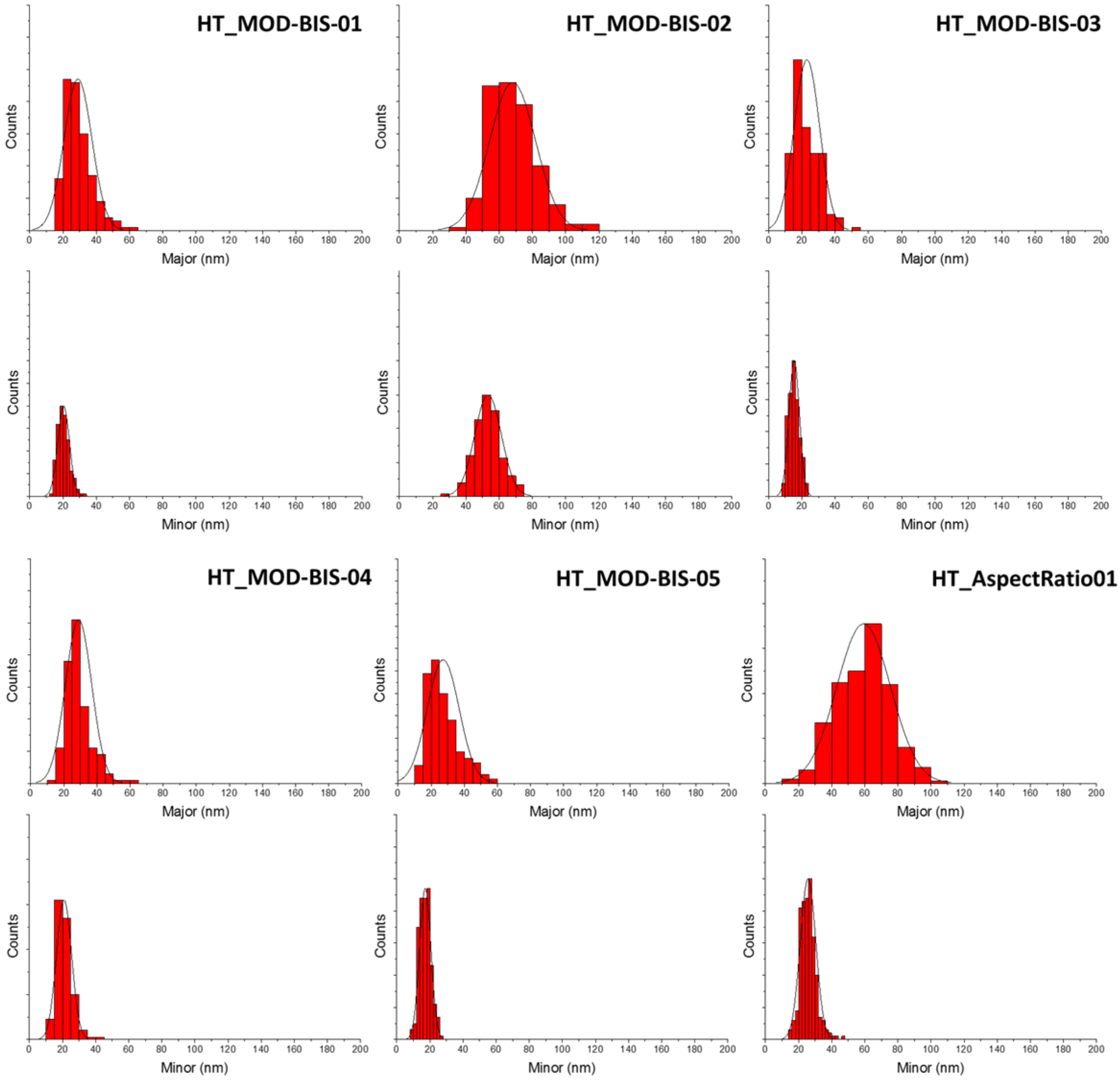

Figure S46. Histogram of the size (major and minor) obtained by TSEM analysis for the materials: HT_MOD-BIS-01, HT_MOD-BIS-02, HT_MOD-BIS-03, HT_MOD-BIS-04, HT_MOD-BIS-05, HT_AspectRatio01.

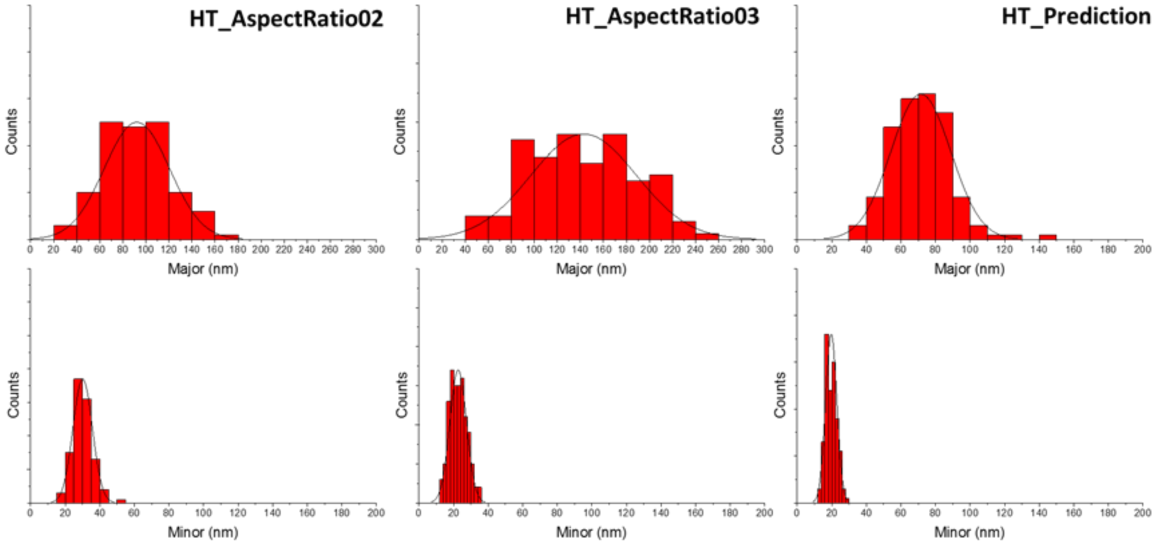

Figure S47. Histogram of the size (major and minor) obtained by TSEM analysis for the materials: HT_AspectRatio02, HT_AspectRatio03, HT_Prediction.

8. X-Ray Diffraction pattern analysis

Hereafter the X-Ray Diffraction patterns of the analyzed materials are reported; the flat configuration was used, except for the HT09 for which the capillary configuration was used. The crystallographic phase of the HT18, HT19 and HT20 materials was not determined, given that they are the result of three repetitions of the 17th experiment**.** In Table S2 in SI the X-ray diffraction analysis results are summarized. All the samples obtained were nanocrystalline, as evidenced by XRD, with anatase as the only detectable or the prominent crystalline phase, depending on the process parameters. The materials synthesized at an initial pH ≤ 10 contain only the anatase phase. The materials synthesized at an initial pH between 11 and 12 contain anatase along with low amounts of brookite, with the exception of the HT02 material and of the HT08 material. These two materials were synthesized at 203 °C, whereas those containing detectable traces of brookite were synthesized at 137 °C. At an initial pH ≥ 12 the brookite content was substantial (> 15% for HT14, 170 °C), so pH > 12 and temperatures < 170 °C favour the formation of the brookite phase. Brookite could come from the large concentration of Na^+, NaOH was used for adjusting the pH. Na^+ stabilizes the brookite polymorph.[12] The material HT15 shows XRD reflexes broader than those corresponding to the other materials, confirming the results of DLS measurements (R_H of primary particles under 2 nm, see Table S4). The amount of brookite in the samples HT04, HT06 and HT14 was determined by the Rietveld analysis carried out by using the MAUD (Materials Analysis Using Diffraction) software and anatase and brookite XRD patterns from Crystallography Open Database (COD). From the XRD patterns, the average sizes of the crystal domains were also obtained by means of the Scherrer Analysis of the 004 XRD reflex (Table S3, D_{004}). This parameter, compared with the parameter *Max* (major), allows an independent estimation of the crystal domain size along c-axes and its coherence with the NP length measured by electron microscopy. Table S3 highlights that a substantial discrepancy is found for the material with an aspect ratio (*p*) larger than 2 only (HT04, HT06 and HT08), indicating that there is a low defectivity of the crystal lattice along the c-axis only for NPs with low aspect ratio. This conclusion can have an important impact on the chemical-physical characteristics of the nanoparticles (facets indexing, carriers' dynamics, etc.) and, therefore, on their functional properties. Little discrepancies can be attributed also to the underestimation of the "major" values in the microscopic analysis, as explained in a previous paragraph. This highlights that even though a certain amount of elongation for monocrystalline NPs is possible, this is limited to aspect ratio values up to 2. Further elongation is associated to the existence of more than one crystalline domain in the NP. Figure S48 and Figure S49 report the reference XRD patterns (together with the peak list) of anatase and brookite respectively.

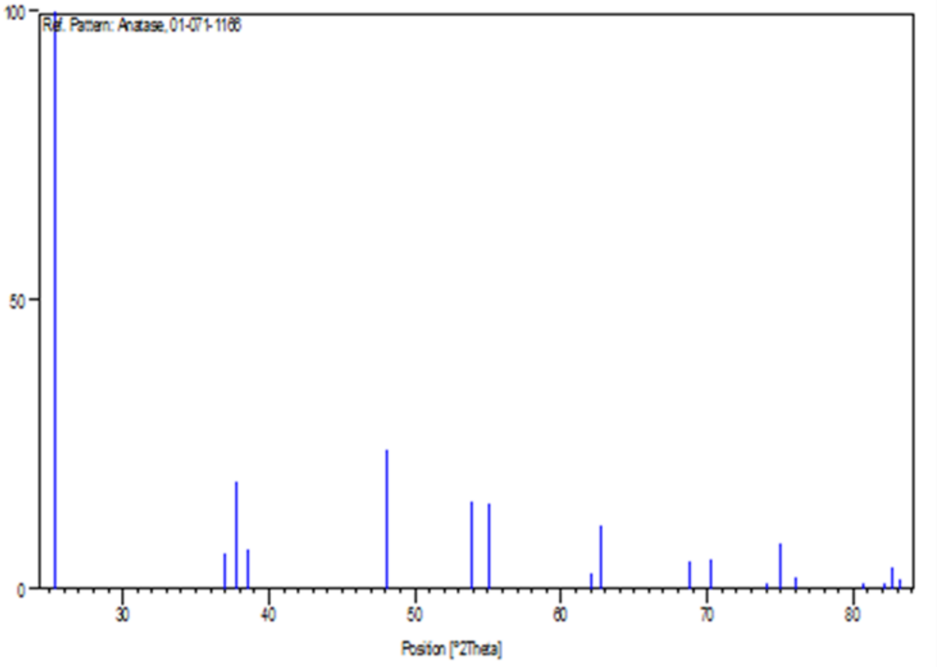

Peak list

No.	h	k	l	d [A]	2Theta[deg]	I [%]
1	1	0	1	3.51629	25.308	100.0
2	1	0	3	2.43073	36.951	6.1
3	0	0	4	2.37865	37.791	18.6
4	1	1	2	2.33222	38.572	7.0
5	2	0	0	1.89210	48.047	24.2
6	1	0	5	1.70007	53.885	15.0
7	2	1	1	1.66619	55.073	14.8
8	2	1	3	1.49308	62.117	2.6
9	2	0	4	1.48076	62.692	11.0
10	1	1	6	1.36420	68.756	4.7
11	2	2	0	1.33792	70.304	5.1
12	1	0	7	1.27921	74.051	0.5
13	2	1	5	1.26459	75.053	7.7
14	3	0	1	1.25046	76.051	2.1
15	0	0	8	1.18933	80.733	0.4
16	3	0	3	1.17210	82.173	0.5
17	2	2	4	1.16611	82.687	3.8
18	3	1	2	1.16052	83.174	1.6

Figure S48. Reference XRD pattern and peak list of Anatase (from JCPDS 01-071-1166).

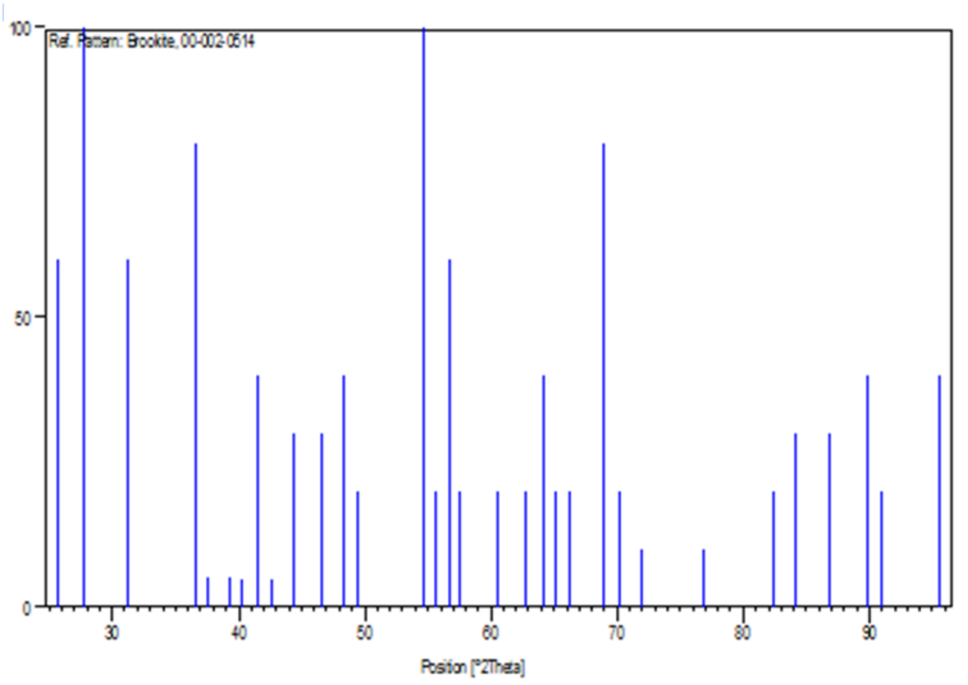

Peak list

No.	h	k	l	d [A]	2Theta[deg]	I [%]
1	1	1	1	3.46000	25.727	60.0
2				3.22000	27.681	100.0
3	2	1	1	2.87000	31.138	60.0
4	1	0	2	2.45000	36.650	80.0
5	0	2	1	2.39000	37.604	5.0
6	4	0	0	2.29000	39.312	5.0
7	2	0	2	2.24000	40.227	5.0
8				2.17000	41.584	40.0
9	2	2	1	2.12000	42.612	5.0
10				2.04000	44.370	30.0
11	4	1	1	1.95000	46.535	30.0
12	3	2	1	1.88000	48.376	40.0
13	3	1	2	1.84000	49.498	20.0
14	1	3	1	1.68000	54.582	100.0
15	5	1	1	1.65000	55.660	20.0
16	4	1	2	1.62000	56.783	60.0
17	2	3	1	1.60000	57.559	20.0
18	6	0	0	1.53000	60.459	20.0
19	6	1	0	1.48000	62.728	20.0
20	0	2	3	1.45000	64.179	40.0
21	1	2	3	1.43000	65.186	20.0
22	2	3	2	1.41000	66.229	20.0
23	0	4	0	1.36000	68.999	80.0
24	3	3	2	1.34000	70.178	20.0
25	3	2	3	1.31000	72.033	10.0
26	1	1	4	1.24000	76.809	10.0
27	4	4	0	1.17000	82.352	20.0
28	7	2	1	1.15000	84.107	30.0
29	4	0	4	1.12000	86.907	30.0
30	4	3	3	1.09000	89.934	40.0
31				1.08000	90.998	20.0
32	7	3	1	1.04000	95.578	40.0

Figure S49. Reference XRD pattern and peak list of Brookite (from JCPDS 00-002-0514).

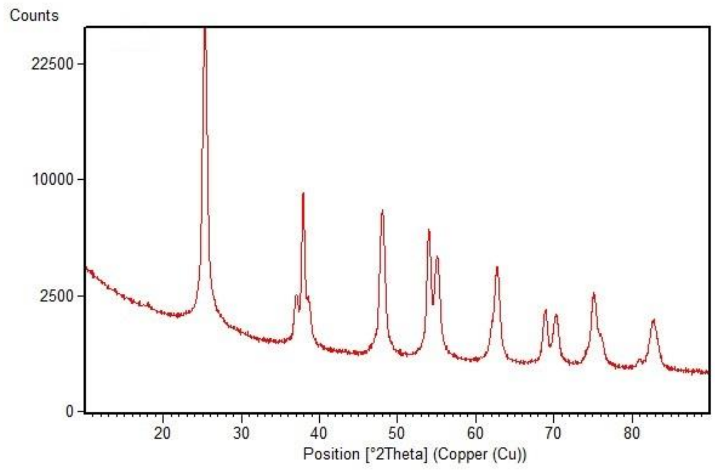

Figure S50. X-ray diffraction pattern of the TiO_2 HT01 material

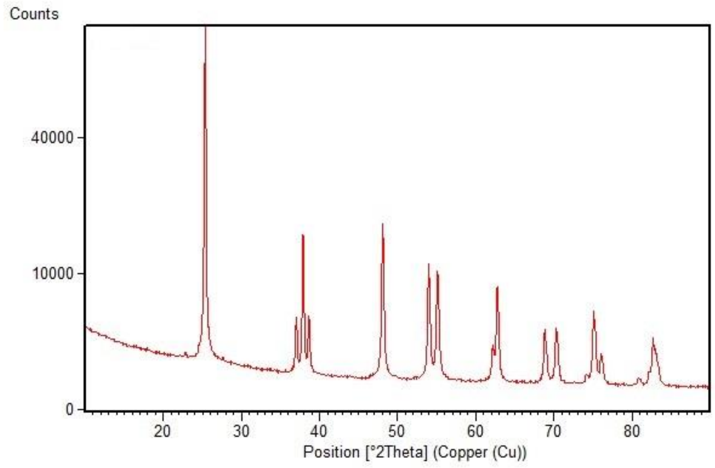

Figure S51. X-ray diffraction pattern of the TiO_2 HT02 material

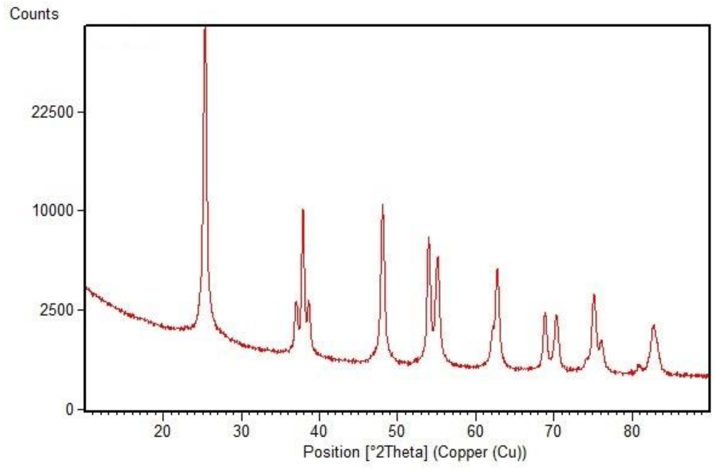

Figure S52. X-ray diffraction pattern of the TiO_2 HT03 material

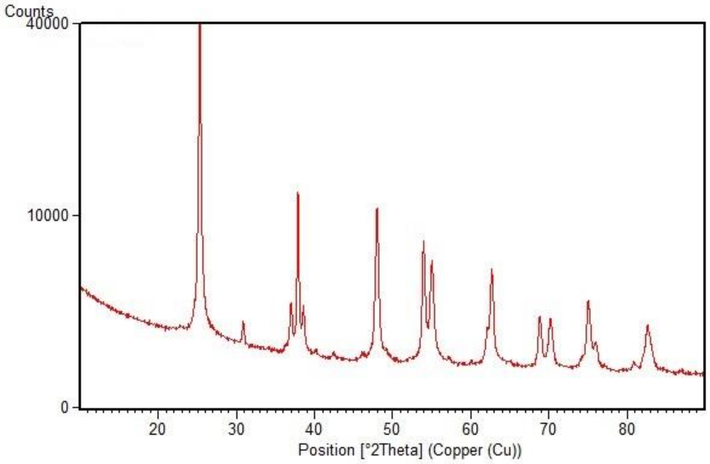

Figure S53. X-ray diffraction pattern of the TiO_2 HT04 material. Trace of brookite phase is visible.

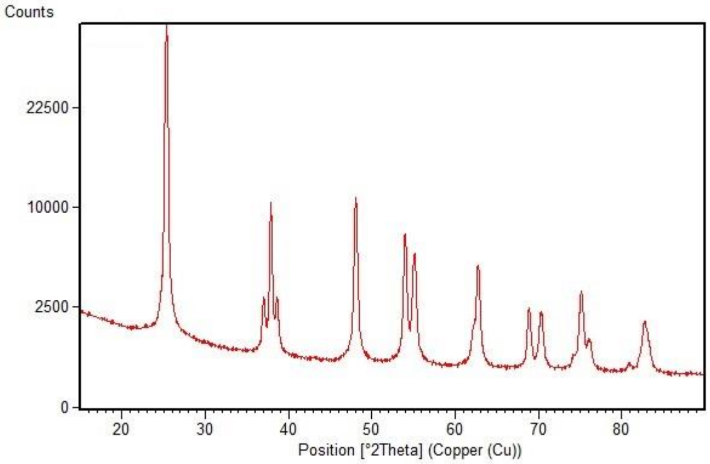

Figure S54. X-ray diffraction pattern of the TiO_2 HT05 material

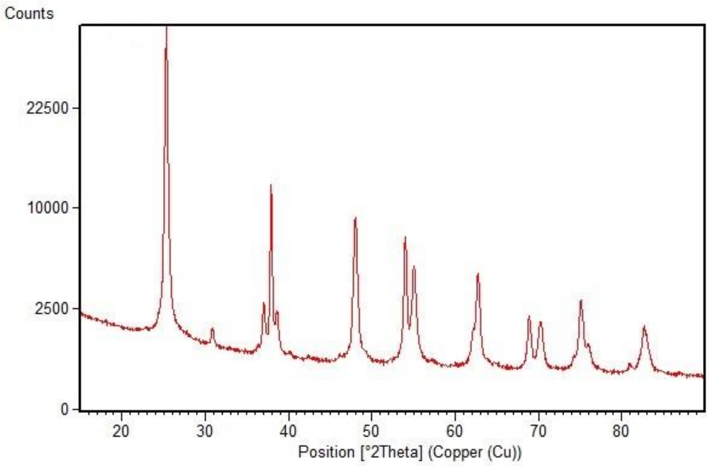

Figure S55. X-ray diffraction pattern of the TiO_2 HT06 material. Trace of brookite phase is visible.

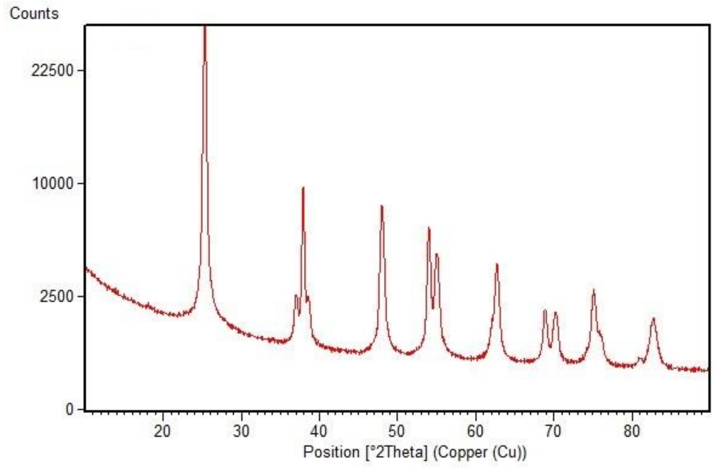

Figure S56. X-ray diffraction pattern of the TiO_2 HT07 material

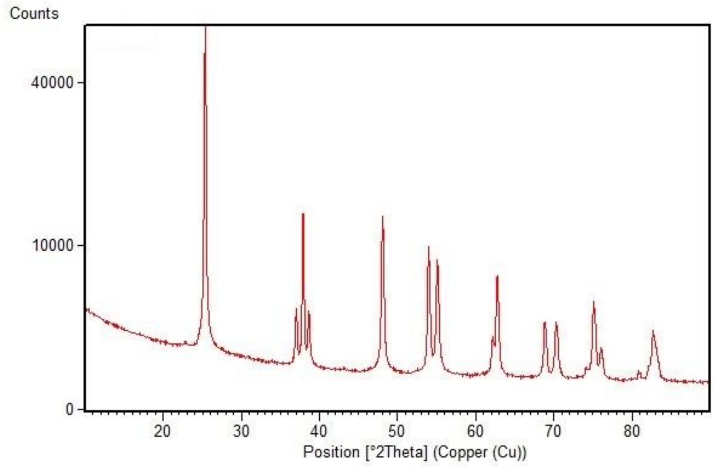

Figure S57. X-ray diffraction pattern of the TiO_2 HT08 material

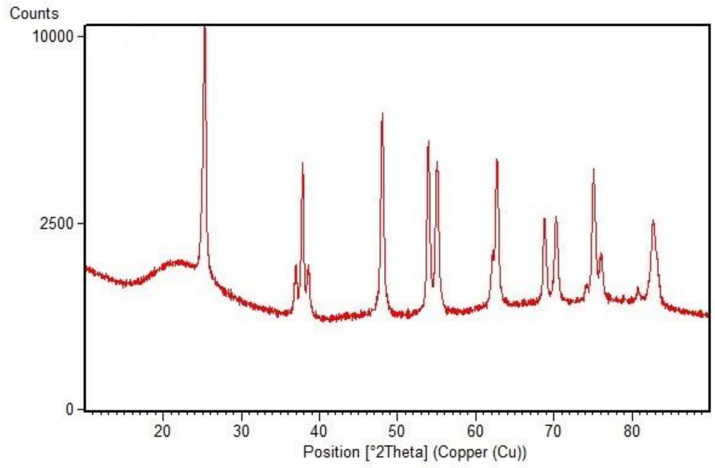

Figure S58. X-ray diffraction pattern of the TiO_2 HT09 material

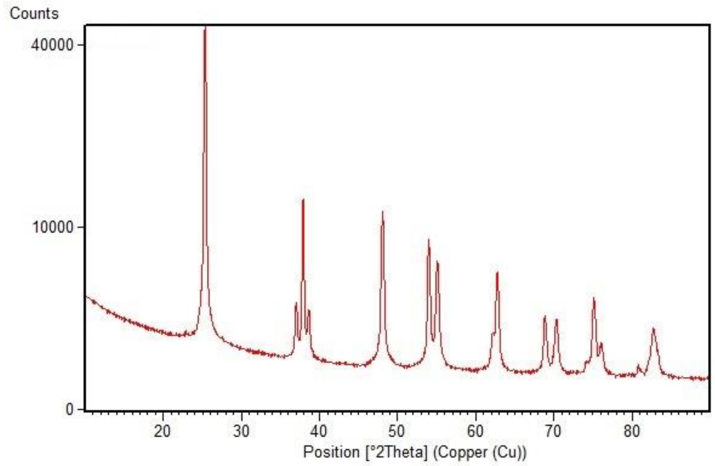

Figure S59. X-ray diffraction pattern of the TiO_2 HT10 material

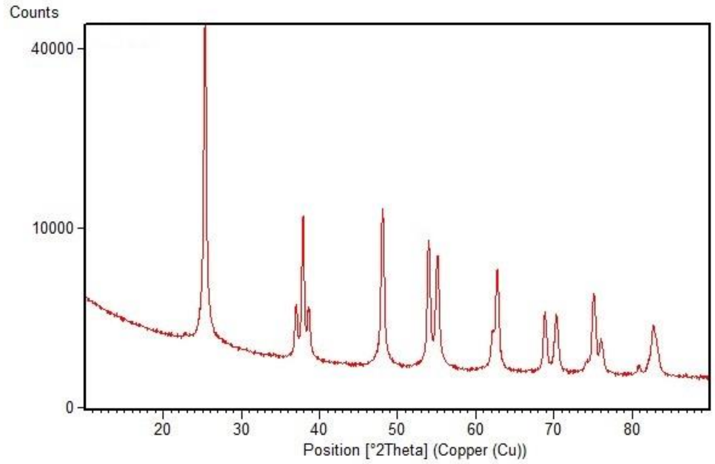

Figure S60. X-ray diffraction pattern of the TiO_2 HT11 material

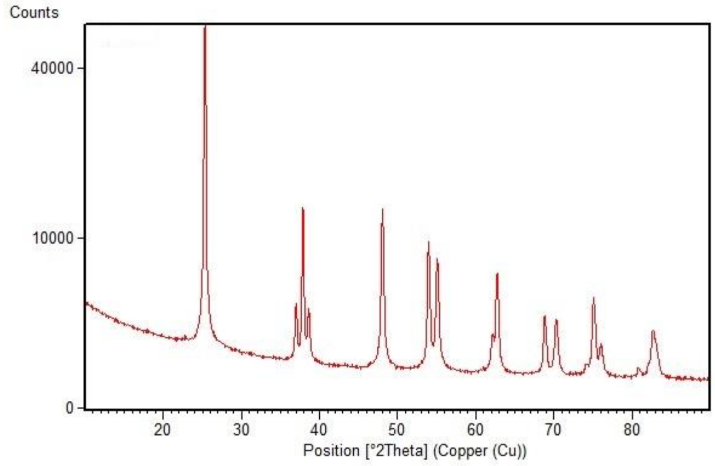

Figure S61. X-ray diffraction pattern of the TiO_2 HT12 material

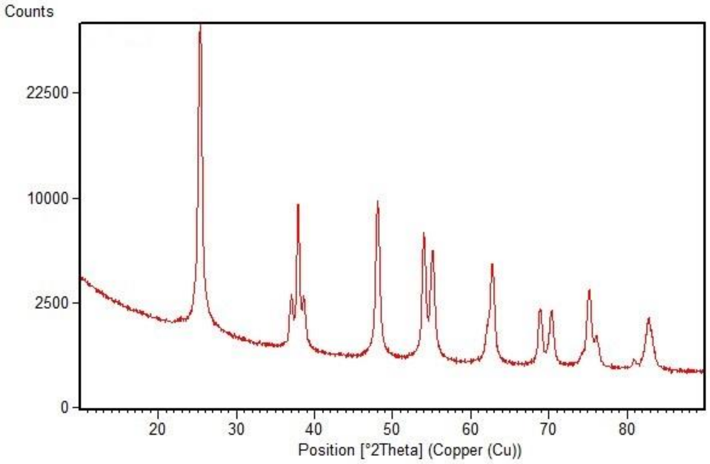

Figure S62. X-ray diffraction pattern of the TiO_2 HT13 material

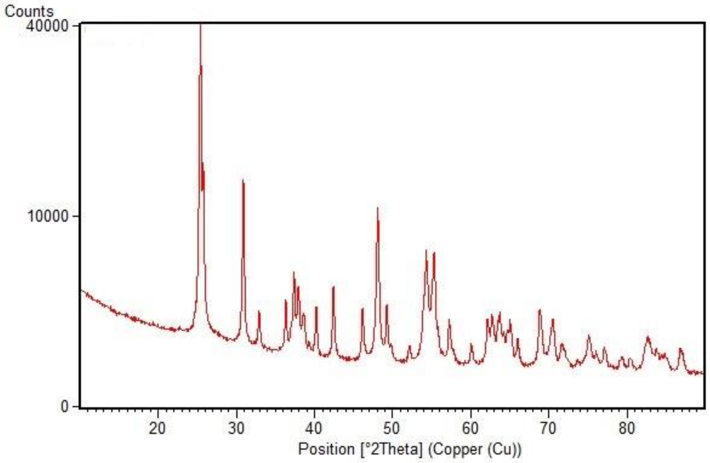

Figure S63. X-ray diffraction pattern of the TiO_2 HT14 material. Brookite phase is detected.

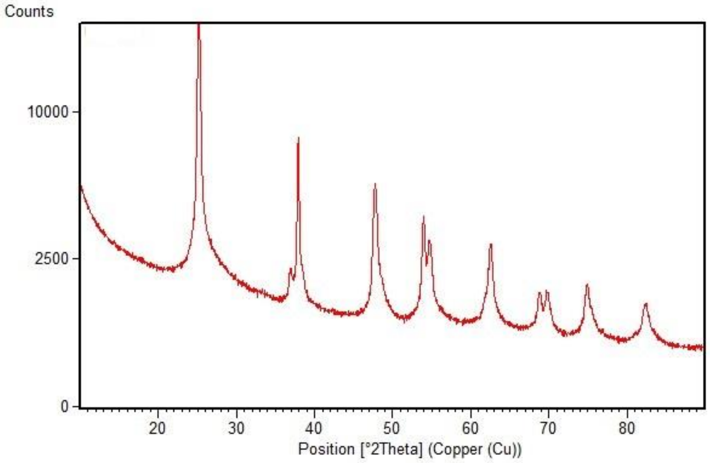

Figure S64. X-ray diffraction pattern of the TiO_2 HT15 material

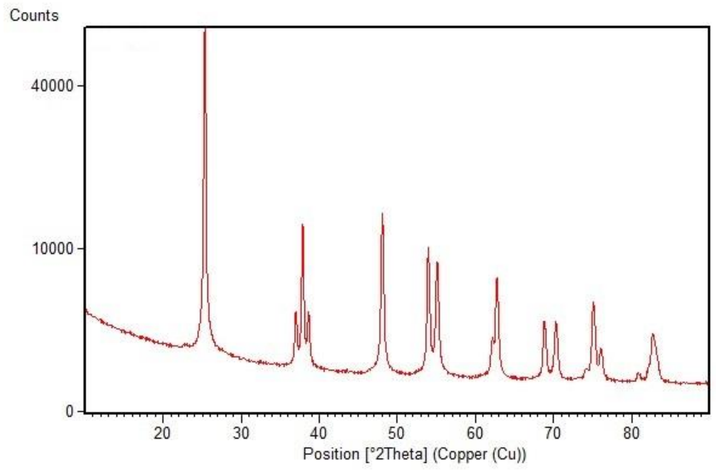

Figure S65. X-ray diffraction pattern of the TiO_2 HT16 material

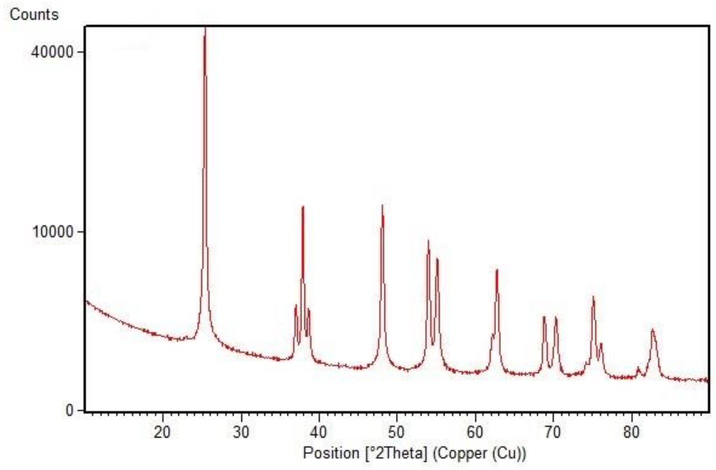

Figure S66. X-ray diffraction pattern of the TiO_2 HT17 material

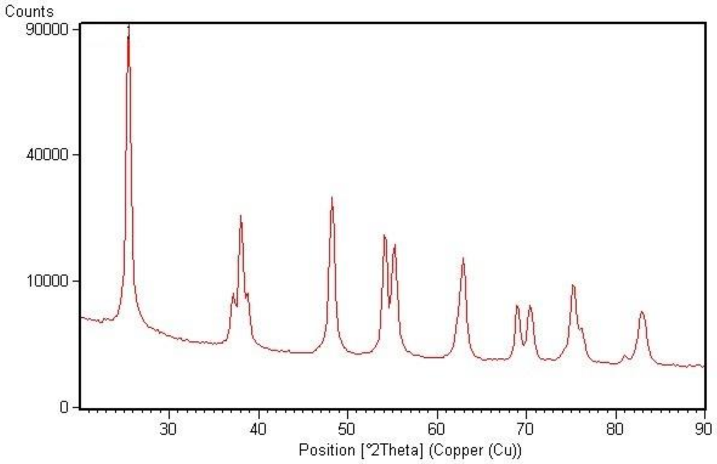

Figure S67. X-ray diffraction pattern of the TiO_2 HT-MODEL_01 material.

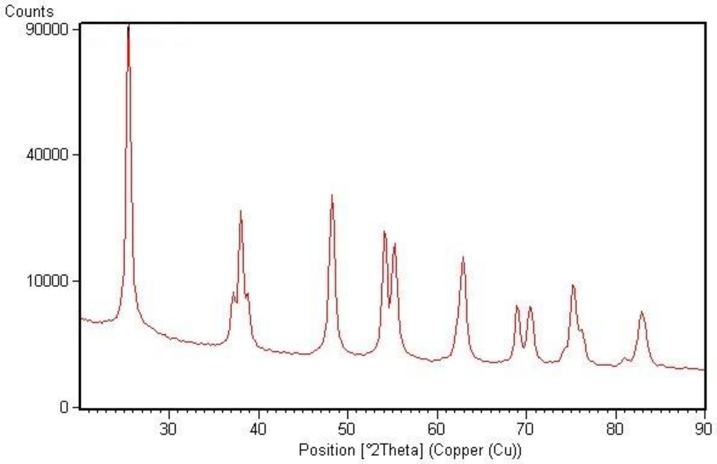

Figure S68. X-ray diffraction pattern of the TiO_2 HT-MODEL_02 material.

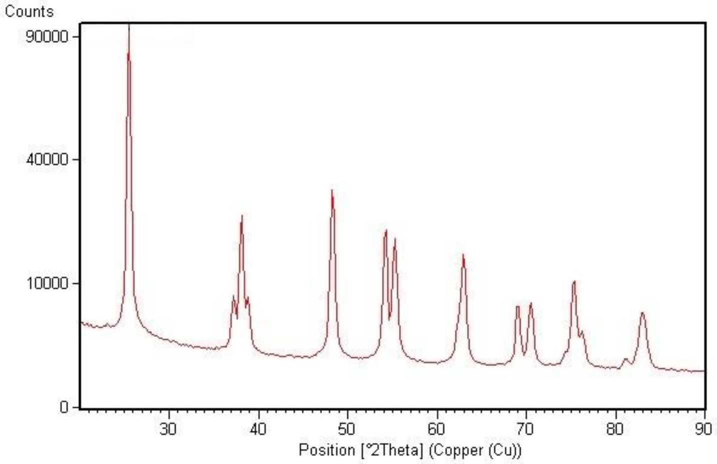

Figure S69. X-ray diffraction pattern of the TiO_2 HT-MODEL_03 material.

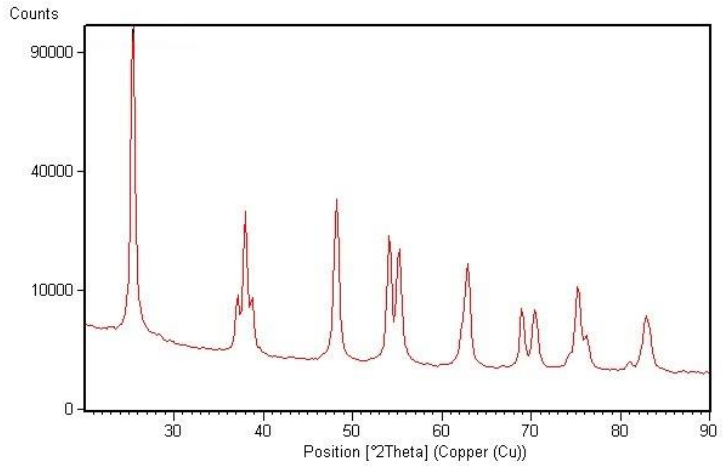

Figure S70. X-ray diffraction pattern of the TiO_2 HT-MODEL_04 material.

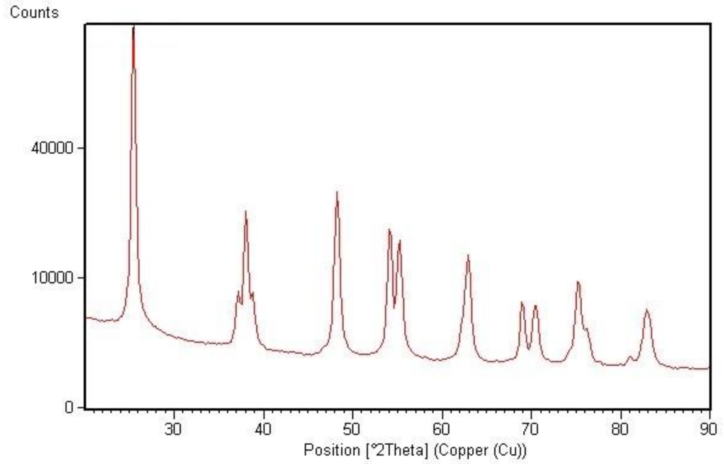

Figure S71. X-ray diffraction pattern of the TiO_2 HT-MODEL_05 material.

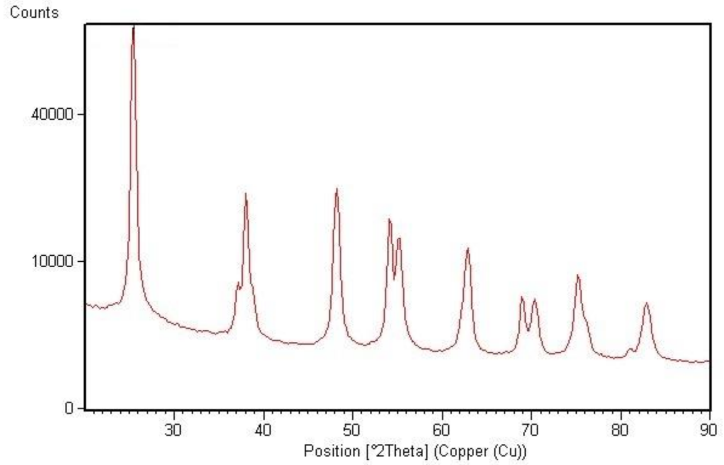

Figure S72. X-ray diffraction pattern of the TiO_2 HT-MODEL_06 material.

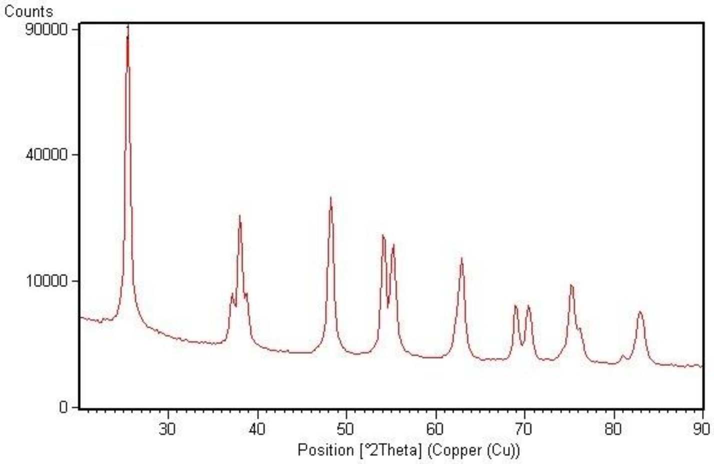

Figure S73. X-ray diffraction pattern of the TiO_2 HT-MOD_BIS_01 material.

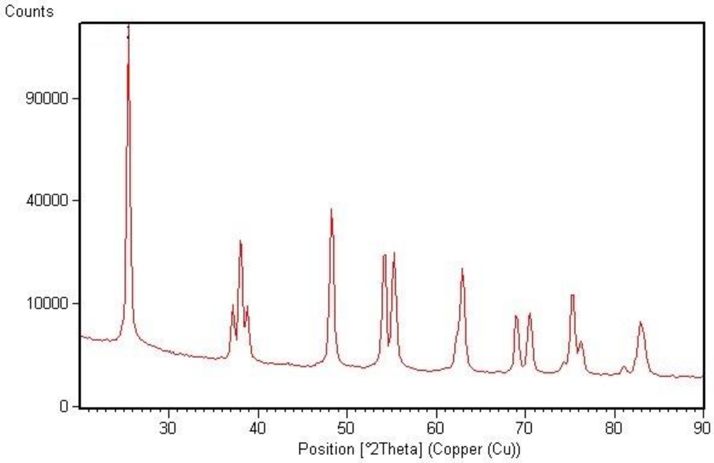

Figure S74. X-ray diffraction pattern of the TiO_2 HT-MOD_BIS_02 material.

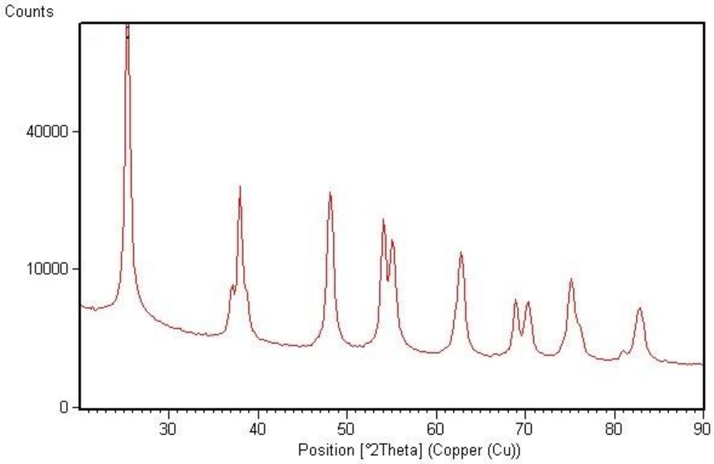

Figure S75. X-ray diffraction pattern of the TiO_2 HT-MOD_BIS_03 material.

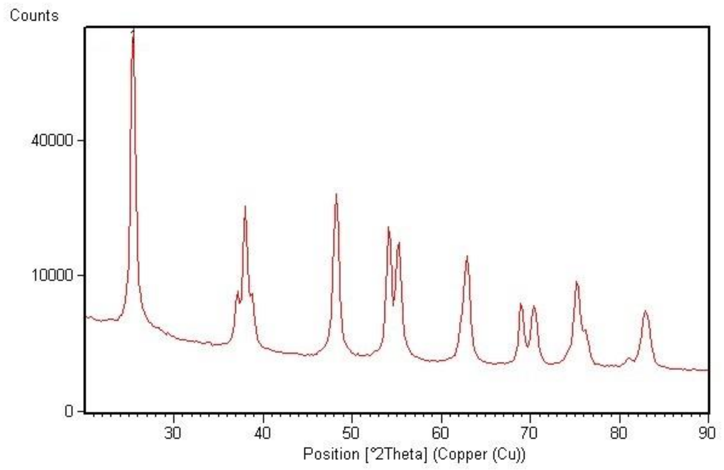

Figure S76. X-ray diffraction pattern of the TiO_2 HT-MOD_BIS_04 material.

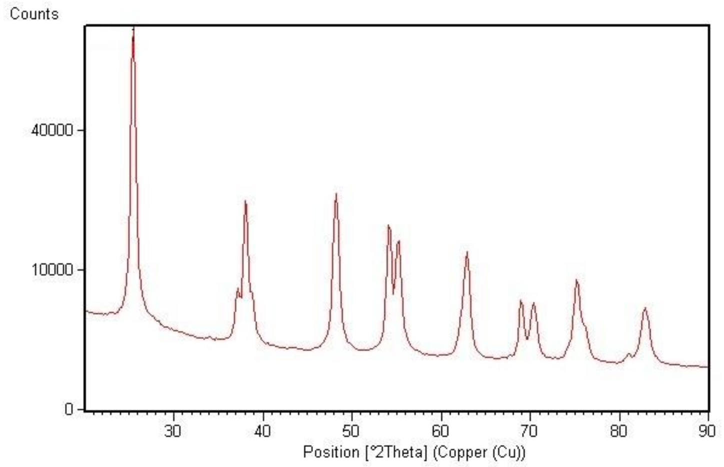

Figure S77. X-ray diffraction pattern of the TiO_2 HT-MOD_BIS_05 material.

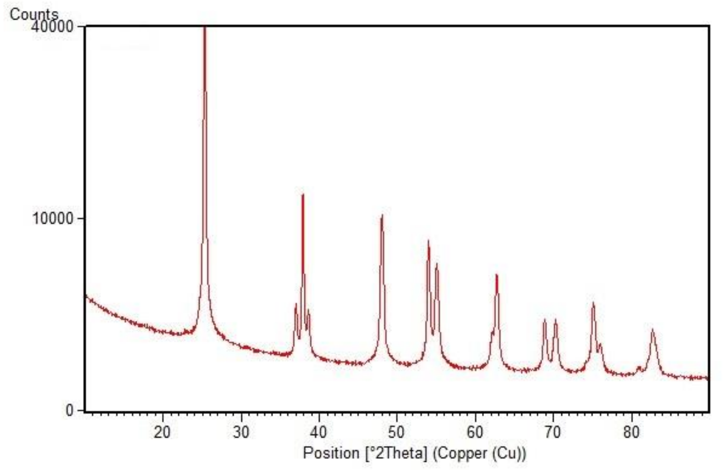

Figure S78. X-ray diffraction pattern of the TiO_2 HT-AspectRatio_01 material.

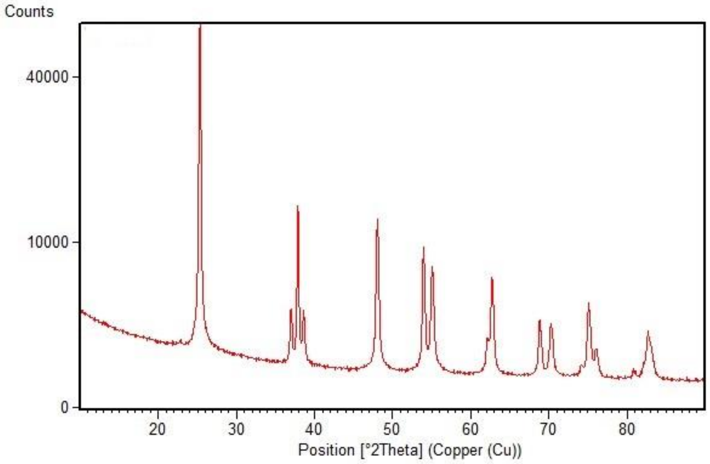

Figure S79. X-ray diffraction pattern of the TiO_2 HT-AspectRatio_02 material.

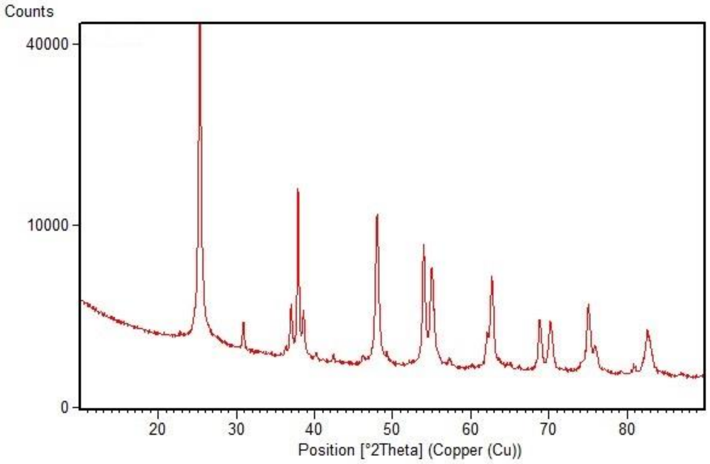

Figure S80. X-ray diffraction pattern of the TiO_2 HT-AspectRatio_03 material. Trace of brookite phase is visible.

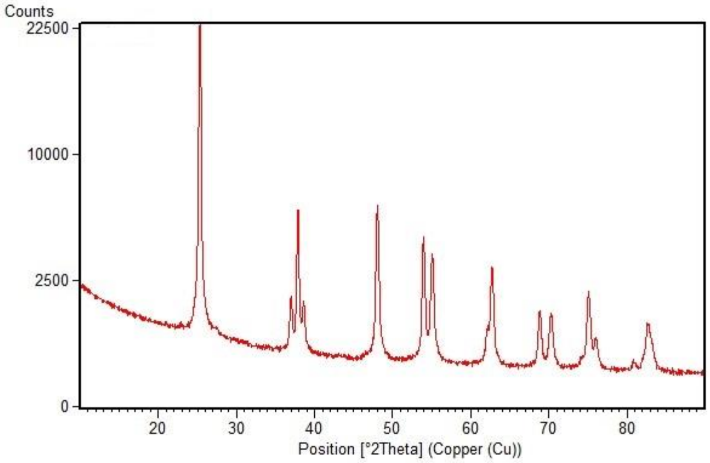

Figure S81. X-ray diffraction pattern of the last TiO_2 predicted material.

9. Dynamic Light Scattering

The analysis of the nanoparticles dimensions can be carried out with dynamic light scattering (DLS) as a fast alternative method to electron microscopy. This technique measures the translational diffusion coefficient and, through to the Stokes-Einstein equation, the hydrodynamic radius. Therefore, electron microscopy and DLS results cannot be directly compared, but the use of the Perrin formula (see section Method) enables the comparison. Table S4 (see SI) reports the hydrodynamic radii calculated with the Perrin formula with major and minor axes of ellipses fitted to the NP's boundary and experimentally determined by means of DLS. The dynamic light scattering data were obtained by analyzing the raw suspensions diluted using NH_3 200 mM as a dispersant solvent and after sonication for 10-30 minutes in an ultrasound bath and in a closed vial to avoid NH_3 evaporation (suggested 95 W, 37 kHz). Suggested concentration is 50 mg/L. The DLS analysis were carried out exclusively on stable colloids in order to avoid a change in the signal during time due to the agglomeration. The reported dimensions were obtained through the fit of the (decay times) distribution function to the integral equation relating the field correlation function and the said distribution function using a constrained regularization method (CONTIN DP algorithm) developed by Provencher (Computer Physics Communication, 27, 229-242 (1982)). The number distribution function is then obtained. The measurements and their polydispersity (as standard deviation of the relevant peak mode) are reported in Table S4 together with the shape and size parameters obtained by the microscopic analysis; the number distribution function is reported in the following figures (Figure S82, Figure S83, Figure S84, Figure S85). Table S3 and Table S4 highlight the effects of precursor and shape controller concentrations, temperature and pH on the final TiO_2 nanoparticles morphology. Within the parameter ranges considered here, we observed a variation of the hydrodynamic radius (DLS) between 2 and 21 nm (seeTable S4). Concurrently, the variation of the aspect ratio was between 1.3 and 5.5 (see Table S3). The second mode observed in the DLS distributions of samples synthesized at pH < 9 and > 10.5 is due to the formation of agglomerates not completely removed by sonication. The agreement between the R_H determined by evaluation of the microscopic data (major and minor axes of the ellipses fitted to the NP boundaries) and the R_H of the first mode (for bimodal distribution) of the DLS size distribution is generally fairly good, within 2 nm or 10%. The exceptions are HT02, HT07 and HT08, for which aggregation/agglomeration phenomena occur probably due to the strong elongation of these nanoparticles.

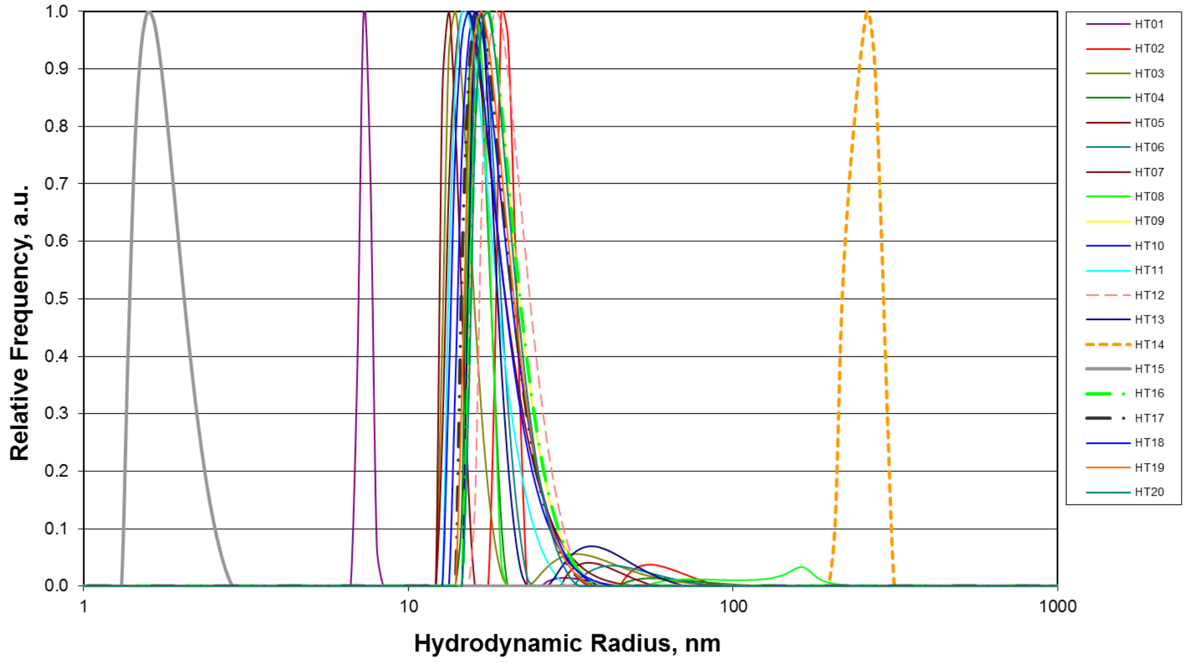

Figure S82. Hydrodynamic Radius distributions for all the materials of the experimental design

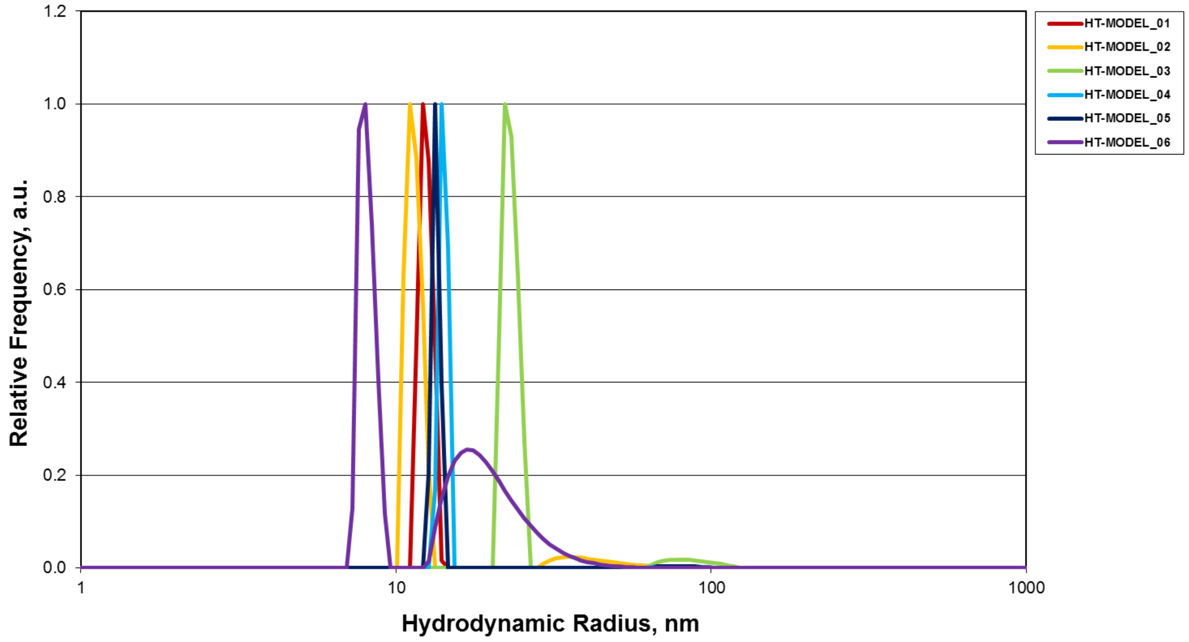

Figure S83. Hydrodynamic Radius distributions for the first validation experiments

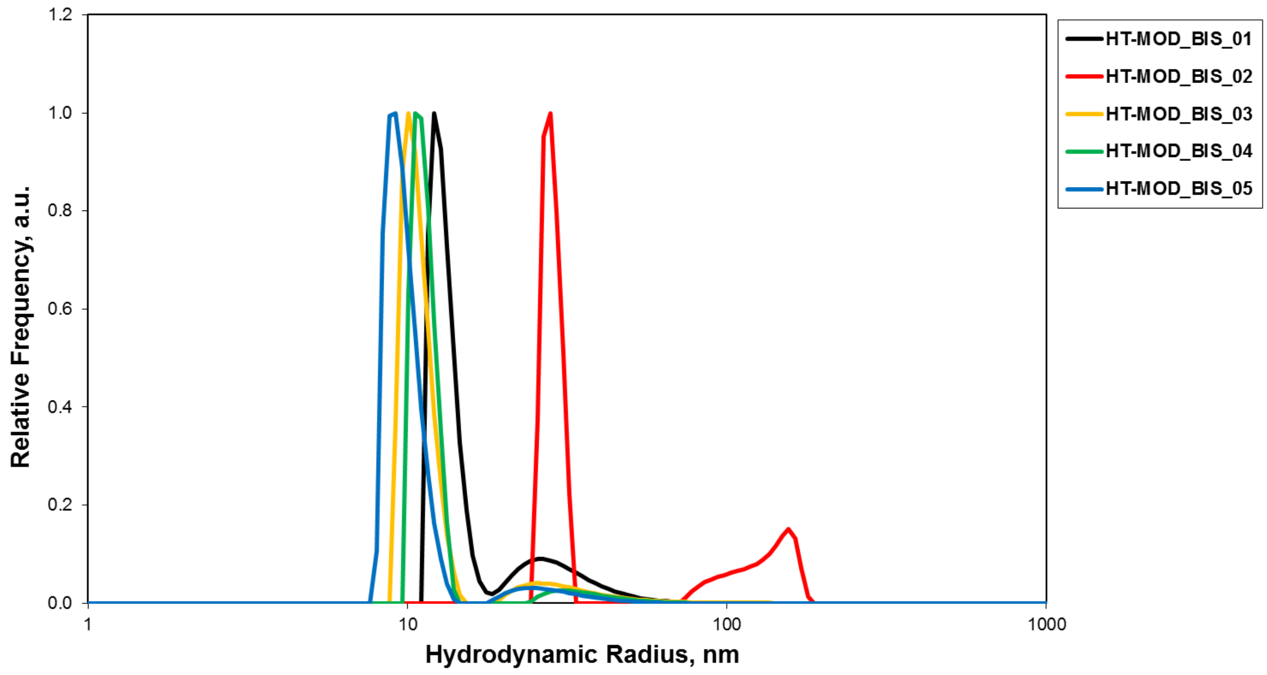

Figure S84. Hydrodynamic Radius distributions for the second validation experiments

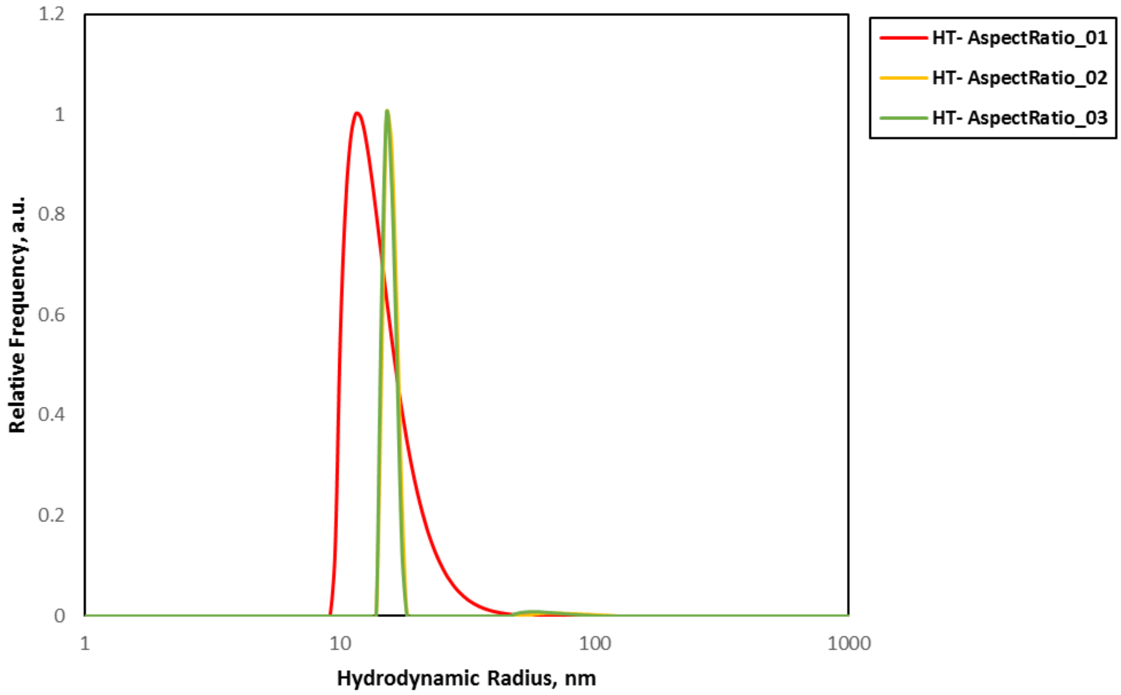

Figure S85. Hydrodynamic Radius distributions for the imposed aspect ratio experiments

10. Second Degree Polynomial Models

The polynomial models were built using coded values (x_1, x_2, x_3, x_4) of experimental conditions:
x_1 = $Ti(Teoa)_2$ concentration, x_2 = $TeoaH_3$ concentration, x_3= pH, x_4 = temperature. Y_1 stands for hydrodynamic radius, Y_2 is the polydispersity measured from DLS data and Y_3 the aspect ratio.
The regressions were performed in Mathcad 14 software using Regress function for a complete second degree polynomial with 4 independent variables. The global measure of the correlation capacity of the models was put into evidence by the coefficient of determination, R^2.

<u>Initial models (20 data fractional Box Wilson program)</u>

$$Y_1(x_1,x_2,x_3,x_4) = 18.436671 + 0.511921x_1 + 1.114592x_2 + 2.14623x_3 + 2.643956x_4 - 0.125x_1x_2 - 0.75x_1x_3 - x_1x_4 - x_2x_3 - 0.75x_2x_4 - 0.125x_3x_4 + 0.618335x_1^2 + 0.40324x_2^2 - 0.887329x_3^2 - 3.038278x_4^2$$

R^2 = 0.782

$$Y_2(x_1,x_2,x_3,x_4) = 19.610424 + 1.757455x_1 - 1.680656x_2 - 0.3835483x_3 + 0.975868x_4 + 0.5631251x_1x_2 - 0.865625x_1x_3 - 1.668125x_1x_4 - 1.668125x_2x_3 - 0.865625x_2x_4 + 0.5631253x_3x_4 - 0.894754x_1^2 - 1.88204x_2^2 - 5.203105x_3^2 - 2.148757x_4^2$$

R^2 = 0.766

$$Y_3(x_1,x_2,x_3,x_4) = 1.43082 + 0.08296x_1 + 0.065553x_2 + 1.132577x_3 - 0.869457x_4 - 0.386875x_1x_2 + 0.009375x_1x_3 + 0.013125x_1x_4 + 0.013125x_2x_3 + 0.009375x_2x_4 - 0.386875x_3x_4 + 0.047899x_1^2 - 0.018781x_2^2 + 0.686731x_3^2 + 0.523259x_4^2$$

R^2 = 0.998

<u>Models with 26 data</u>

$$Y_1(x_1,x_2,x_3,x_4) = 18.311893 + 0.407102x_1 + 1.310852x_2 + 1.913921x_3 + 2.726356x_4 - 0.706556x_1x_2 - 1.330618x_1x_3 - 1.873684x_1x_4 - 0.108507x_2x_3 + 0.051493x_2x_4 + 0.547527x_3x_4 + 0.732221x_1^2 + 0.773031x_2^2 - 1.643796x_3^2 - 2.74701x_4^2$$

R^2 = 0.832

$$Y_2(x_1,x_2,x_3,x_4) = 19.45097 + 1.812474x_1 + 1.199042x_2 - 0.715563x_3 - 1.435496x_4 - 2.835291x_1x_2 + 0.134572x_1x_3 + 2.128591x_1x_4 - 5.337425x_2x_3 - 2.03686x_2x_4 + 3.923907x_3x_4 - 0.652813x_1^2 - 2.486142x_2^2 - 4.448121x_3^2 - 2.465596x_4^2$$

R^2 = 0.795

$$Y_3(x_1,x_2,x_3,x_4) = 1.416942 + 0.080658x_1 + 0.074693x_2 + 1.13782x_3 - 0.816469x_4 - 0.305752x_1x_2 + 0.076544x_1x_3 - 0.055872x_1x_4 + 0.082533x_2x_3 - 0.05335x_2x_4 - 0.461919x_3x_4 + 0.055628x_1^2 + 0.025852x_2^2 + 0.678453x_3^2 + 0.49161x_4^2$$

R^2 = 0.998

Final models with 34 data

$$Y_1(x_1,x_2,x_3,x_4) = 19.36549 - 0.2797x_1 + 1.56885x_2 + 3.5447x_3 + 1.82225x_4 - 1.1978x_1x_2 - 1.66594x_1x_3 - 1.62873x_1x_4 - 0.02003x_2x_3 - 0.001268x_2x_4 - 0.35086x_3x_4 + 0.3914x_1^2 + 0.52265x_2^2 - 0.81701x_3^2 - 2.74921x_4^2$$

R^2 = 0.769

$$Y_2(x_1,x_2,x_3,x_4) = 19.6114239 + 1.0313718x_1 + 1.48527x_2 + 1.7991534x_3 - 4.1983899x_4 + 1.4263262x_1x_2 - 0.4279443x_1x_3 - 1.3865203x_1x_4 - 1.051601x_2x_3 - 2.06380x_2x_4 - 2.476674x_3x_4 - 0.4497319x_1^2 - 1.8040123x_2^2 - 3.8699325x_3^2 - 2.6148x_4^2$$

R^2 = 0.764

$$Y_3(x_1,x_2,x_3,x_4) = 1.65475 - 0.0010507x_1 + 0.081061x_2 + 1.24559795x_3 - 0.9446382x_4 + 0.0251679x_1x_2 - 0.0087112x_1x_3 - 0.0151489x_1x_4 + 0.0948893x_2x_3 - 0.0476591x_2x_4 - 0.7316x_3x_4 - 0.0040507x_1^2 - 0.0546547x_2^2 + 0.6761123x_3^2 + 0.4905399x_4^2$$

R^2 = 0.967

Models for the length of the NPs along the c-axis (34 data) and its Standard Deviation

$$Length(x_1,x_2,x_3,x_4) = 47.576759 - 2.490607x_1 + 2.93623x_2 + 28.02371x_3 - 8.31341x_4 + 1.217006x_1x_2 - 5.35429x_1x_3 - 2.144865x_1x_4 + 1.790157x_2x_3 + 1.620863x_2x_4 - 10.017107x_3x_4 - 0.87459x_1^2 - 0.616409x_2^2 + 5.204391x_3^2 + 7.297355x_4^2$$

R^2 = 0.802

$$Standard\ Deviation\ of\ length(x_1,x_2,x_3,x_4) = 9.9407 - 0.1593x_1 + 1.6765x_2 + 11.5121x_3 - 2.7567x_4 + 1.3412x_1x_2 - 1.9655x_1x_3 + 0.9093x_1x_4 + 1.4837x_2x_3 - 0.5613x_2x_4 - 2.9441x_3x_4 + 1.9136x_1^2 + 1.0102x_2^2 + 5.5316x_3^2 + 3.1849x_4^2$$

R^2 = 0.834

11. Artificial Neural Networks (ANN) optimization

Basic considerations on ANN formulation

ANNs belong to the stochastic modeling approach aiming to reflect the influence of independent variable (inputs) upon measured results (outputs). ANN models are apparently similar to polynomial regression models, but, in fact they differ from several points of view. ANNs are a sort of “grey box”, being designed to mimic the information processing and knowledge acquisition methods of the human brain.

The problems handled by neural networks can be quite varied. On the most general level the former can be divided into four basic types:

- association (auto or hetero),
- classification,
- transformation (different representation),
- modelling.

In the present study, ANNs were used for modelling. ANN model does not require knowledge of the mathematical equations mimicking the process: the nonlinearity of a single unit transformation and a sufficiently large number of variable parameters (weights) ensure enough "freedom" to adapt the ANN to any relation between input and output data.

The ANN mimics a surprising number of the brain’s characteristics:

- learn from experience
- generalize from previous examples
- abstract essential characteristics from input containing scattered irrelevant data, as any self-organizing system.

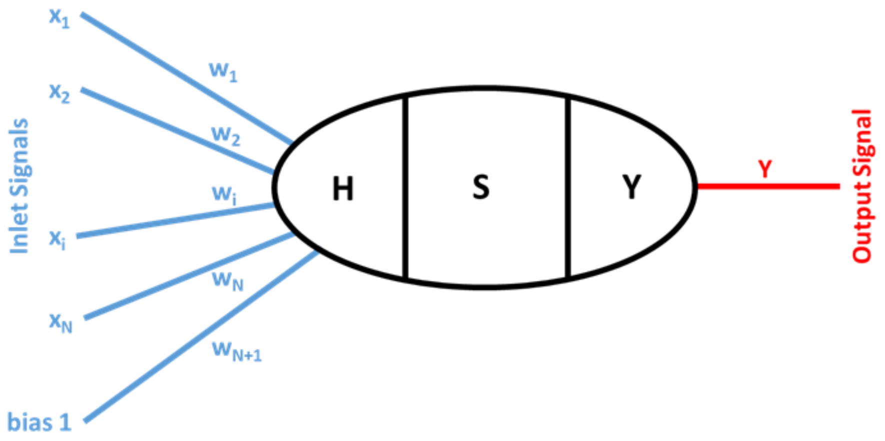

Figure S86. The synthetic (artificial) neuron. x_i values are the component of the input vector, each with its weight w_i. S is the activation function, which processes the input H to give the output vector Y

ANN performs better than the classical polynomial regression, because of its generalization capacity, providing a correct answer to a question outside the learning set. The ANN generalization relies on its capacity of finding out the hidden rules that govern the process,

even if, at this time, it cannot be mathematically expressed. This quality becomes effective when the data used for training and testing are in large amount.
The information is processed in an ANN by the neurons. The inlet information is abstracted into a vector, *X*:

$$X = (x_1, \dots, x_N, 1)$$

The importance of each "dendrite information" is abstracted into the weights, *W*:

$$W = (w_1, \dots, w_{N+1})$$

The processed signal is H, which will feed the activation function:

$$H = h(x_1 w_1, \dots, x_N w_N, w_{N+1})$$

The most common inlet global function, *h*, is addition:

$$h = \sum_{i=1}^{N+1} x_i w_i \quad with\ x_{N+1} = 1$$

The power of the synthetic neuron is given by its activation function, f, applied upon the processed signal, H.

$$S = f(H)$$

The most used activation functions, f, are:

$$f(H) = \begin{cases} sign(H-\sigma) = \begin{cases} 1, H-\sigma \geq 0 \\ -1, H-\sigma < 0 \end{cases} \\ ramp(H,\alpha,\sigma) = max\{0, min[1, \alpha \cdot (H-\sigma)]\} \\ sf(H,\alpha,\sigma) = \dfrac{1}{1+\dfrac{\exp(-\alpha \cdot H)}{\sigma}} \end{cases}$$

The outlet signal, Y, results processing further the outcome of the activation function, S.

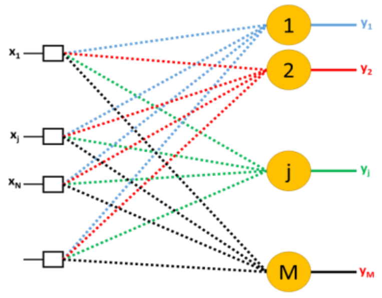

Figure S 87. The non-active (input) and active layers

$$Y = g(S)$$

Generally, the outlet function, g, is the identity:

$$g(S) = S$$

In our study the sigmoid activation function was used.

The learning algorithm, through which the weights are modified so the output of the neuron should match the reality.

The first and last layers are for input and output, respectively, while the others are the hidden layers. The network is said to be fully connected when any node in a given layer is connected to all the nodes in the adjacent layers. The learning algorithm is back-propagating. Matching the ANN output to the real world passes through the attached neuron weights, which modify until the learning criterion is fulfilled, i.e. the output matches reality.

In the present study, three distinct modelling ANNs were built to reflect the influence of working parameters upon the characteristics of the bipyramidal anatase synthetized. All three networks have the same architecture: four input neurons, for the four independent variables – $Ti(TeoaH)_2$ concentration (mM), added $TeoaH_3$ concentration (mM), pH and temperature, three neurons in the hidden layer – to ensure learning capability, without increasing the number of ANN weights too much, and one output neuron, for the dependent variables to be thought – the hydrodynamic radius (Y1), the polydispersity (Y2) and Aspect Ratio (Y3), respectively.

The ANN were trained and tested in the frame of Matlab R2015a software. The data sets were used 70-80% for training, and 30-20% for validation and testing to prevent overfitting and test the prediction capability.The implemented training algorithm, "trainlm" is used. It is the fastest backpropagation algorithm in the Matlab toolbox and it updates the weight and bias values according to Levenberg-Marquardt optimization.

The other hyperparameters are:

- Number of epach: max 1000;
- Maximum validation failures 6;
- Minimum performance gradient 1e-7;
- Initial Mu 0.001;
- Mu increasing factor: 10;
- Mu decreasing factor: 0.1;
- maximum Mu: 1e10;

Simulation of the Results before Validation

As for the results presented in the main text, here we reported simulations obtained from the ANN model before the validation and optimization (i.e. with the first 20 experiments results only) at different ratios of Ti(TeoaH)$_2$ and TeoaH$_3$.

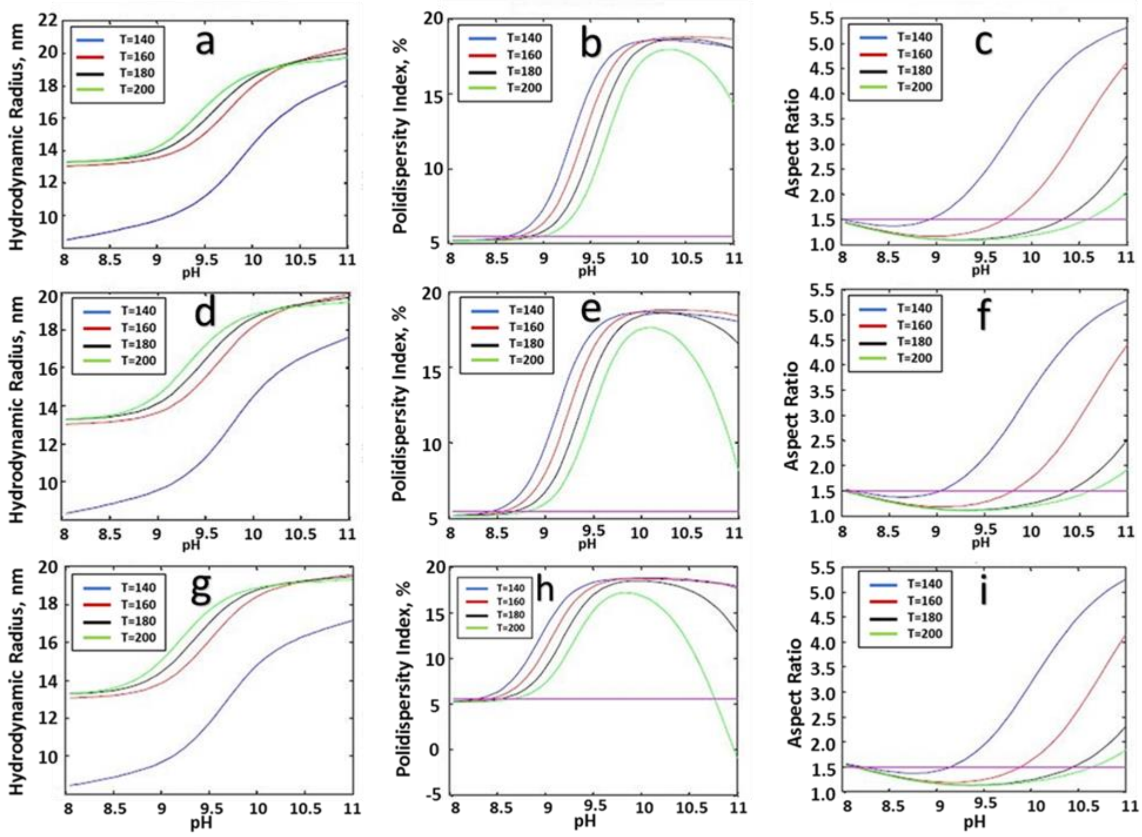

Figure S88. Variation of main particle characteristics (hydrodynamic radius: left column, polydispersity index: middle column, aspect ratio: right column) at low initial Ti(TeoaH)$_2$ 35 mM: TeoaH$_3$ 15mM (a,b,c), :TeoaH$_3$ 25mM (d,e,f) and :TeoaH$_3$ 35 mM (g,h,i).

Figure S88 presents the influence of working conditions of synthesis upon the characteristics of both the final particle hydrodynamic radius distribution and the aspect ratio. As pH has the greatest influence upon the final NPs characteristics, the variation of the hydrodynamic radius, the polydispersity and the aspect ratio are represented as functions of pH at different temperatures. As for initial Ti(TeoaH)$_2$ and TeoaH$_3$ concentrations, several conditions were considered: high and low Ti(TeoaH)$_2$ concentration and various Ti(TeoaH)$_2$/TeoaH$_3$ ratios. As Figure S88(a,b,c) shows, the influence of temperature is more important for the aspect ratio, being higher as pH increases. The polydispersity is more affected by the increase of pH; for pH around 9.5, the temperature decrease may drastically affect the polydispersity index (the polydispersity is 8% at pH = 9.5 and T = 200°C, while at pH = 9.5 and T = 140°C the former

reaches 15%). For the aspect ratio, at each temperature two working points are possible for the prescribed value of 1.5, but only those at very low pH ensure also a polydispersity of 5% as required for metrological applications. The hydrodynamic radius is strongly influenced by both pH and temperature. Small NPs, under 15 nm, can be obtained at pH ≤ 9. Nanoparticles smaller than 10 nm can only be obtained at low temperatures, around 140°C, again at pH < 9. Figure S88(d,e,f) represents the influence of the working parameters (pH and temperature) keeping the same $Ti(TeoaH)_2$ concentration as in Figure S88(a,b,c), but at a higher initial $TeoaH_3$ concentration. The hydrodynamic radius, polydispersity index and the aspect ratio are not significantly affected by the increase of added $TeoaH_3$ at low and medium temperature levels. At high temperatures (T = 200°C), some working points at high pH (about 11) can be identified if a polydispersity index of 8-9 % and R_H = 18 nm may be accepted. Figure S88(g,h,i) shows that when the initial $TeoaH_3$ concentration is very high compared to the $Ti(TeoaH)_2$ load, the multiplicity of favourable working conditions appears at temperature of 200°C – same polydispersity and aspect ratio could be obtained at low pH, around 8.5, and at high pH, around 10.7. Very small particle hydrodynamic radius values, below 10 nm can be obtained only at low temperature regardless the $Ti(TeoaH)_2$ and $TeoaH_3$ ratios.

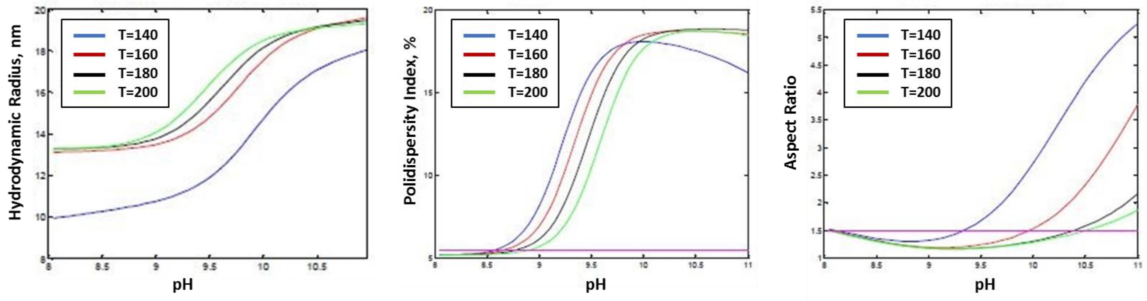

Figure S89. Variation of main particle characteristics at $Ti(TeoaH)_2$ 65mM and $TeoaH_3$ 15mM

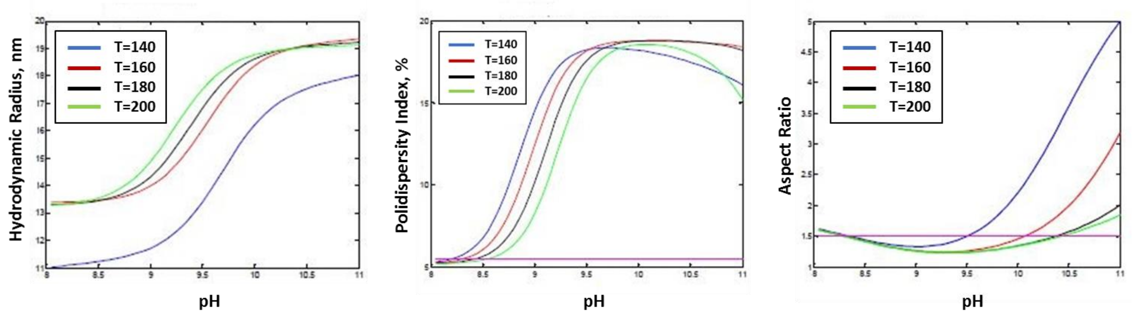

Figure S90. Variation of main particle characteristics at $Ti(TeoaH)_2$ 65mM and $TeoaH_3$ 35mM

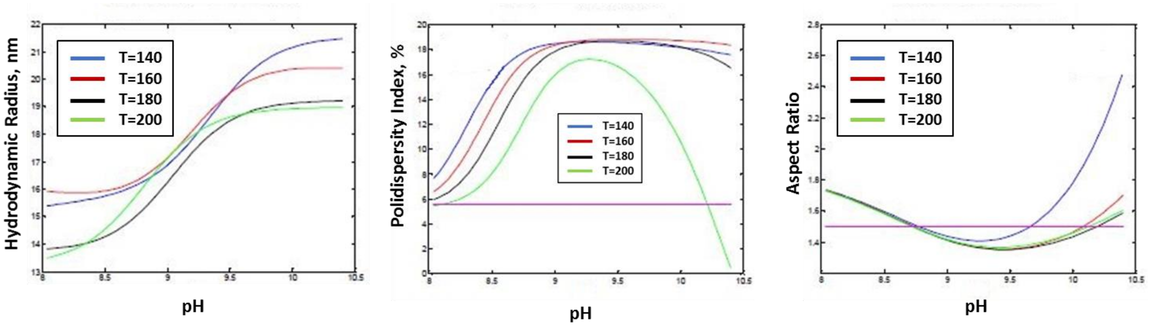

Figure S91. Variation of main particle characteristics at Ti(TeoaH)$_2$ 65mM and TeoaH$_3$ 65mM

As Figure S89-Figure S91 show, at higher Ti(TeoaH)$_2$ load, and the initial TeoaH$_3$ in low or moderate concentration compared to that of Ti(TeoaH)$_2$, the influence of pH and temperature are quite similar with the case of Ti(TeoaH)$_2$ at concentration of 35 mM, with the additional consideration that particles with sizes around 10 nm are possible only at low initial TeoaH$_3$ concentration. When the latter equals the Ti(TeoaH)$_2$'s, the general observation that low temperature leads to smaller particle formation seems no longer to hold (Figure S91). As for the aspect ratio, the required value of 1.5 is reached at pH = 8.7 where the polydispersity is above 14% at all temperatures. So, adding too much initial amount of TeoaH$_3$ is not in favor of the prescribed particles characteristics.

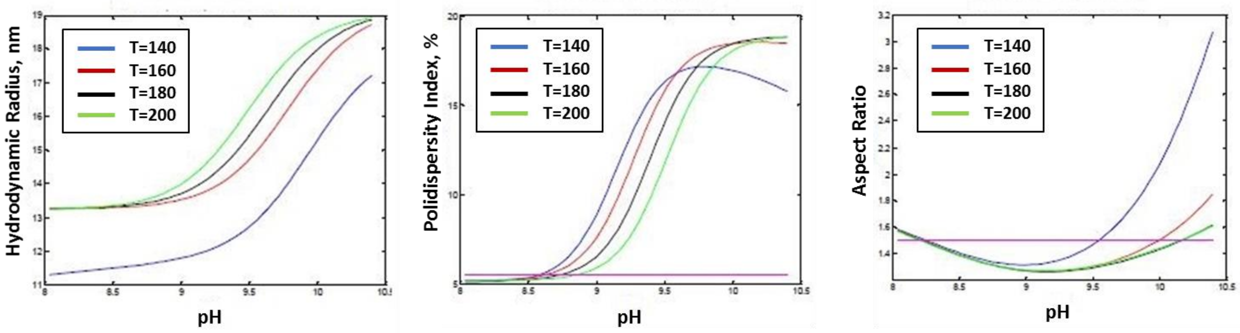

Figure S92. Variation of main particle characteristics at Ti(TeoaH)$_2$ 90mM and TeoaH$_3$ 15mM

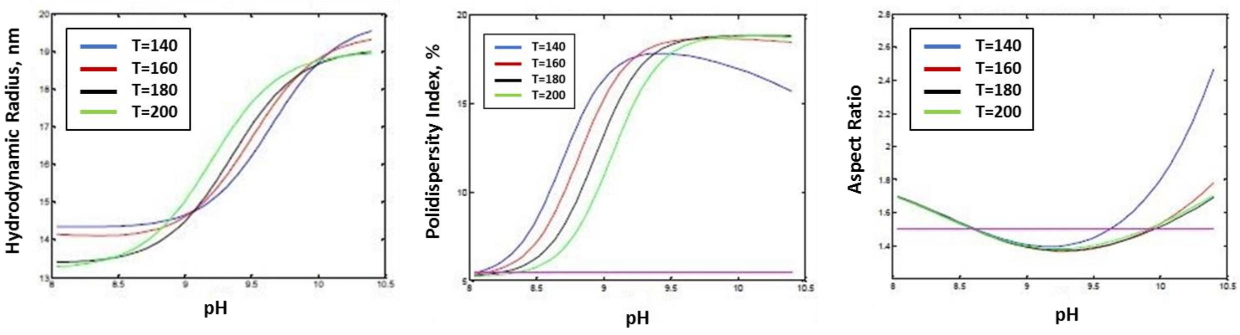

Figure S93. Variation of main particle characteristics at Ti(TeoaH)$_2$ 90mM and TeoaH$_3$ 40mM

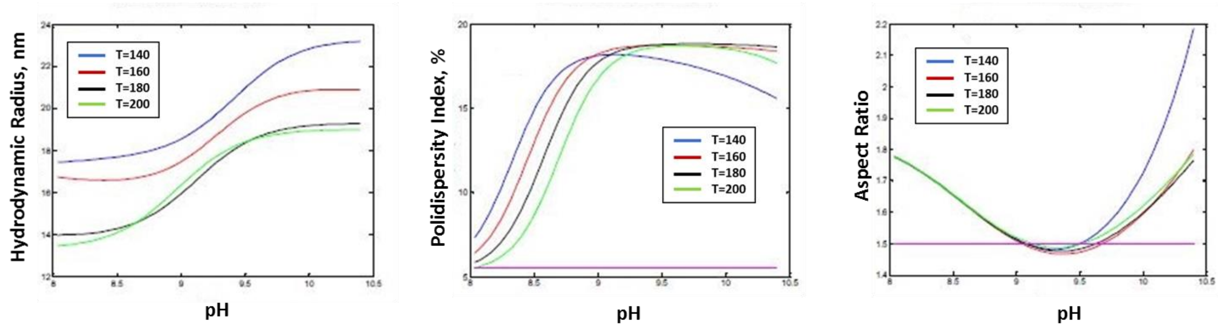

Figure S94. Variation of main particle characteristics at Ti(TeoaH)$_2$ 90mM and TeoaH$_3$ 60mM

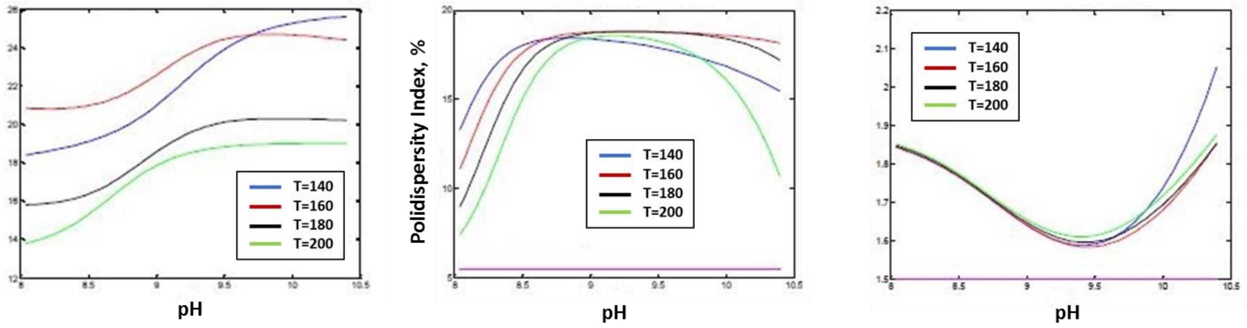

Figure S95. Variation of main particle characteristics at Ti(TeoaH)$_2$ 90mM and TeoaH3 80mM

According to Figure S92-Figure S95, at very high Ti(TeoaH)$_2$ concentrations, good working conditions may be identified at lower initial added TeoaH$_3$ concentrations. For a pH around 8.5, the desired polydispersity index and aspect ratio can be reached in a large range of temperatures. If the initial TeoaH$_3$ concentration is over 60mM, there are no working conditions to ensure the required particle characteristics (Figure S94 and Figure S95).

Analysing all the results, we can summarize:

i. When pH ≥9.5, both polydispersity and aspect ratio present a higher sensitivity to temperature variations;

ii. pH and temperature have, in this order, the greatest influence upon polydispersity and aspect ratio. For certain initial conditions (high temperatures and TeoaH$_3$/Ti(TeoaH)$_2$ ratios, as well), the aspect ratio could present multiplicities, which could happen to polydispersity too, but seldom, though;

iii. At ≥ 65mM Ti(TeoaH)$_2$ (see Figure S68-S70 in SI) the particle hydrodynamic radius values are around 10 nm at 140°C only. At 90 mM Ti(TeoaH)$_2$ (see Figure S71-S74 in SI), if the initial TeoaH$_3$ concentration is larger than 40 mM, the particle hydrodynamic radius values will be greater than 14 nm end even bigger at higher pH values. Nonetheless, at initial TeoaH$_3$ concentration ≥ 80 mM, none of the desired characteristics could be obtained;

iv. When working at 200°C and pH ≥ 10, the initial TeoaH$_3$ concentration determines multiplicity for polydispersity;

The recommended working domain for metrological purposes, ensuring the completion of restrictions for all three parameters (the hydrodynamic radius, the polydispersity and the aspect ratio) is between 8 and 9 for pH, while for temperature is between 160°C and 200°C; as the initial concentration of added $TeoaH_3$ increases, the process becomes more sensitive to temperature.

Validation Experiments for the ANN models

The validation of the mathematical model was realized by performing new experiments in the range of interest for the operating parameters. The experimental outcomes for the obtained TiO_2 NPs were compared with predicted ones in Table S6.

Table S5. Synthesis parameters for the 6 validation experiments

Experiment Name	Z_1, mM	Z_2, mM	Z_3, pH	Z_4, °C
HT- MODEL_01	85	9	8.3	163
HT- MODEL_02	82	56	8.0	202
HT- MODEL_03	66	80	10.0	200
HT- MODEL_04	65	20	9.2	215
HT- MODEL_05	72	40	8.2	208
HT- MODEL_06	33	14	8.8	138

Table S6. Product predicted and measured characteristics in some test points (based on T-SEM micrographs and R_H) for ANN and Polynomial Models with relative errors.

Experiment Name	Product characteristics								
	Rh, nm			polydispersity from DLS, %			Aspect Ratio		
	ANN	Polynomial	Experimental	ANN	Polynomial	Experimental	ANN	Polynomial	Experimental
HT-MODEL_01	13	12	13	5.2	5.2	4.4	1.4	1.4	1.4
HT-MODEL_02	13	15	11	2.3	6.3	5.6	1.7	1.5	1.5
HT-MODEL_03	19	20	24	6.0	14	5.3	1.5	1.3	1.3
HT-MODEL_04	16	15	14	5.6	9.8	2.9	1.2	1	1.4
HT-MODEL_05	13	13	12	5.3	5.5	3.1	1.6	1.5	1.4
HT-MODEL_06	8	6	7	5.8	5	6.4	1.5	1.7	1.5
Mean Relative Error	11 %	12 %		31 %	38 %		8%	7 %	

The ANN models seems to have a higher prediction ability mainly for the polydispersity. The models have been refined and mean relative error (%) reported in Table 1 of the main text.

Optimized Conditions for the Refined ANN Models

The search for best operating conditions for given product characteristics was achieved by formulation of an optimization problem. The definition of the goal was realized in several ways:

a) The objective function selected was the minimization of the hydrodynamic radius (Y1) with the restrictions: low polydispersity (Y2 < 5) and shape factor close to 1.5 (1.4 < Y3 < 1.55). The values of Y1, Y2, Y3 at each step of the GA calculations was realized by using the neural models. The results obtained showed that several suboptimal (local) optimum can be defined corresponding more or less to the product characteristics considered:
   - Z1, $Ti(TeoaH)_2$ concentration = 65.5 mM;
   - Z2, added $TeoaH_3$ = 14.3 mM;
   - Z3, pH = 8.66;
   - Z4, Temperature = 206.3°C;

   The minimum value for Y1 is 9.6 nm and the restrictions are respected: Y2 = 3.28%, Y3 = 1.4.

b) Solving the problem by using a composite objective function to include all three conditions (minimum Y1, low Y2 and Y3 close to 1.5) and avoid restrictions formulation. This function was defined as:

$$F_{ob} = Y1 + Y2 + |(Y3 - 1.5) * 100|$$

   the multiplication with 100 of the third term contribution is meant to provide the same order of magnitude to all terms and increase the importance of the aspect ratio.
   The minimization process lead to the following results:
   - for the range of high $Ti(TeoaH)_2$ load the best solution found is: **Z1 = 75 mM, Z2 = 51 mM, pH = 8.3, T = 203°C**. The minimum value of hydrodynamic radius that corresponds to these working condition is Y1 = 11.5 nm, the lowest value of polydispersity Y2 = 4.3 % and Y3 = 1.49.
   - Searching over the whole concentration range the solution is: **Z1 = 46 mM; Z2 = 21 mM; pH = 8.2, T = 140°C**. The corresponding product characteristics are: Y1 = 9.58 nm; Y2 = 3.5 % and Y3 = 1.45.

c) If predefined parameters are meant for all three product characteristics (for instance Y1=11 nm, Y2=3.5%, Y3=1.5), then the objective function may be formulating like:

$$F_{ob} = |(Y1 - 11) + (Y2 - 3.5) + (Y3 - 1.5) * 100|$$

   The minimization by GA of this objective function lead to the solution: **Z1 = 64 mM; Z2 = 15.5 mM; pH = 9.2, T = 200.4°C**. The corresponding product characteristics are: Y1 = 10.9 nm; Y2 = 3.4 % and Y3 = 1.48.
   For larger hydrodynamic radius, the objective function is:

$$F_{ob} = |(Y1 - 14) + (Y2 - 4.5) + (Y3 - 1.5) * 100|$$

   Searching in the whole range of operating variables, the optimum obtained is: **Z1 = 65.6 mM; Z2 = 37.1 mM; pH = 8.93, T = 209.4°C**. The corresponding product characteristics are: Y1 = 16.0 nm; Y2 = 4.5 % and Y3 = 1.42.

Reverse Engineering ANN models

Five additional validation experiments for the reverse engineering models were carried out together with three imposed aspect ratio experiments.

Table S7. Synthesis parameters of the validation experiments for the refined model and experiments with imposed aspect ratio

Experiment Name	Z_1, mM	Z_2, mM	Z_3, pH	Z_4, °C
HT- MOD_BIS_01	65	14	8.3	200
HT- MOD_BIS_02	30	60	10.5	200
HT- MOD_BIS_03	40	64	8.3	140
HT- MOD_BIS_04	30	15	8.6	200
HT- MOD_BIS_05	90	15	8.6	140
HT- AspectRatio_01	100	15	10.5	145
HT- AspectRatio_02	54	65	11.0	163
HT- AspectRatio_03	56	37	11.3	147

Table S8. Validation experiments (for the optimized models) and experiments with imposed aspect ratio predicted (by ANN and Polynomial Models with relative errors.) and measured characteristics

	Product characteristics								
Experiment Name	Rh, nm			polydispersity from DLS, %			Aspect Ratio		
	ANN	Polynomial	Experimental	ANN	Polynomial	Experimental	ANN	Polynomial	Experimental
HT- MOD_BIS_01	13	12	14	4	-3.4	7	1.4	1.3	1.4
HT- MOD_BIS_02	23	23	28	11	10.7	7	1.7	1.8	1.3
HT- MOD_BIS_03	8	9	11	26	25	9	1.9	1.9	1.5
HT- MOD_BIS_04	14	13	12	6	-5.8	8	1	0.8	1.4
HT- MOD_BIS_05	12	13	11	9	8.9	9	1.9	2	1.6
HT- AspectRatio_01	18	17	20	16	16	20	3.3	3.4	2.3
HT- AspectRatio_02	20	21	24	15	11	21	3.2	3.2	3.1
HT- AspectRatio_03	17	15	28	14	11	28	4.2	4.3	6.3
Mean Deviation from Exp	3.6	3.7		6.3	9.4		0.58	0.65	
Mean Relative Error	17 %	14 %		34 %	69 %		19 %	23 %	

The data shown in Table S8 highlight that there is a certain discrepancy between the experimental and the predicted results, especially for the materials with imposed aspect ratio (last three materials). Therefore, the models have been refined and mean relative error (%) reported in Table 2 of the main text.

Final Refined Model with the Reverse Engineering Experiments

Table S9 shows the experimental characteristics of NPs and the computed values from the last ANNs and the polynomial models.

Table S9. Experimental and computed values for product characteristic using the refined ANN models and the polynomial models

Experiment Name	Y_1, nm			Y_2, %			Y_3, Aspect Ratio		
	ANN	Polynomial	Experimental	ANN	Polynomial	Experimental	ANN	Polynomial	Experimental
HT01	6	5	7	4	5	4	1,6	1,6	1.5
HT02	20	23	20	6	10	5	1,9	2,1	2
HT03	14	18	15	3	6	9	1,4	1,1	1.4
HT04	25	22	22	18	18	18	5,5	5,7	5.5
HT05	12	15	14	5	6	5	1,4	1,2	1.4
HT06	20	18	20	19	18	15	6,1	5,4	5.5
HT07	14	14	18	19	20	25	1,5	1,7	1.7
HT08	18	19	17	6	5	6	2,3	2,4	2.3
HT09	21	21	20	17	17	18	1,4	1,5	1.4
HT10	18	20	19	19	21	19	1,5	1,5	1.8
HT11	17	18	17	16	13	17	1,5	1,3	1.3
HT12	21	23	21	19	18	16	1,8	1,6	1.5
HT13	14	12	13	4	8	5	1,4	1,2	1.4
HT14*	19	23	19	13	13	13	4,8	5,0	4.7
HT15	4	10	2	19	20	14	4,0	4,1	4
HT16	20	16	20	16	7	17	1,4	1,3	1.4
HT17	19	19	20	19	20	17	1,6	1,6	1.5
HT18	20	19	17	19	20	23	1,6	1,6	1.4
HT19	20	19	20	19	20	17	1,6	1,6	1.4
HT20	19	19	18	19	20	17	1,6	1,6	1.4
HT-MODEL_01	14	14	13	4	5	4	1,4	1,0	1.4
HT-MODEL_02	13	13	11	5	6	6	1,4	1,8	1.5
HT-MODEL_03	23	22	24	5	9	5	1,5	1,0	1.3
HT-MODEL_04	15	14	14	4	8	3	1,4	1,3	1.4

HT-MODEL_05	12	12	12	4	5	3	1,4	1,9	1.4
HT-MODEL_06	7	6	7	4	6	6	1,6	1,6	1.5
HT-MOD_BIS_01	14	12	14	4	5	7	1,4	1,5	1.4
HT-MOD_BIS_02	26	25	28	6	10	7	1,6	1,4	1.3
HT-MOD_BIS_03	12	10	11	9	9	9	1,4	1,4	1.5
HT-MOD_BIS_04	14	13	12	6	8	8	1,4	1,3	1.4
HT-MOD_BIS_05	14	12	11	9	8	9	1,4	1,5	1.6
HT-AspectRatio_01	11	18	20	19	18	20	2,3	3,1	2.3
HT-AspectRatio_02	25	24	24	21	18	21	3,1	3,3	3.1
HT-AspectRatio_03	25	20	28	27	20	28	6,2	4,9	6.3
Mean Relative Error, %	9.1	11.9		13.9	19.5		5.6	10.1	

*Due to the substantial presence of brookite in the sample HT14 and the difficulties in the analysis of the material HT15, the missing data for the aspect ratio were taken in the range of the other experimental values, while the outlier values for Y1 and Y2 in HT14 run were replaced with values in the range of variation in order to avoid a strong model distortion

Models for Length of the Nanoparticles along the c-axis Direction

Table S10 reports the experimentally measured and the calculated (ANNs and polynomial) values of the length of the nanoparticles along the direction of the nanoparticle's c-axis and its standard deviation.

Table S10. Experimental and computed values for product characteristic using the new ANNs and polynomial models.

Experiment Name	Length, nm			Polydispersity, nm		
	ANN	Polynomia l	Experimental	ANN	Polynomial	Experimental
HT01	22	28	23	9	10	7
HT02	83	75	89	32	27	33
HT03	32	35	38	9	6	9
HT04	109	114	108	48	44	47
HT05	32	31	29	9	12	8
HT06	79	97	100	31	31	31
HT07	32	37	27	9	13	10
HT08	79	68	81	37	30	37
HT09	59	54	43	12	15	11
HT10	40	46	52	15	14	15
HT11	39	42	39	11	10	9
HT12	50	51	49	10	15	12
HT13	30	17	29	9	5	9
HT14*	80	103	60	25	40	25
HT15*	79	77	76	18	22	18
HT16	39	52	49	16	13	10
HT17	43	48	47	11	10	10
HT18	43	48	45	11	10	11
HT19	43	48	43	11	10	10
HT20	43	48	44	11	10	11
HT- MODEL_01	32	20	32	9	7	10
HT- MODEL_02	32	34	29	9	12	9
HT- MODEL_03	46	51	45	13	14	13
HT- MODEL_04	32	40	34	10	10	12
HT- MODEL_05	32	36	36	9	11	14
HT- MODEL_06	23	29	24	9	9	7

HT- MOD_BIS_01	32	28	29	9	10	8
HT- MOD_BIS_02	69	64	68	13	17	14
HT- MOD_BIS_03	25	16	23	9	6	8
HT- MOD_BIS_04	32	30	29	9	9	8
HT- MOD_BIS_05	30	31	27	9	7	10
HT- AspectRatio_01	63	67	59	15	17	16
HT- AspectRatio_02	80	81	92	27	27	28
HT- AspectRatio_03	141	98	143	46	33	46
Mean Relative Error, %	8.5	13.5		8.9	18.1	

*Due to the substantial presence of brookite in the sample HT14 and the difficulties in the analysis of the material HT15, the missing data were estimated and taken in the range of the other experimental values

Final Test

To test the final reliability of the models, they were used for the prediction of a synthesis with an intermediate aspect ratio. A very good match between the experimental and calculated characteristics was observed (Table S11).

Table S11. Proposed experiment for testing the final refined models.

Experiment Name	Z_1, mM	Z_2, mM	Z_3, pH	Z_4, °C
Prediction	108	62	11.1	145

Table S12. Experimental and computed values for product characteristic using the new ANNs and polynomial polynomial models.

Experiment Name	Y_1, ANN	Y_1, Polynomial	Y_1, Experimental	Y_2, ANN	Y_2, Polynomial	Y_2, Experimental	Y_3, ANN	Y_3, Polynomial	Y_3, Experimental	Length, ANN	Length, Polynomial	Length, Experimental
Prediction	23	20	26	19	25	8	3.7	4.7	3.7	79	90	71

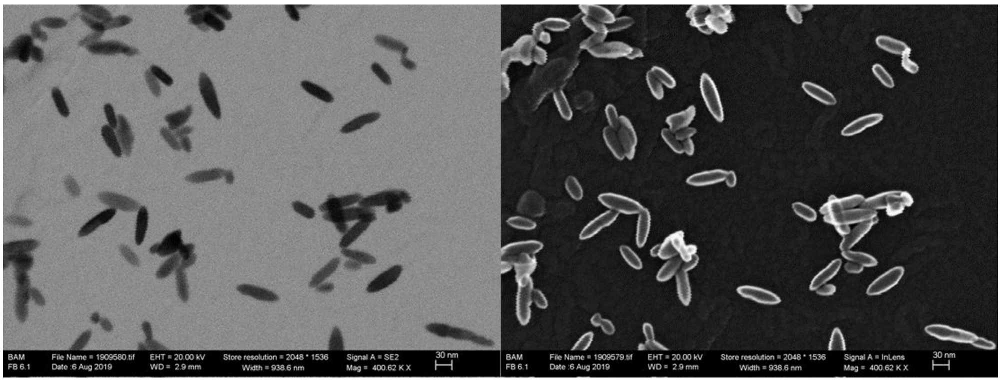

Figure S96. T-SEM and SEM images of sample Prediction

Table S13. Distance between the Models'Prediction and the Experimental Obtained Nanoparticles' Characteristics: hydrodynamic radius, polydispersity and aspect ratio

Material	***Hydrodynamic Radius, nm***		***Polidispersity, %***		***Aspect Ratio***	
	ANN, nm	***Polynomial, nm***	***ANN, %***	***Polynomial, %***	***ANN***	***Polynomial***
HT01	-1.0	-2.0	0.0	1.0	0.1	0.1
HT02	0.0	3.0	1.0	5.0	-0.1	0.1
HT03	-1.0	3.0	-6.0	-3.0	0.0	-0.3
HT04	3.0	0.0	0.0	0.0	0.0	0.2
HT05	-2.0	1.0	0.0	1.0	0.0	-0.2
HT06	0.0	-2.0	4.0	3.0	0.6	-0.1
HT07	-4.0	-4.0	-6.0	-5.0	-0.2	0.0
HT08	1.0	2.0	0.0	-1.0	0.0	0.1
HT09	1.0	1.0	-1.0	-1.0	0.0	0.1
HT10	-1.0	1.0	0.0	2.0	-0.3	-0.3
HT11	0.0	1.0	-1.0	-4.0	0.2	0.0
HT12	0.0	2.0	3.0	2.0	0.3	0.1
HT13	1.0	-1.0	-1.0	3.0	0.0	-0.2
HT14*	0.0	4.0	0.0	0.0	0.1	0.3
HT15*	2.0	8.0	5.0	6.0	0.0	0.1
HT16	0.0	-4.0	-1.0	-10.0	0.0	-0.1
HT17	-1.0	-1.0	2.0	3.0	0.1	0.1
HT18	3.0	2.0	-4.0	-3.0	0.2	0.2
HT19	0.0	-1.0	2.0	3.0	0.2	0.2
HT20	1.0	1.0	2.0	3.0	0.2	0.2
HT- MODEL_01	1.0	1.0	0.0	1.0	0.0	-0.4
HT- MODEL_02	2.0	2.0	-1.0	0.0	-0.1	0.3
HT- MODEL_03	-1.0	-2.0	0.0	4.0	0.2	-0.3
HT- MODEL_04	1.0	0.0	1.0	5.0	0.0	-0.1
HT- MODEL_05	0.0	0.0	1.0	2.0	0.0	0.5
HT- MODEL_06	0.0	-1.0	-2.0	0.0	0.1	0.1
HT-MOD_BIS_01	0.0	-2.0	-3.0	-2.0	0.0	0.1
HT-MOD_BIS_02	-2.0	-3.0	-1.0	3.0	0.3	0.1
HT-MOD_BIS_03	1.0	-1.0	0.0	0.0	-0.1	-0.1
HT-MOD_BIS_04	2.0	1.0	-2.0	0.0	0.0	-0.1
HT-MOD_BIS_05	3.0	1.0	0.0	-1.0	-0.2	-0.1

HT-AspectRatio_01	-9.0	-2.0	-1.0	-2.0	0.0	0.8
HT-AspectRatio_02	1.0	0.0	0.0	-3.0	0.0	0.2
HT-AspectRatio_03	-3.0	-8.0	-1.0	-8.0	-0.1	-1.4

Table S14. Distance between the Models'Prediction and the Experimental Obtained Nanoparticles' Characteristics: length along the c-axis and its standard deviation

Material	***Length along the c-axis***		***Standard Deviation***	
	ANN, nm	***Polynomial, nm***	***ANN, %***	***Polynomial, %***
HT01	1	-5	-2	-3
HT02	6	14	1	6
HT03	6	3	0	3
HT04	-1	-6	-1	3
HT05	-3	-2	-1	-4
HT06	21	3	0	0
HT07	-5	-10	1	-3
HT08	2	13	0	7
HT09	-16	-11	-1	-4
HT10	12	6	0	1
HT11	0	-3	-2	-1
HT12	-1	-2	2	-3
HT13	-1	12	0	4
HT14*	-20	-43	0	-15
HT15*	-3	-1	0	-4
HT16	10	-3	-6	-3
HT17	4	-1	-1	0
HT18	2	-3	0	1
HT19	0	-5	-1	0
HT20	1	-4	0	1
HT- MODEL_01	0	12	1	3
HT- MODEL_02	-3	-5	0	-3
HT- MODEL_03	-1	-6	0	-1
HT- MODEL_04	2	-6	2	2
HT- MODEL_05	4	0	5	3
HT- MODEL_06	1	-5	-2	-2
HT- MOD_BIS_01	-3	1	-1	-2
HT- MOD_BIS_02	-1	4	1	-3
HT- MOD_BIS_03	-2	7	-1	2

HT- MOD_BIS_04	-3	-1	-1	-1
HT- MOD_BIS_05	-3	-4	1	3
HT- AspectRatio_01	-4	-8	1	-1
HT- AspectRatio_02	12	11	1	1
HT- AspectRatio_03	2	45	0	13

References

1. V. Lavric; R. Isopescu; V. Maurino; F. Pellegrino; L. Pellutiè; E. Ortel; V.-D. Hodoroaba, *Crystal Growth & Design* **2017,**
2. aA. I. Vogel; G. H. Jeffery, *Vogel's Textbook of Quantitative Chemical Analysis*. Longman Scientific & Technical: Harlow, Essex, England, **1989**; bM. M. A. Perik; P. J. D. Oranje, *Analytica Chimica Acta* **1974,** *73,* 2
3. S. W. Provencher, *Computer Physics Communications* **1982,** *27,* 3
4. V. D. Hodoroaba; D. Akcakayiran; D. O. Grigoriev; D. G. Shchukin, *Analyst* **2014,** *139,* 8
5. P.-J. De Temmerman; E. Verleysen; J. Lammertyn; J. Mast, *Powder Technology* **2014,** *261,*
6. C. A. Schneider; W. S. Rasband; K. W. Eliceiri, *Nature Methods* **2012,** *9,* 7
7. aS. Hansen, *Journal of Chemical Physics* **2004,** *121,* 18 ; bF. Perrin, *Journal de Physique et le Radium* **1936,** *7,* 1
8. N. I. A.-S. Tim Kemmitt, Graeme J. Gainsford, *Inorg. Chem.* **2000,** *39,*
9. J.-J. M. Jin-Koo Park, Jin-Burm Kyong, Ho-Kun Kim, *Bull. Korean Chem. Soc.* **2003,** *24,* 5
10. G. E. P. Box, Wilson, K.B., , *J. Royal Stat. Soc.* **1951,** *1,*
11. aK. Y. Lee; N. Chung; S. Hwang, *Ecological Informatics* **2016,** *36,* ; bC. R. Gutierrez, *Advances in Knowledge Representation*. InTech: Rijeka, Croatia, **2012**.
12. aI. N. Kuznetsova; V. Blaskov; I. Stambolova; L. Znaidi; A. Kanaev, *Materials Letters* **2005,** *59,* 29–30 ; bH. Xie; N. Li; B. S. Liu; J. J. Yang; X. J. Zhao, *J Phys Chem C* **2016,** *120,* 19
